# Tracking early lung cancer metastatic dissemination in TRACERx using ctDNA

**DOI:** 10.1038/s41586-023-05776-4

**Published:** 2023-04-13

**Authors:** Christopher Abbosh, Alexander M. Frankell, Thomas Harrison, Judit Kisistok, Aaron Garnett, Laura Johnson, Selvaraju Veeriah, Mike Moreau, Adrian Chesh, Tafadzwa L. Chaunzwa, Jakob Weiss, Morgan R. Schroeder, Sophia Ward, Kristiana Grigoriadis, Aamir Shahpurwalla, Kevin Litchfield, Clare Puttick, Dhruva Biswas, Takahiro Karasaki, James R. M. Black, Carlos Martínez-Ruiz, Maise Al Bakir, Oriol Pich, Thomas B. K. Watkins, Emilia L. Lim, Ariana Huebner, David A. Moore, Nadia Godin-Heymann, Anne L'Hernault, Hannah Bye, Aaron Odell, Paula Kalavakur, Fabio Gomes, Akshay J. Patel, Elizabeth Manzano, Crispin T. Hiley, Nicolas Carey, Joan Riley, Daniel E. Cook, Darren Hodgson, Daniel Stetson, J. Carl Barrett, Roderik M. Kortlever, Gerard I. Evan, Allan Hackshaw, Robert D. Daber, Jacqui A. Shaw, Hugo J.W.L. Aerts, Abel Licon, Josh Stahl, Mariam Jamal-Hanjani, TRACERx consortium, Nicolai J Birkbak, Nicholas McGranahan, Charles Swanton

**Affiliations:** 1 Cancer Research UK Lung Cancer Centre of Excellence, University College London Cancer Institute, London, UK; 2 Cancer Evolution and Genome Instability Laboratory, The Francis Crick Institute, London, UK; 3 Invitae, San Francisco, California, USA; 4 Department of Molecular Medicine, Aarhus University Hospital, Aarhus, Denmark; 5 Artificial Intelligence in Medicine (AIM) Program, Mass General Brigham, Harvard Medical School, Boston, MA, USA; 6 Department of Radiation Oncology, Brigham and Women's Hospital, Dana-Farber Cancer Institute, Harvard Medical School, Boston, MA, USA; 7 Advanced Sequencing Facility, The Francis Crick Institute, London, UK; 8 Cancer Genome Evolution Research Group, Cancer Research UK Lung Cancer Centre of Excellence, University College London Cancer Institute, London, UK; 9 Tumour Immunogenomics and Immunosurveillance Laboratory, University College London Cancer Institute, London, UK; 10 Bill Lyons Informatics Centre, University College London Cancer Institute, London, UK; 11 Department of Cellular Pathology, University College London Hospitals, London, UK; 12 AstraZeneca, Cambridge, UK; 13 AstraZeneca, Waltham, MA, USA; 14 The Christie NHS Foundation Trust, Manchester, UK; 15 University Hospital Birmingham NHS Foundation Trust, Birmingham, UK; 16 Cancer Research Centre, University of Leicester, Leicester, UK; 17 Department of Biochemistry, University of Cambridge, Cambridge, UK; 18 Cancer Research UK & UCL Cancer Trials Centre, London, UK; 19 Radiology and Nuclear Medicine, CARIM & GROW, Maastricht University, Maastricht, The Netherlands; 20 Cancer Metastasis Laboratory, University College London Cancer Institute, London, UK; 21 Department of Oncology, University College London Hospitals, London, UK; 22 Department of Radiology, Freiburg University Hospital, 79106 Freiburg, Germany; 23 Department of Clinical Medicine, Aarhus University, Aarhus, Denmark; 24 Bioinformatics Research Centre, Aarhus University, Aarhus, Denmark

## Abstract

Circulating tumour DNA (ctDNA) can detect and profile residual tumour cells persisting after curative intent therapy^1^. Large patient cohorts incorporating longitudinal plasma sampling and extended follow-up are required to determine the role of ctDNA as a phylogenetic biomarker of relapse in early-stage non-small-cell lung cancer (NSCLC). Here, we developed ctDNA methods tracking a median of 200 mutations identified in resected NSCLC tissue across 1069 plasma samples collected from 197 patients enrolled in the TRACERx study^2^. Lack of preoperative ctDNA detection distinguished biologically indolent lung adenocarcinoma with good clinical outcome. Postoperative plasma analyses were interpreted within the context of standard-of-care radiological surveillance and administration of cytotoxic adjuvant therapy. Landmark analyses of plasma samples collected within 120 days post-surgery revealed ctDNA detection in 25% of patients, including 49% of all patients who experienced clinical relapse; 3 to 6 monthly ctDNA surveillance identified impending disease relapse in an additional 20% of landmark negative patients. We developed a bioinformatic tool (ECLIPSE) for non-invasive tracking of subclonal architecture at low ctDNA levels. ECLIPSE identified patients with polyclonal metastatic dissemination, which was associated with poor clinical outcome. Through measuring subclone cancer cell fractions in preoperative plasma, we found subclones seeding future metastases were significantly more expanded compared to non-metastatic subclones. Our findings will support (neo)adjuvant trial advances and provide new insights into the process of metastatic dissemination using low ctDNA level liquid biopsy.

ctDNA is a multi-faceted biomarker, pre-surgical ctDNA levels reflect relapse risk in early-stage NSCLC^3–5^ and postoperative ctDNA detection highlights impending NSCLC recurrence^4–8^. Potential exists for postoperative ctDNA to guide adjuvant therapy administration^9,10^. Longitudinal measurements of clonal composition across metastatic sites can also be captured using ctDNA^7,11–13^. Within the TRACERx study^2^ patients undergoing resection of NSCLC are managed according to National Institute of Clinical Excellence approved care pathways^14^ and followed for 5 years post-surgery. Plasma is collected preoperatively and at 3-monthly postoperative intervals in the first 2 years, followed by 6-monthly intervals between years 3 to 5. Previously, we demonstrated that 13/14 patients with NSCLC recurrence had detectable postoperative ctDNA which could provide insight into the clonal structure of residual disease^7^. Here, we analyse 1069 plasma samples from 197 patients with a median follow-up of 4.6 years in event-free patients. We implement new phylogenetic tracking technologies including patient-specific anchored-multiplex PCR (AMP)^15^ cell-free DNA (cfDNA) enrichment tracking a median of 200 tumour mutations, combined with an informatic tool (ECLIPSE) to extract clonal composition in the context of the low ctDNA levels (<1%) encountered in early-stage NSCLC^16^. We address prognostic implications of preoperative ctDNA detection, alongside postoperative ctDNA detection as an indicator of both impending disease relapse and phylogenetic pattern of metastatic dissemination.

## ctDNA detection using AMP

**Extended Figure 1:**
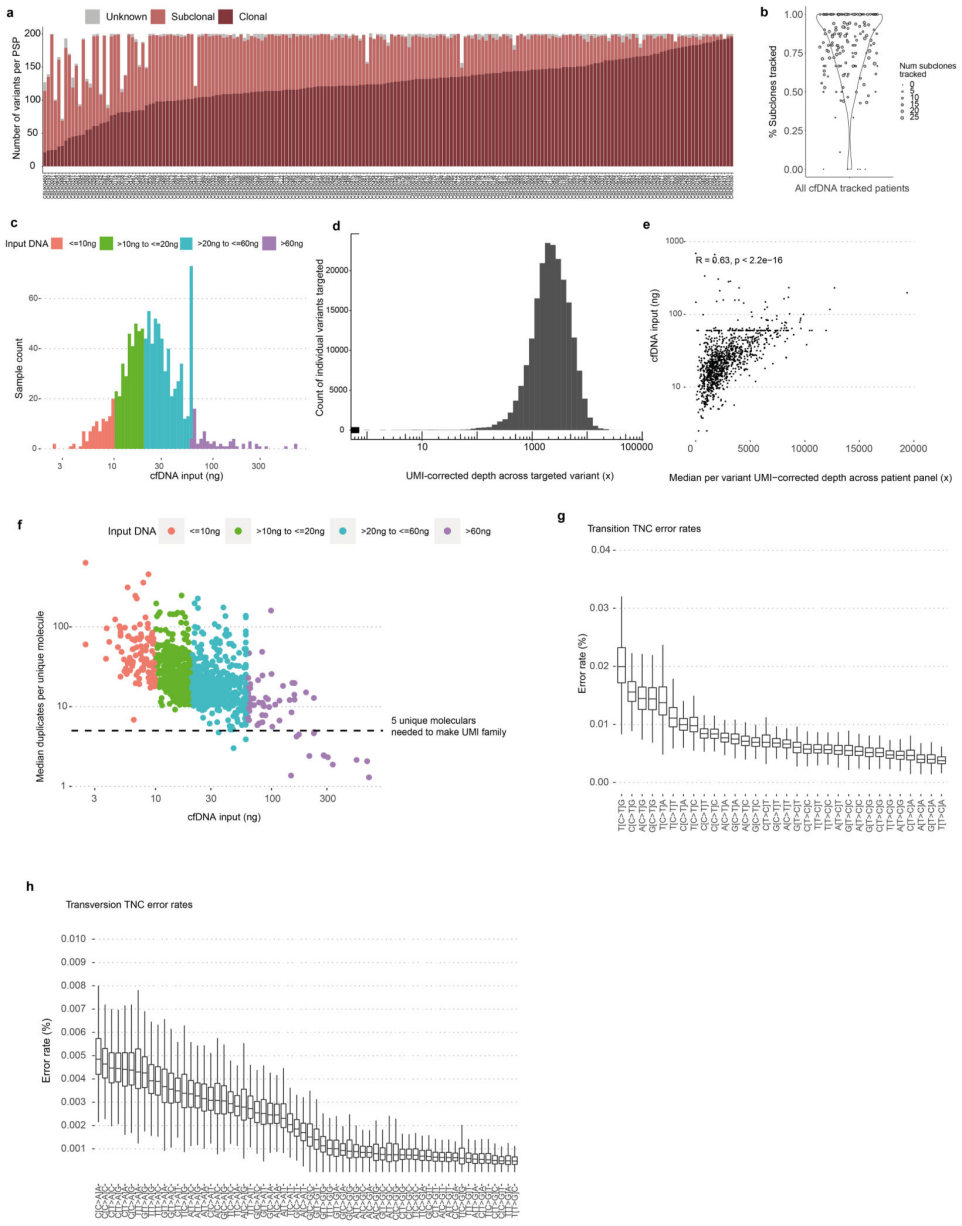
TRACERx ctDNA cohort sequencing parameters. **A**. Stacked bar plot of patient specific panels (PSPs) designed from primary tumour sequencing data showing the number of clonal (dark red) and subclonal (light red) variants per panel. Variants lacking clonality information are displayed in grey (median of 3 variants per patient [1-20], these mutations are either no longer called by TRACERx or called by ArcherDx but not TRACERx, see methods). A median of 126 clonal variants (range 21 to 195) and 64 subclonal variants (range 0 to 174) were tracked by the PSPs. Clonality was determined by PyClone analyses of multi-region exome data derived from primary resections of NSCLC (methods), in the absence of PyClone data, variants present in all multi-region sequenced tumour samples were called clonal. **B**. Violin plot demonstrating the % of subclonal clusters derived from multi-region tumour exome data tracked by PSPs on a per patient basis. A median of 88% of the subclonal mutation clusters present in each patient's multi-region exome derived phylogenetic tree were tracked [range 0-100]. 184 tumours with phylogenetic trees were included. **C**. Distribution of cfDNA input values for the cohort, median input of 23ng, n=1069 samples. Capping at 60ng input was performed for some of the cohort explaining the peak at this value; for the remainder of the cohort, all cfDNA extracted was input into the assay (colours represent different cfDNA input categories as indicated). **D**. Histogram demonstrating the distribution of per-variant unique sequencing depth values across the cohort; unique depth refers to error-controlled depth achieved across a position targeted by a PSP (at least 5 unique molecular identifier (UMI) matched reads required to create a consensus error-controlled read, see methods). The median unique depth per-variant tracked by a PSP was 2226x (range 0 to 53789x, n=201910). **E**. Correlation between cfDNA input (ng, Y axis) into the assay and the median UMI-corrected depth achieved across a PSP across 1069 plasma timepoints (X axis). Spearman's R value = 0.63 and two-sided P value < 2.2e-16. **F**. Association between median deduplication ratio achieved in a sample (Y-axis) and cfDNA input into the assay (ng, X-axis); duplication ratio refers to the median number of duplicate UMI-supported reads within a read family. Resequencing of samples where the median duplication ratio was less than 10 was performed where possible to maximise recoverable information from cfDNA samples, given that 5 UMI-supported reads are required to make a UMI family. 17 of 1069 evaluated cfDNA samples exhibited a final median deduplication ratio less than 5 (corresponds to the horizontal line on the plot). Colours correspond to different cfDNA input categories and match panel ©. **G-H**. Boxplots demonstrating the error rates (%, Y axis) per each of 96 mutation trinucleotide contexts (X axis, 192 mutation trinucleotide contexts [TNCs] simplified to 96 reverse-complement identical mutation types), plots divided by transition event (G) and transversion event (H). Background position data from n=1069 cell-free DNA libraries utilised to generate plots, variants predicted to exhibit low background error rates from pilot data analyses were prioritised for PSP design. Hinges correspond to first and third quartiles, whiskers extend to the largest/smallest value no further than 1.5x the interquartile range. Centre lines represent medians.

**Extended Figure 2:**
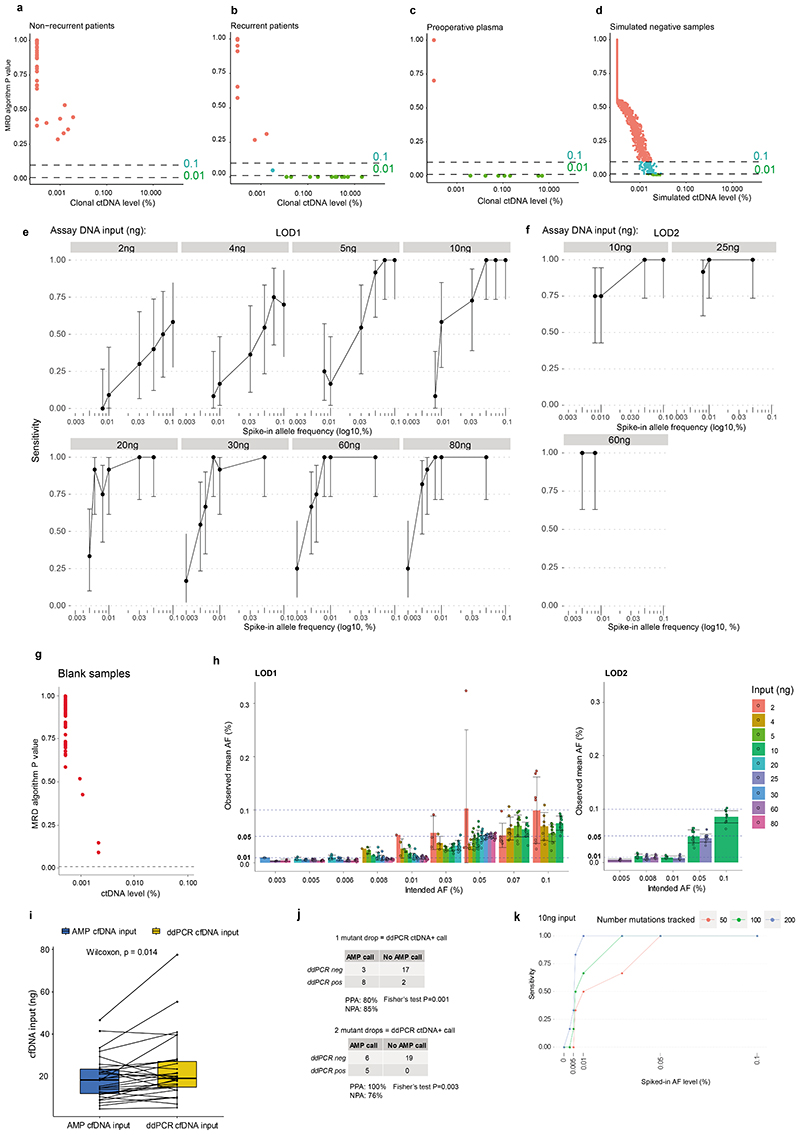
MRD calling thresholds and analytical validation. **A-D**. Postoperative MRD caller P values (one-sided Poisson test, see methods) observed in pilot-phase of the project. **A**. n=5 patients who did not have recurrence of their NSCLC; all n=55 patient samples had caller P values in excess of P > 0.1 threshold meaning that they were deemed negative for ctDNA. **B**. Postoperative caller P values observed in n=5 patients who had relapse of their NSCLC. 1 of 13 calls was made between caller P values of 0.1 and 0.01, the remaining 12 calls were made at a caller P value less than 0.01. **C**. Preoperative ctDNA calls from pilot cohort; 7 patients had positive ctDNA in plasma prior to surgery, all calls were made at caller P values <0.01. **D**. In-silico simulation analysis to assess MRD caller specificity. 3157 mock MRD panels were generated within the evaluable pilot patient libraries and MRD caller P values were assessed. At a caller P value <0.1 threshold, 121/3157 simulated mock panels were ctDNA positive (*in-silico* specificity of 96.2%); at a caller P value threshold <0.01, 22/3157 simulated mock panels were ctDNA positive (*in-silico* specificity of 99.3%). **E-F**. Analytical validation of 50 variant MRD detection panels. **E**. Fragmented DNA with a known single nucleotide polymorphism (SNP) profile was spiked into a second background of fragmented DNA with a different SNP profile and a patient-specific panel targeted 50 alternate positions present in spiked-in DNA. 559 data points were generated across different DNA input quantities indicated, to establish the limit of detection plots. The Y axis and centre of the error bars demonstrate sensitivity (defined as the proportion of all repeats that resulted in MRD detection using a caller P value of 0.01). The confidence intervals on the plot are Clopper-Pearson confidence intervals (95% CIs). The X axis shows the quantity of variant germline DNA that was spiked into each repeat expressed as a percentage of total DNA in that sample. **F**. Circulating tumour DNA samples with high variant allele fractions were spiked into a different cell-free DNA background. Variant positions in ctDNA were targeted with a 50 variant panel; 100 data points were generated across the DNA input quantities indicated. Axes and error bars are the same as (E). **G**. Data from analyses of 48 blank samples donated by 24 healthy participants, caller P values are displayed. **H**. Barplots demonstrating the intended allele frequencies and the measured allele frequencies in the different spike-ins presented in part (E) and part (F) only data from variant DNA positive samples are presented. The colours of the barplot represent different DNA input masses as shown by the legend. The error bars on the plot represent the mean value of all positive spike-in samples +/- standard deviation of the values. Where the error bar is absent, this is because at this spike-in level and DNA input mass, only one positive sample was observed. Where the error bar led to an observed mean AF less than 0, the error bar was stopped at 0 for visualisation purposes (the 0.05% spike-in, 2ng input mass case). The horizontal dashed lines correspond to 0.1%, 0.05%, and 0.01% spike-in categories. Each data point is represented on the plots by a circle. n=369 variant DNA positive samples displayed in LOD1 barchart, n=93 variant DNA positive samples displayed in LOD2 barchart. **I**. Comparison between the content of cell-free DNA input into ddPCR reactions (yellow) and AMP PCR reactions (blue). Hinges correspond to first and third quartiles, whiskers extend to the largest/smallest value no further than 1.5x the interquartile range. Centre lines represent medians. Each dot on the plot represents a data point, lines connect paired samples from the same patient. Significantly more cell-free DNA was input into ddPCR reactions (paired two-sided Wilcoxon-test P=0.01366). **J**. Orthogonal comparison between ctDNA detection based on AMP panels used in TRACERx and ddPCR against a single clonal variant. ddPCR ctDNA positive call threshold was two mutant droplets (bottom table) and one mutant droplet (top table). Percentage positive agreement (PPA) and percentage negative agreement (NPA) using ddPCR as the comparator is displayed in the table. Two-sided Fisher's test P values are demonstrated under the cross tables. **K**. A 300 mutation patient-specific panel was designed and applied to 10ng DNA samples containing spike-in variant levels from 0% to 0.1%. *In silico* sub-sampling of the 300 mutations was performed (3 x 200 mutation *in silico* panels, 3x 100 mutation *in silico* panels and 3x 50 mutation *in silico* panels, see methods) and sensitivities are categorised by the number of mutations targeted by the panel.

**Figure 1:**
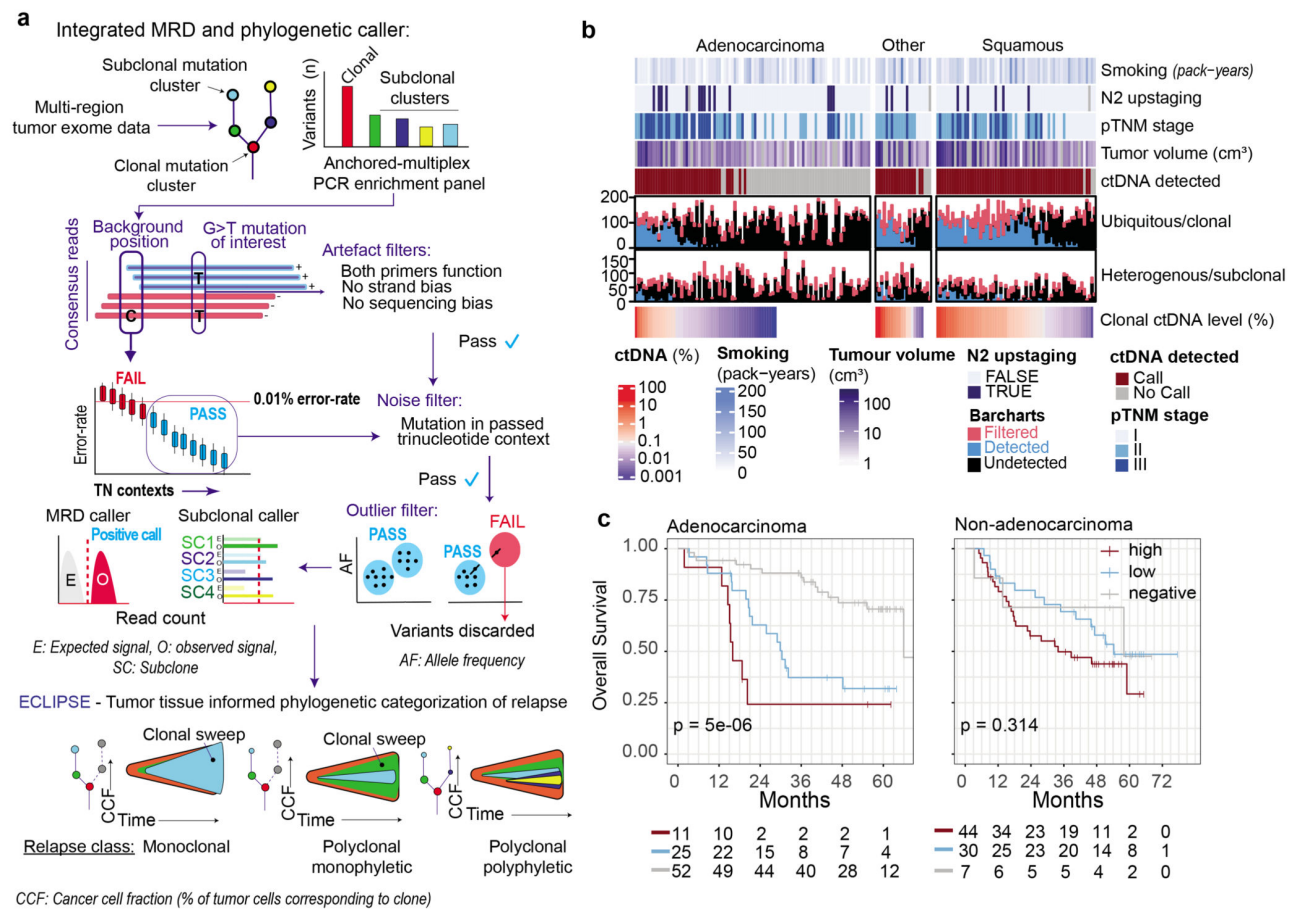
Overview of cohort and ctDNA calling. **A**. The ctDNA detection method estimates intra-library, trinucleotide specific sequencing error rates. For calling ctDNA, the number of consensus reads at all positions targeted by a patient specific panel (PSP), that pass described filters are compared to expected error rates. To detect subclones, ECLIPSE evaluates the collective signal across all mutations in each subclone and integrates this with primary-tumour derived copy number information to estimate plasma cancer cell fractions (CCF), clonal sweeps (where a subclone reaches 100% CCF) and metastatic dissemination patterns. The ctDNA analysis approach is described further in Supplementary Note. **B**. Heatmap of clinical features associated with preoperative ctDNA analyses in non-pilot TRACERx patients (with non-synchronous primary tumours). N2 upstaging row: patients clinically staged with N0/1 lymph-node involvement upstaged to N2 disease by pathology; grey - no pathology staging. pTNM stage row: pathological tumour node metastasis (v7) stage. Volumetrics row: tumour volume (cm^3^) measured by computed tomography, grey - unevaluable, log10 transformed. Barcharts: mutations tracked by a patient's PSP categorised by clonality; black - mutation undetected (per-variant one-sided Poisson P value >0.01, methods), red - mutation filtered by MRD caller, blue - mutation detected. Clonal ctDNA level: the mean percentage of mutant consensus reads across all clonally mutated positions tracked by a PSP (log10 transformed, methods), patients with 0% level are given a white colour, a non-zero clonal ctDNA level can occur in ctDNA negative patients where signal was insufficient to result in confident detection of ctDNA. **C**. Kaplan-Meier curves demonstrating overall survival outcomes in ctDNA high (dark red), ctDNA low (blue) and ctDNA negative (grey) non-synchronous adenocarcinoma patients (left) and non-synchronous non-adenocarcinoma patients (right). ctDNA high and low was categorised based on median clonal ctDNA levels across all ctDNA positive NSCLCs (0.16%). Log-rank P values displayed.

AMP patient-specific cfDNA enrichment panels (PSPs) targeted a median of 200 mutations pre-identified in multi-region exome analyses of early-stage NSCLC surgical resection specimens (range 72 to 201). A median of 126 clonal mutations were tracked, enabling sensitive identification of ctDNA; a median of 64 subclonal mutations (representing a median of 88% of subclones sampled in surgical tissue) were tracked to infer subclonal evolution at relapse (Figure 1a, Extended figure 1a-b, Supplementary Table 1). The median cfDNA input into the AMP assay was 23ng (interquartile range 15ng to 37ng, Extended figure 1c, Supplementary Table 2). A molecular residual disease (MRD) detection algorithm evaluated background (non-variant) sequencing positions to estimate library error rates, to enable ctDNA detection (methods, Figure 1a, Extended figure 1d-h, Supplementary Note). An MRD algorithm P value of 0.01 was determined optimal through analyses of a 10-patient pilot cohort (Supplementary Note, Extended figure 2a-d). Pilot patients were excluded from subsequent ctDNA analyses apart from ECLIPSE interrogation of subclonal kinetics (methods).

Analytical and orthogonal validation of variant DNA detection using the locked-assay was performed (Supplementary Note). 659 spike-in samples were analysed at assay DNA inputs of 2ng to 80ng and variant DNA levels of 0.003% to 0.1% (methods). Sensitivity for variant DNA detection using a 50-variant PSP at 0.01% variant DNA level (representative of ctDNA levels encountered post-resection of NSCLC, using current MRD assays^8^) was >90% at DNA inputs of 20ng and above. Below 20ng input, sensitivity for 0.01% variant DNA declined. Specificity was 100% in analyses of 48 healthy participant samples (Extended figure 2e-h, Supplementary Table 3). Orthogonal validation of preoperative ctDNA positive calls was performed using digital droplet PCR (Extended figure 2i-j, Supplementary Table 4). Tracking more than 50 mutations improved assay sensitivity at lower DNA inputs (Extended figure 2k, Supplementary Tables 5–6).

## Features of preoperative ctDNA detection

**Extended Figure 3:**
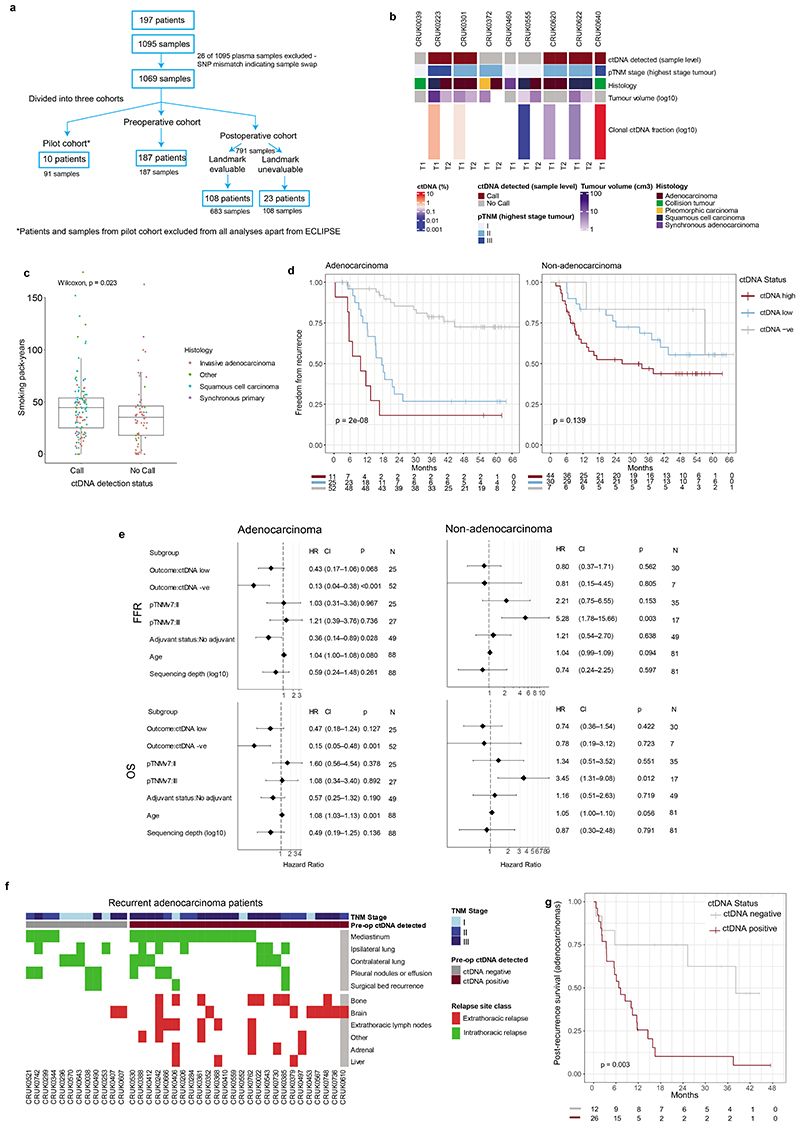
Preoperative ctDNA detection **A**. Flow diagram demonstrating different cohorts analysed in this manuscript; the top part of the flow diagram shows the total number of plasma samples that were intended to be analysed (n=1095 from 197 patients) which reduced to 1069 samples due to single nucleotide polymorphism mismatches between cfDNA and tissue exome data in 26 cases, suggesting sample swap. These samples were analysed in 3 main cohorts, the pilot cohort (left), the preoperative cohort (middle), and the postoperative cohort (right). The postoperative cohort was divided into different categories based on landmark evaluability (relating to samples donated within 120 days of surgery to enable a landmark ctDNA analysis). **B**. Heatmap demonstrating individual tumour-specific clonal ctDNA fractions in patients with synchronous primaries diagnosed at baseline. The annotation rows of the heatmap show the ctDNA call present in that sample across all variants interrogated by the MRD caller, the highest pathological TNM stage, the individual histology, and individual tumour volumes of the two synchronous tumours present at baseline (for this category, grey represents absent data or volume unevaluable). **C**. Boxplot demonstrating the difference in pack-year history across 187 preoperative ctDNA positive NSCLC patients and preoperative ctDNA negative NSCLC patients. Hinges correspond to first and third quartiles, whiskers extend to the largest/smallest value no further than 1.5x the interquartile range. Centre lines represent medians. P value represents a Wilcoxon-test. **D**. Kaplan-Meier curves demonstrating freedom from recurrence outcomes in ctDNA high (dark red), ctDNA low (blue), and ctDNA negative (grey) single primary adenocarcinoma patients (left) and single primary non-adenocarcinoma patients (right). ctDNA high and low were categorised based on median clonal ctDNA levels across ctDNA positive cases and relate to above and below 0.16%. Log-rank P values are displayed on each plot. **E**. Multivariable Cox regression analyses of Overall Survival (OS) and Freedom From Recurrence (FFR, defined as recurrence only) in patients with single (non-synchronous) NSCLC; evaluating ctDNA detection status, pTNM stage (Tumour Node Metastasis pathological stage version 7, categories I, II or III), whether adjuvant therapy was administered, age, and log10-transformed unique sequencing depth as predictors in adenocarcinomas and non-adenocarcinomas separately. Unique sequencing depth was included to adjust for under sequenced samples, representing potential false negatives. n=88 adenocarcinoma patients and n=81 non-adenocarcinoma patients were analysed for FFR and OS. On the forest plots, the diamond represents the multivariable Hazard Ratio (HR) with error-bars corresponding to 95% confidence intervals (CI). Multivariable P values (p) are displayed on the plot alongside the number of patients in each category (N). Reference categories were ctDNA positive patients, pTNM stage I patients and patients given adjuvant therapy. The exact Cox regression P value for the Outcome: ctDNA -ve category in the FFR adenocarcinoma plot = 0.00022. **F**. Heatmap showing the site of relapse in recurrent adenocarcinoma cases divided by whether preoperative ctDNA was detected (dark red, right) or undetected (grey, left). Intrathoracic (mediastinum, locoregional, ipsilateral lung, distant lung – green colours) or extrathoracic (bone, brain, liver, adrenal, extrathoracic lymph nodes or other extrathoracic site – red colours) sites of relapse are shown (sites shown are metastatic sites diagnosed within 180 days of clinical relapse). Heatmap is annotated by Tumour Node Metastasis pathological version 7 stage. **G**. Kaplan-Meier curve demonstrating post-relapse survival in recurrent adenocarcinoma patients stratified by preoperative ctDNA positive (red) or preoperative ctDNA negative (grey). Log-rank P value is displayed on the plot.

Preoperative cfDNA was analysed across 187 TRACERx patients (Supplementary Table 7–8, Extended figure 3a). 178 patients had a single primary NSCLC (Figure 1b) and 9 patients had synchronous primary NSCLCs at diagnosis (Extended figure 3b, Supplementary Note). In agreement with prior findings^3,7^, higher rates of preoperative ctDNA detection in non-adenocarcinoma histologies compared with lung adenocarcinoma were observed (39/93 lung adenocarcinomas ctDNA positive versus 78/85 non-adenocarcinomas, Figure 1b). Patients exhibiting preoperative ctDNA detection had a higher smoking pack-year history (Wilcoxon-test P=0.023, Extended figure 3c, Figure 1b). Preoperative ctDNA detection associated with clinically occult mediastinal lymph node disease in patients with adenocarcinoma. 81 adenocarcinomas were clinical N0/1 stage and following pathological nodal staging performed in 80/81 patients, 14/80 were upstaged to pN2 status. 11/14 (79%) pN2 upstaged patients were ctDNA positive versus 19 of 66 (29%) not upstaged (Chi-square test P=0.001, Figure 1b). Therefore, preoperative ctDNA detection could guide mediastinal resection strategies in adenocarcinoma.

## Preoperative ctDNA and clinical outcome

Given the variation in ctDNA detection across NSCLC subtypes, we assessed preoperative ctDNA status (negative [absent detection]; low, or high [classified based on clonal ctDNA level, the mean percentage of mutant consensus reads across clonally mutated positions tracked by a PSP]) as a prognostic biomarker separately in patients with single (non-synchronous) adenocarcinomas (n=88) and single non-adenocarcinomas (n=81) evaluable for survival analyses (methods). In patients with adenocarcinoma, ctDNA status was associated with overall survival (OS) (log-rank P=5e-06, Figure 1c). The 52 of 88 (59%) adenocarcinoma patients who were preoperative ctDNA negative had superior OS outcomes (90% 2-year OS [95% CI:82 to 99%]) compared with ctDNA low (63% 2-year OS [95% CI: 46 to 85%], n=25) or high adenocarcinoma (24% 2-year OS [95% CI: 8 to 74%], n=11). In non-adenocarcinoma, 7 of 81 ctDNA negative patients had OS outcomes indistinguishable from ctDNA low or high patients and ctDNA status was not strongly prognostic (log-rank P=0.314, Figure 1c, when the 7 ctDNA negative patients were excluded log-rank P=0.2). Similar findings were observed in freedom from recurrence (FFR) analyses (Extended figure 3d). In multivariable survival analyses including pathological TNM (pTNM) stage, adjuvant therapy status, age, and unique sequencing coverage, preoperative ctDNA status was associated with FFR and OS in adenocarcinoma but not in non-adenocarcinoma (Extended figure 3e). In patients with adenocarcinoma, preoperative ctDNA detection was associated with extrathoracic metastasis and poor post-recurrence outcomes. 18/20 (90%) recurrences involving extrathoracic sites occurred in patients who were preoperative ctDNA positive, compared with 8/18 (44%) intrathoracic-only recurrences (Chi-squared test, P=0.008); post-recurrence survival was shorter in those who were preoperative ctDNA positive relative to those who were preoperative ctDNA negative (log-rank P=0.003, Extended figure 3f-g).

## Biology of ctDNA detection

**Extended Figure 4:**
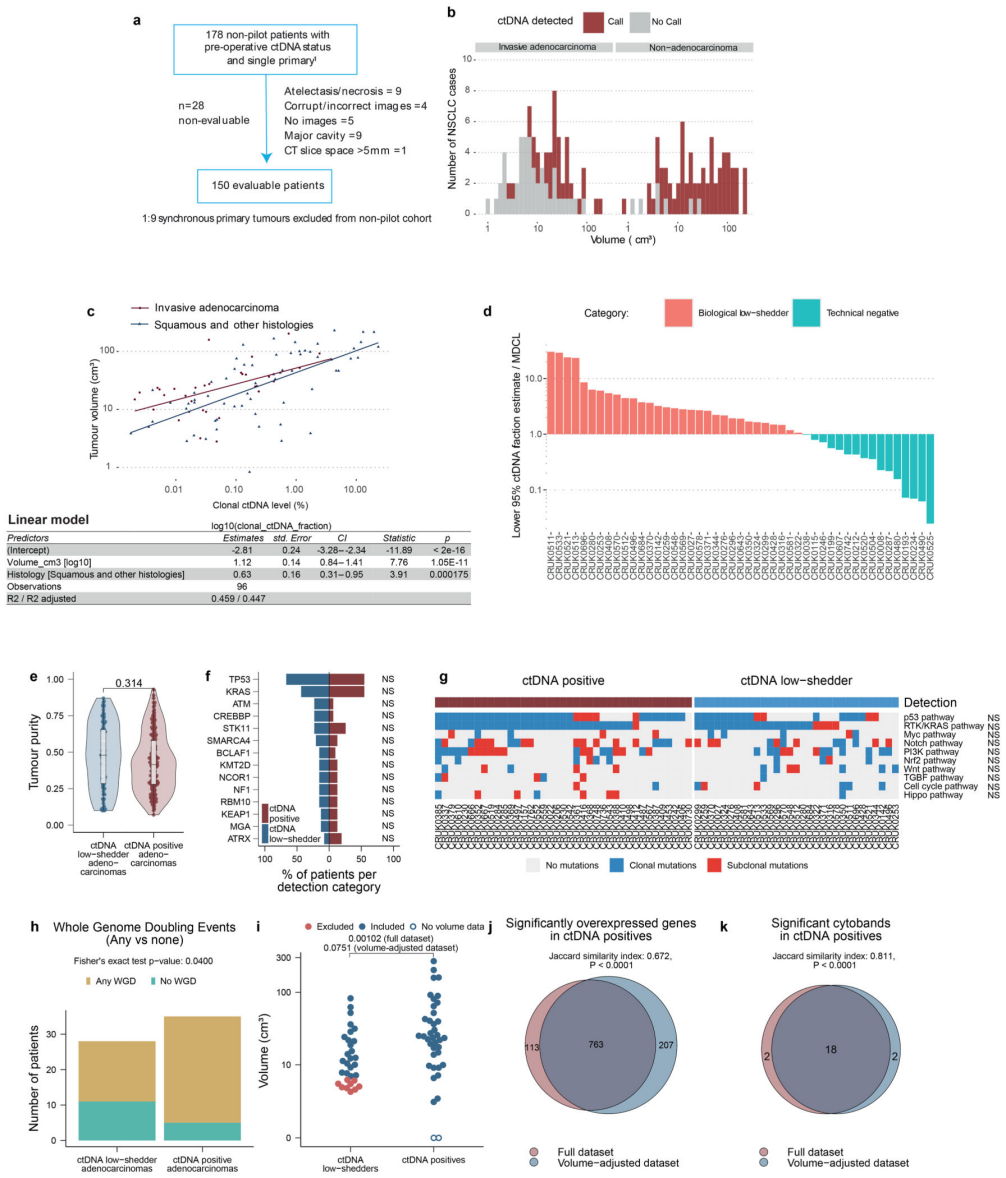
Volume and phenotypic analysis of ctDNA positive and ctDNA negative adenocarcinomas. **A**. Flow chart demonstrating patients available for volumetric analyses and reasons for exclusion. **B**. Histogram showing the number of NSCLC cases by volume, with ctDNA positive samples shown as red bars, and ctDNA negative samples shown as grey bars. n=150 volume evaluable cases. **C**. Volume versus log10-transformed clonal ctDNA level correlation plot with each individual TRACERx case that was ctDNA positive as a point and coloured by adenocarcinoma status (dark red) and squamous or other histology (dark blue). Fitted line represents a linear model line categorised by tumour histology. Below the correlation plot is a table describing a linear multivariable model based on these data to predict log10-transformed clonal ctDNA levels based on tumour volume and histology (adenocarcinoma and squamous and other categories). P values represent linear model adjusted P values, n=96 ctDNA positive, volume evaluable NSCLCs analysed. **D**. Based on a multivariable linear regression model fitted to the data in (C), we categorised ctDNA negative adenocarcinomas as biological low-shedders or technical non-shedders (see methods). If a particular tumour volume resulted in an estimated clonal mutation ctDNA level above the clonal ctDNA level a library could detect (95% lower confidence interval for estimated clonal ctDNA level based on tumour volume is above detectable clonal ctDNA level in the preoperative cfDNA library from that patient), then the case was classed as a probable biological low-shedder (red on histogram); otherwise, the case was classed as a probable technical non-shedder (turquoise on histogram). Y axis represents the lower 95% confidence estimate for clonal mutation ctDNA level divided by the minimally detectable clonal mutation ctDNA level (MDCL) for that patient's panel. The X axis is each individual patient analysed. Data from n=47 ctDNA negative adenocarcinomas presented. **E**. Violin box-plots comparing tumour purity in ctDNA low-shedder adenocarcinomas (blue, n = 79 tumour regions from 28 patients) and ctDNA positive adenocarcinomas (red, n = 166 tumour and lymph node regions from 35 patients). Pairwise comparisons are performed using linear mixed-effects models, P values are two-sided. Boxplot hinges correspond to first and third quartiles, whiskers extend to the largest/smallest value no further than 1.5x the interquartile range and centre lines represent medians. Violins represent the distribution of the underlying data. **F**. Barplots showing gene-level driver alterations between ctDNA positive adenocarcinomas (n = 39 patients) and ctDNA negative low-shedder adenocarcinomas (n = 31 patients). Colours denote ctDNA detection status. Y axis shows the top 14 most frequently altered genes, X axis shows the percentage of patients carrying an alteration in the gene per detection category. NS: Not significant (two-sided Fisher's exact test with FDR P value adjustment). **G**. Pathway-level driver mutations between ctDNA positive adenocarcinomas (n = 39 patients) and ctDNA negative low-shedder adenocarcinomas (n = 31 patients). X axis shows patient IDs, Y axis shows pathways following the Sanchez-Vega definition. Top bar denotes ctDNA detection status (dark red represents ctDNA positives, blue represents biological low-shedders). Heatmap colours display mutations; blue denote clonal mutations and red denote subclonal mutations. No pathway showed significant enrichment in either ctDNA shedder or non-shedder adenocarcinomas (NS: Not significant, using two-sided Fisher’s exact test with FDR P value adjustment). **H**. Whole genome doubling status per tumour comparing ctDNA positive adenocarcinomas to ctDNA negative low-shedder adenocarcinomas, using two-tailed Fisher's exact test. Yellow represents the number of tumours subjected to whole genome doubling in at least one region, turquoise represents tumours without any whole genome doublings. **I**. Volume by ctDNA shedding status. Biological non-shedders in red represent the smallest quartile samples. After removal of these from the analysis, no significant difference in tumour volume was found between ctDNA positives and ctDNA low-shedders. Pairwise comparisons are made with two-sided Wilcoxon-tests. **J**. Venn diagram showing the overlap between significantly differentially expressed genes between ctDNA positive and ctDNA low shedder adenocarcinomas obtained from the full dataset, relative to the volume-adjusted dataset. Comparisons are made by computing the Jaccard similarity index and the corresponding two-sided P value using the exact method. **K**. Venn diagram showing the overlap between significantly altered cytobands as called by GISTIC, comparing ctDNA positive to ctDNA low shedder adenocarcinomas obtained from the full dataset, relative to the volume-adjusted dataset. Statistical testing follows (J).

Computed Tomography (CT) volumetric data were available for 150/178 patients with non-synchronous NSCLC (Extended figure 4a, Supplementary Table 8). In NSCLC, 10cm^3^ tumour volume has been associated with ctDNA levels of ~0.1%^3,7,17^ (a level detectable by AMP; Extended figure 2). 17/42 (41%) patients with adenocarcinoma and tumour volumes of ≥10cm^3^ were preoperative ctDNA negative, compared with only 2/50 (4%) patients with non-adenocarcinoma (chi-squared test, P <0.001, Extended figure 4b). The relative absence of ctDNA detection in higher-volume adenocarcinomas suggested a low-ctDNA shedding phenotype. We developed a regression model in 96 preoperative ctDNA positive cases to estimate clonal ctDNA levels based on tumour histology and volume (Extended figure 4c, methods). We then estimated clonal ctDNA levels in the 47 ctDNA negative adenocarcinomas categorising these tumours as low-shedders (ctDNA detection expected based on tumour volume, but not observed [31/47 cases]) or technical negatives (tumour volume predicted for ctDNA levels below sample limit of detection [16/47 cases], Extended figure 4d, methods). The latter group was excluded from analyses of ctDNA detection and tumour biology.

**Figure 2:**
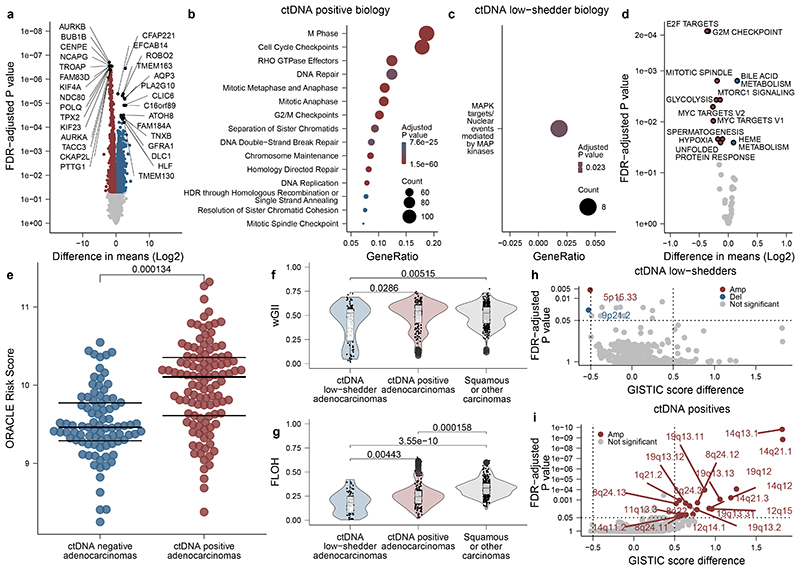
Genomic and transcriptomic predictors of ctDNA detection in early-stage NSCLC. **A**. Differential gene expression analysis comparing 34 ctDNA positive adenocarcinomas (101 regions) to 28 ctDNA low-shedder adenocarcinomas (62 regions). X axis shows log2 difference in means, Y axis shows two-sided FDR adjusted P values. Statistical testing is carried out by computing moderated t-statistics from a linear model fit to the transformed expression data (methods). Red and blue: genes significantly over-expressed in ctDNA positives and ctDNA low-shedders (technical non-shedders excluded), respectively. Top 15 genes are labelled per detection category. **B**, **C**. Reactome pathway enrichment analysis based on the 1,759 significant genes found in **A**. Y axis lists pathways, X axes shows proportion of genes involved. **B**. Top 15 pathways in ctDNA positives. **C**. The only significantly enriched pathway in ctDNA low-shedders. Size: gene count, colour: one-sided hypergeometric P value. **D**. Differential enrichment analysis based on the Hallmark gene-sets. Samples, axes and colours follow **A**. **E**. ORACLE gene expression scores in ctDNA positive (35 patients, 109 regions) versus ctDNA negative (42 patients, 87 regions) adenocarcinomas. Centre lines show medians. Colours follow **A**. **F, G**. Violin-boxplots showing wGII and FLOH levels of ctDNA positive adenocarcinomas (35 patients, 166 regions), ctDNA low-shedder adenocarcinomas (28 patients, 79 regions) and squamous or other carcinomas (74 patients, 303 regions). Hinges correspond to first and third quartiles, whiskers extend to the largest/smallest value no further than 1.5x the interquartile range. Center lines represent medians. **H, I**. GISTIC score analysis comparing 35 ctDNA positives (166 regions) and 28 ctDNA low-shedders (79 regions). Red: amplifications, blue: deletions, grey: non-significant values. Y axis: one-sided P values computed by GISTIC 2.0's permutation-based statistical methods, X axis: GISTIC score difference. Dotted lines: G-score and significance cutoffs. Pairwise comparisons are performed using linear mixed-effects models, P values are two-sided.

Available multi-region transcriptomic data allowed comparison of 34 ctDNA-positive adenocarcinoma to 28 low-shedder adenocarcinomas (Figure 2a, Supplementary table 9-10). Genes upregulated in ctDNA positive adenocarcinomas included those associated with M-phase, cell cycle, and DNA repair (Supplementary table 11); and Gene Set Variation Analysis (GSVA^18^) using the Hallmark genesets (which summarise 50 biological states^19^) revealed upregulation of proliferation and cell cycle associated gene sets (Figure 2b-d). We evaluated our published prognostic biomarker associated with outcomes in lung adenocarcinoma (ORACLE^20^). Preoperative ctDNA-positive adenocarcinomas demonstrated higher ORACLE scores relative to negative adenocarcinomas (P = 0.000134, Figure 2e). We observed no difference between ctDNA positive adenocarcinoma and low-shedders when we analysed tumour purity and subclonal and clonal somatic driver mutations, individually and summarised to pathways (Extended figure 4e-g). We observed that ctDNA-positive adenocarcinomas showed increased levels of both wGII (weighted genome integrity index^21^) and FLOH (fraction of loss of heterozygosity^22^) relative to low-shedders (P = 0.0286 & P = 0.00443) and an increased percentage of ctDNA positive adenocarcinomas had experienced whole genome doubling (WGD, any WGD compared to none, 86% versus 61%, P = 0.0400, Figure 2f-g, Extended figure 4h). We used GISTIC2.0^23^ to assess if the increased levels of chromosomal alterations in ctDNA positive tumours were linked with the observed increase in cell proliferation (methods). We observed 20 amplified cytobands enriched in ctDNA shedders (FDR q-value < 0.05) with a GISTIC score difference (GSD) of at least 0.5 (Figure 2h-i), a previously defined threshold for comparing two sample sets^24^. Within these cytobands, a total of 966 genes are located, of which 21 are listed in the COSMIC cancer gene census^25^ as cancer genes (Supplementary table 12), including proliferation-associated genes *CCND1* (11q13.3), *CDK4* (12q14.1), *MDM2* (12q15) and *CCNE1* (19q12). These results were largely recapitulated when excluding the bottom quartile of tumour volumes from low-shedding adenocarcinomas (Supplementary Note, Extended figure 4i-k); indicating that tumour biology is likely the main driver behind our observations.

## Postoperative ctDNA detection without relapse

**Extended Figure 5:**
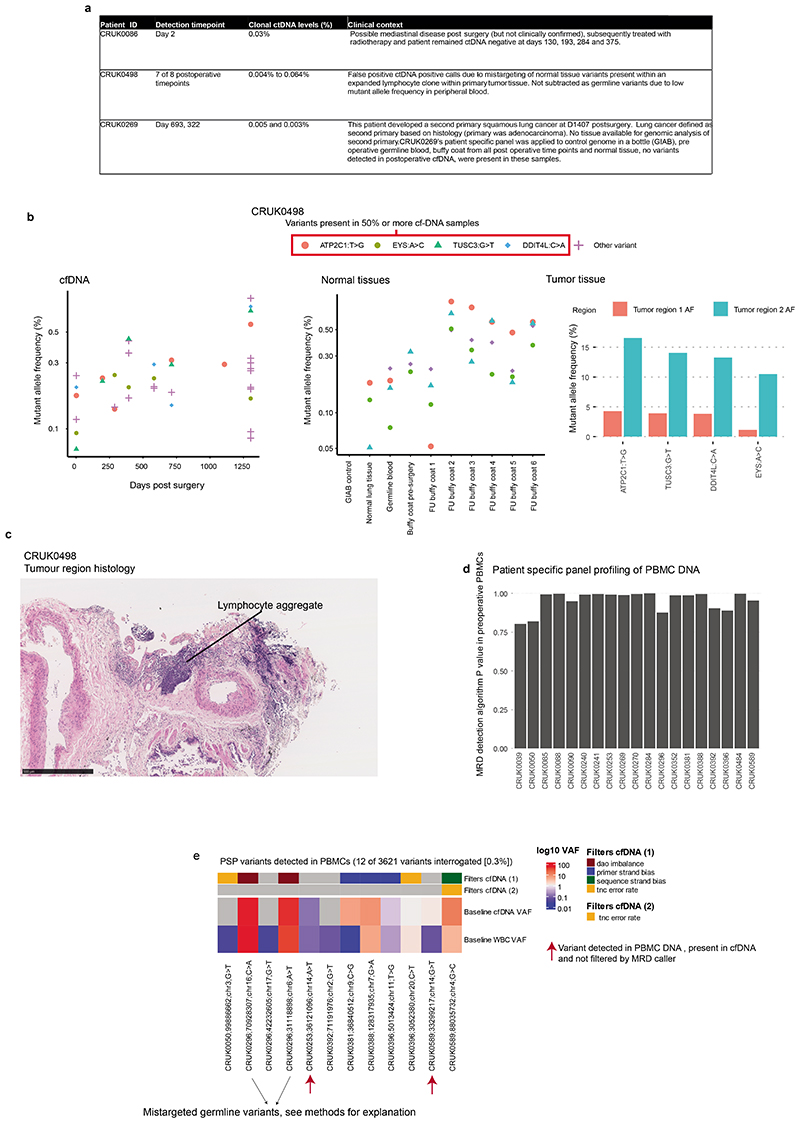
Exploration of unexpected MRD positive results in non-relapse patients. **A**. Table demonstrating details of unexpected ctDNA positive results in patients who did not suffer disease recurrence. **B**. CRUK0498 false positive analysis: Dot-plots represent confidently detected variants at illustrated cfDNA sampling timepoints (left panel), variants confidently detected in normal tissue, control DNA, and peripheral-blood mononuclear cell (PBMC, buffy-coat) DNA based on application of CRUK0498's patient specific panel to these respective samples (middle panel) and the mutant allele frequencies of selected variants in tumour tissue exome data (right panel). The four variants in the legend (variants in genes *ATP2C1*, *DDIT4L*, *EYS*, and *TUSC3*) represent variants confidently called at 50% or more of the timepoints across the cfDNA samples (note that confidently called means an individual variant Poisson one-sided P value of <0.01 [generated by MRD caller, see methods]). **C**. A haematoxylin and eosin image from patient CRUK0498's tumour where exome analysis detected the variants in genes *ATP2C1*, *DDIT4L*, *EYS* at high variant allele-frequencies. This image shows a dense lymphocyte aggregate in this tumour region. Scale bar below image. A single image was analysed. **D**. A further 19 preoperative PBMC samples were analysed from TRACERx patients; no confident panel-wide variant DNA calls were made in these patients' PBMC samples using the MRD calling algorithm. **E**. Variant-level analyses of the preoperative samples analysed in panel (D) highlighted that 12 of 3621 variants interrogated by the panels were detected (variant level one-sided Poisson P value <0.01). 8 of 12 detected variants were removed from the MRD caller algorithm in cell-free DNA analyses (cfDNA) due to triggering filters highlighted in the heatmap annotation. Only 2 of the 4 remaining variants carried deep alternate reads in the respective patients' preoperative cfDNA sample (red arrows). The heatmap shows the cfDNA variant allele frequency and the WBC variant allele frequency of the detected variants (grey colour represents no detection of the variant). Two mistargeted germline variants are highlighted by black arrows for patient CRUK0296, variants were targeted in error by the industry panel design pipeline but not by the TRACERx exome pipeline (methods), and were filtered from the MRD calling algorithm due to triggering the outlier filter (dao imbalance filter, dark red).

**Figure 3:**
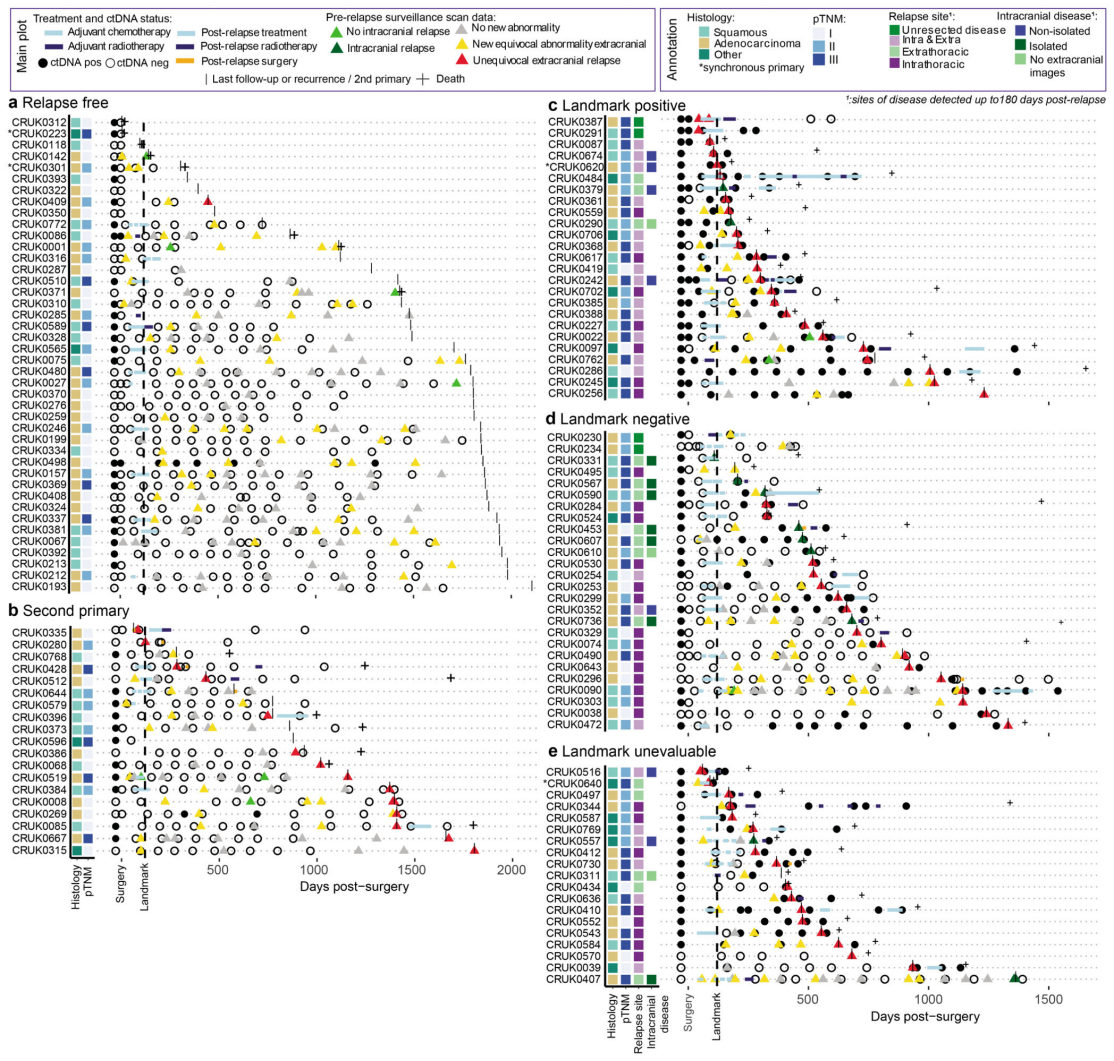
Postoperative Minimal Residual Disease detection in early-stage NSCLC. **A-D**. Longitudinal ctDNA data from non-pilot patients with (**A**) no evidence of non-small-cell lung cancer (NSCLC) recurrence, n=42; (**B**) development of a second-primary cancer, n=19; (**C**) recurrence of NSCLC in landmark positive patients, n=25 patients (**D**) recurrence of NSCLC in landmark negative patients, n=26 patients and (**E**) recurrence of NSCLC in landmark unevaluable patients, n=19 patients. In all panels, each circle represents a cfDNA sampling time point. Circles to the left of surgical day are preoperative timepoints, circles to the right of surgical day are postoperative timepoints. Black filled circle: positive ctDNA detection. Light blue rectangles: chemotherapy, dark blue rectangles: radiotherapy, orange rectangles: patient received post-recurrence surgery. Triangles represent standard of care postoperative CT, PET or MRI surveillance imaging (imaging up until first relapse or last follow-up displayed on plot). Imaging classified as no disease (grey), equivocal images (yellow), or unequivocal imaging evidence of extracranial relapse (red). Light green triangles: no evidence of intracranial relapse, dark green triangles: intracranial relapse. Vertical black lines: the event date for a patient (if death, second-primary, NSCLC recurrence occurred); otherwise, the vertical line represents last TRACERx follow-up. Crosses: patient death events. To the left of the panels, the annotation plots highlight histology, pTNM (pathological TNM) status, relapse site, and details regarding whether an intracranial relapse was isolated (brain-only) or non-isolated (brain and extracranial site) or occurred without extracranial imaging to confirm solitary status. Relapse site annotation displays anatomical sites of disease identified within an 180 day post-recurrence period.

Postoperative cfDNA samples from 42 recurrence-free patients and 19 patients who subsequently developed new primary cancers during follow-up (based on histological or clinical findings) were analysed to assess AMP clinical specificity (PSPs are specific to the excised NSCLC and are not expected to detect new primary cancers, Figure 3a-b, Supplementary Table 13). 10 of 426 (2%) postoperative samples from 3 of 61 (5%) of patients exhibited ctDNA detection (Figure 3a-b). CRUK0086 was ctDNA positive prior to radiation therapy, CRUK0269 was ctDNA positive post-surgery and developed a new primary NSCLC and CRUK0498 had false positive ctDNA detection at 7 of 8 postoperative timepoints likely due to PSP-mistargeting of somatic mutations associated with a lymphoid aggregate present in primary tumour tissue (Supplementary Note, Extended figure 5a-e).

## Postoperative ctDNA detection and relapse

**Extended Figure 6:**
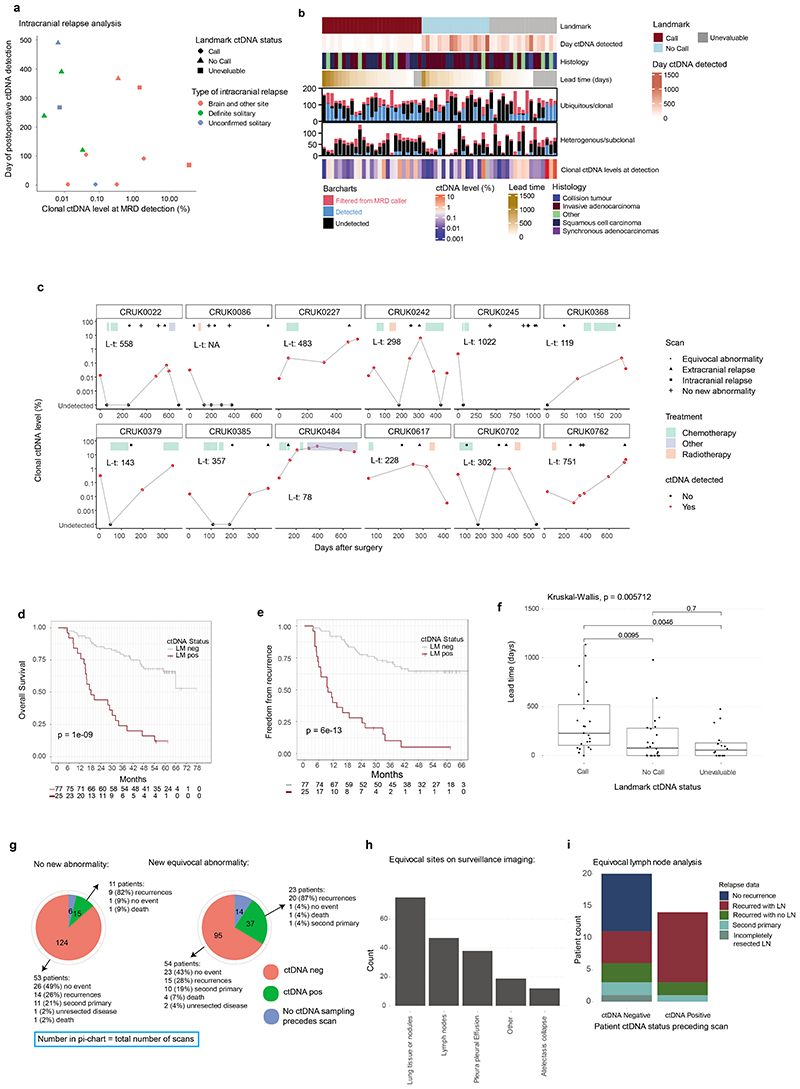
Expanded postoperative ctDNA and imaging surveillance analysis. **A**. Analysis of 13 patients who experienced intracranial relapse who were positive for ctDNA in a postoperative blood sample. The X axis shows the clonal ctDNA level at the point of postoperative ctDNA detection and the Y axis shows the day of postoperative ctDNA detection. Points are coloured based on whether the intracranial relapse was solitary (green), accompanied by another extracranial site (red), or unconfirmed solitary (blue, no extracranial imaging performed) and are shaped by landmark ctDNA status. **B**. Heatmap of clonal mutation ctDNA level data at first postoperative ctDNA detection. The annotation rows show the landmark ctDNA status of the patient (landmark positive, ctDNA detected within 120 days postoperatively; landmark negative, ctDNA negative within 120 days postoperatively; unevaluable, landmark status cannot be established), the day ctDNA was detected postoperatively, the histology of the primary tumour, and lead time (days from ctDNA detection to clinical relapse). Where lead time was not applicable (for example incompletely resected disease, ctDNA detected post-relapse, see methods) lead time is coloured grey. The next two rows (bar charts) demonstrate the number of clonal or subclonal mutations tracked by an AMP patient-specific panel (PSP); if the bar is blue, it represents confident detection of an individual variant (based on an individual variant P value of <0.01 [one sided Poisson test based on MRD caller output, see methods]), if the bar is black, it represents absence of confident calling of a variant, if the bar is red, it represents that a variant was filtered by the MRD calling algorithm. The final row represents the mean clonal ctDNA level at the first ctDNA detection time point for a patient. This is on a log-10 scale as displayed in the heatmap legend. For patient CRUK0296, ctDNA detection occurred but clonal ctDNA levels were 0% (grey bar) as the mutation driving ctDNA detection postoperatively did not have a clonal status. **C** Longitudinal per-patient plots in 12 patients who were ctDNA positive prior to adjuvant therapy. Plots are annotated with lead time (L-t), scans performed, and treatment administered (see legend). The Y axis represents clonal ctDNA levels and each circle on the plot represents a blood sampling time point. If the circle is red, it indicates that the blood sample was positive for ctDNA using the MRD caller. The X axis displays days post-surgery. **D-E**. Kaplan-Meier curves in the landmark evaluable population (patients who donated blood within 120 days post-surgery before treatment or clinical recurrence, n=102/108 landmark evaluable patients were evaluable for survival analysis, see methods for exclusions) showing overall survival (OS,D) or freedom from recurrence (FFR,E) outcomes for landmark positive (dark red) versus landmark negative (grey) patients. Log-rank P values displayed on curves. **F**. Boxplots showing the distribution of lead times (times from ctDNA detection to clinical recurrence) categorised by patient landmark ctDNA status. Hinges correspond to first and third quartiles, whiskers extend to the largest/smallest value no further than 1.5x the interquartile range. Centre lines represent medians. Kruskal-Wallis test P=0.0057, unadjusted pairwise Wilcoxon-tests compare individual categories, n=63 patients analysed. **G**. Pie charts demonstrate the number of occurrences of specified ctDNA detection statuses (red – ctDNA negative, green – ctDNA positive, blue – no ctDNA status established), preceding a scan showing no new changes (left) or new equivocal extracranial changes (middle). The ctDNA positive and negative categories are then broken down further into a patient-level analysis showing the outcomes of patients who experienced the occurrence of the specified imaging and ctDNA status event(s). **H**. Barchart showing the count of specific equivocal anatomical sites noted on scans showing new equivocal changes; equivocal lung lesions and lymph nodes were the most common abnormal equivocal findings on NSCLC surveillance imaging. Multiple equivocal sites can be observed on one scan. **I**. Barplot of eventual site of relapse and ctDNA status in 33 patients with ctDNA status established prior to surveillance imaging, showing new equivocal lymph node enlargement. The X axis shows the patient ctDNA detection status preceding surveillance scans. The Y axis shows the patient count. Patient CRUK0090 exhibited occurrences of both negative and positive ctDNA statuses prior to separate equivocal lymphadenopathy scans, so is present in both ctDNA positive and negative categories. Other patients are only included once. Patient CRUK0234 was diagnosed with an unresected lymph node, was ctDNA negative postoperatively and included in the analysis. The barcharts are filled with recurrence status of patients in these categories. Recurred with LN refers to lymph node involvement at relapse (dark red colour). Recurred with no LN refers to recurrence with no lymph node involvement (green colour).

365 postoperative plasma samples were analysed from 70 patients who suffered either recurrence of their NSCLC (n=66) or incomplete resection (macroscopic residual disease, n=4, Figure 3c-e, Supplementary Table 13). ctDNA was detected postoperatively (pre- or post-relapse) in 59/70 (84%) of these patients. 3/11 patients relapsing without postoperative ctDNA detection lacked plasma sampling within 100 days of clinical relapse (CRUK0303, 0495, 0570). In those with plasma sampled close to relapse, 2/11 patients had unresected hilar or mediastinal lymph-node metastases on postoperative imaging (CRUK0230, 0234), and 4/11 had intracranial recurrence (CRUK0331, 0407, 0567, 0736), and 2/11 had intrathoracic recurrence (CRUK0329, 0490, Figure 3c-e, Supplementary Table 14). Intracranial recurrence has previously been associated with absent postoperative ctDNA detection^26^. Here, 17 patients experienced brain metastases within 180 days of relapse and 14/17 patients also had extracranial imaging at relapse. Of these 14 patients, 3/7 patients with isolated (brain-only) intracranial relapse versus 7/7 with non-isolated intracranial relapse exhibited postoperative ctDNA detection (Figure 3c-e, Extended figure 6a).

## Landmark MRD analysis

We explored postoperative ctDNA detection within a landmark analysis framework^1,6^ (Figure 3a-d). 108/131 patients with postoperative plasma sampling performed were evaluable for landmark analysis based on ≥1 plasma sample obtained within 120 days of surgery, prior to adjuvant therapy or relapse (Supplementary Table 7). 51/108 patients relapsed, with disease recurrence (n=47) or incompletely resected disease detected during follow-up (n=4). At landmark, 27/108 patients (25%) exhibited 1 or more positive ctDNA calls and 25/27 of these patients relapsed (positive predictive value of landmark for relapse 93%, negative predictive value 68%, sensitivity of landmark for relapse 49%). Landmark positive status associated with higher pTNM stage (5/41 [12%] stage I, 8/35 [23%] stage II and 14/32 [44%] stage III patients landmark positive, chi-squared test P=0.008). 15/21 (71%) relapse events occurring within 1 year of surgery were landmark positive versus 8/26 (31%) events occurring later than 1 year (4 patients with incomplete resections excluded, chi-squared test P=0.01). The median clonal ctDNA level at MRD detection in landmark-positive patients who relapsed was 0.08% (range 0.002% to 2.41%, n=25, Extended figure 6b). 12 patients were landmark positive before adjuvant therapy (Supplementary Table 15, Figure 3a-d). A pre-adjuvant ctDNA positive patient (CRUK0086) had undetectable ctDNA following adjuvant radiotherapy and was disease-free until non-cancer associated death; the remaining 11/12 patients suffered eventual clinical relapse despite 5/11 patients exhibiting undetectable ctDNA following adjuvant therapy indicating that ctDNA clearance in this setting may not always predict a positive outcome (Extended figure 6c).

In 102/108 patients evaluable for survival analyses, landmark-positive patients exhibited a HR of 5.3 (95% CI 2.9 to 9.7, P=1e-09 log-rank test) for OS and an HR of 6.8 for FFR (95% CI 3.7 to 12.3, P=6e-13 log-rank test) relative to landmark negative patients (methods, Extended figure 6d-e).

16/81 (20%) landmark negative patients emerged to be ctDNA positive during ctDNA surveillance prior to, or at, clinical relapse; this occurred a median of 359 days postoperatively (range 120 to 929 days), after a median of 3 negative postoperative plasma samples (range 1 to 9) at a median clonal ctDNA level of 0.02% (range 0.003% to 6.67%) (Figure 3a-b,d, Extended figure 6b).

## ctDNA lead times

Overall median lead time encountered in the cohort was 119 days (0 to 1137 days, n=63, methods). Lead times were associated with landmark status (Kruskal-Wallis P = 0.006); landmark-positive patients had the longest lead times (median 228 days [0 to 1137 days], n=23) relative to landmark-negative patients (median 76 days, [0 to 980 days], n=24, P=0.010, Wilcoxon-test) and landmark unevaluable patients (median 56 days, [0 to 477 days], n=16, P=0.005, Wilcoxon-test, Extended figure 6f).

## Imaging and ctDNA

We assessed postoperative ctDNA detection in the context of standard-of-care extracranial CT, magnetic resonance imaging, or positron emission tomography imaging surveillance in the adjuvant setting (methods, Figure 3, Supplementary Table 16). In patients who eventually experienced relapse, we identified 44 surveillance scans from 23 patients that showed no new abnormalities compared to prior imaging; 22/23 patients had plasma sampling performed prior to these scan(s) (Figure 3c-e). 9/22 patients were ctDNA positive before the scan and 8/9 ctDNA positive patients suffered eventual recurrence at sites covered by the extracranial scans (CRUK0590 experienced intracranial recurrence, Extended figure 6g). Thus, in some cases, positive postoperative ctDNA status preceded new abnormalities on surveillance imaging. Postoperative ctDNA detection before equivocal abnormalities occurred in 23 patients, 20/23 suffered subsequent NSCLC recurrence (Figure 3, Extended figure 6g-h). Prior to surveillance imaging showing new equivocal lymphadenopathy, 14 patients were ctDNA-positive and 20 patients were ctDNA negative. 11/14 (79%) ctDNA-positive patients subsequently relapsed with lymph node involvement at the equivocal site versus 6/20 (30%) patients ctDNA-negative before the scan (Fisher's test P=0.013, Extended figure 6i). Establishing ctDNA status may facilitate definitive therapeutic intervention at equivocal radiological sites, supporting prior findings from a cohort predominantly consisting of locally-advanced NSCLC treated with chemo-radiation therapy^6^.

## ctDNA-based measurement of clonal architecture

**Extended Figure 7:**
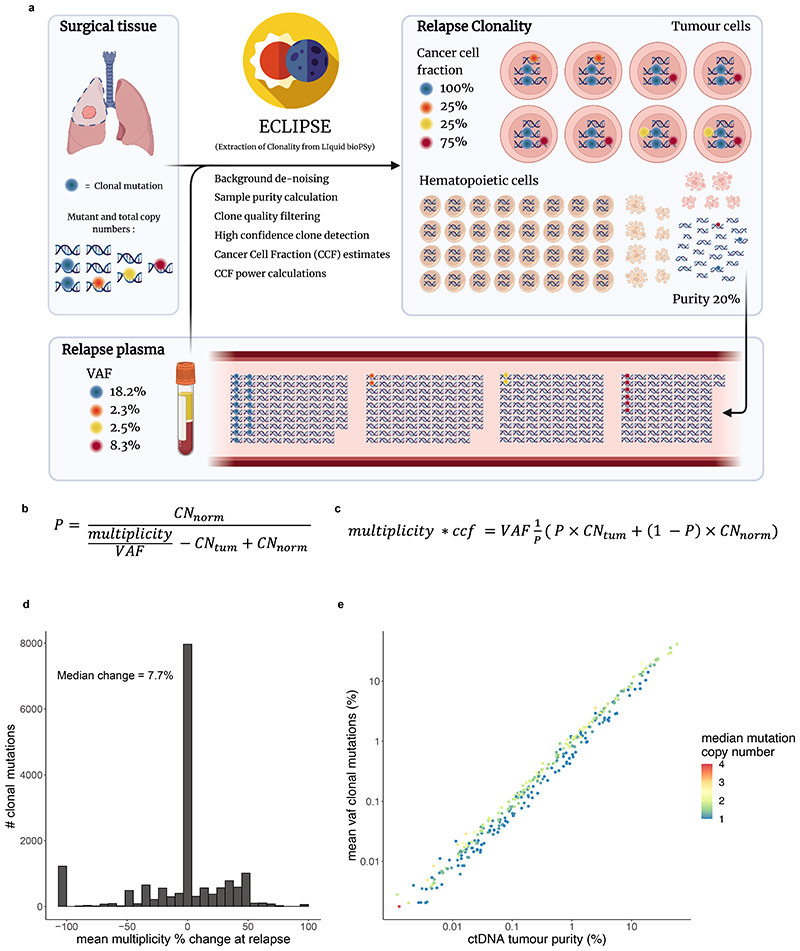
ECLIPSE methodology. **A**. A conceptual overview of the ECLIPSE method and data input types. CCF; cancer cell fraction and VAF; variant allele fraction. **B**. Equation to calculate tumour purity (the % of cells from which the DNA was derived which are tumour cells, see supplementary note 1, also termed 'cellularity' or 'aberrant cell fraction') using clonal mutations. **C**. Equation to calculate cancer cell fraction (CCF). Multiplicity = the number of mutated DNA copies in each mutated cell, CNt = total copy number in the tumour, CNn = total copy number in normal (non-tumour) cells, VAF = variant allele fraction, *P* = tumour purity (the % of cells from which the DNA was derived which are tumour cells, see Supplementary Note 1). **D**. Percentage change in mean multiplicity of clonal mutations comparing measurements in surgical excised tissue samples to tissue samples taken at relapse (46 patients with paired primary and recurrence tissue samples plotted). **E**. A comparison between mean clonal VAF of mutations and ctDNA tumour purity as calculated by ECLIPSE where data points (plasma samples) are coloured by the average copy number of tracked clonal mutations (measured using tissue sequencing). Multi-tumour patients and samples with evidence of copy number of instability at relapse are excluded. A total of 322 samples from 134 patients are plotted.

**Extended Figure 8:**
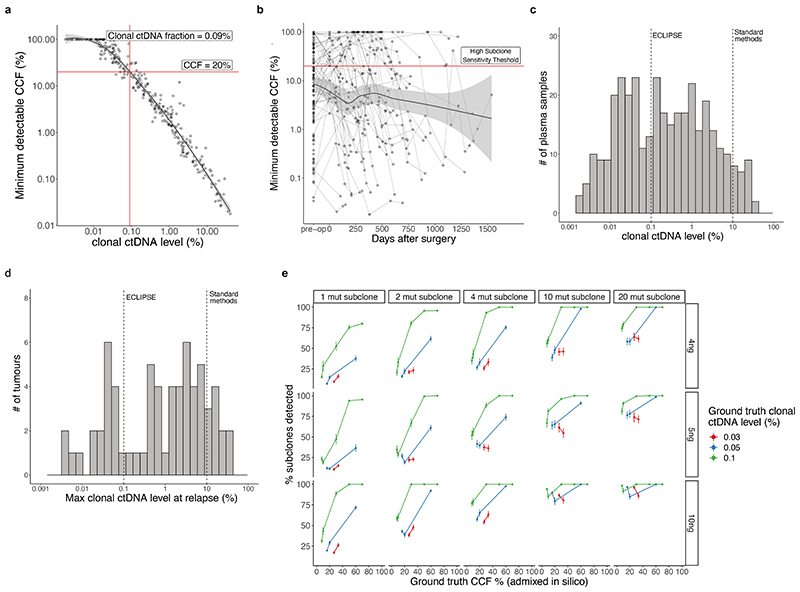
Subclone detection sensitivity of ECLIPSE. **A**. Minimally detectable CCF for each ctDNA positive sample compared to clonal ctDNA levels for each sample. All ctDNA positive samples included (N=354). Minimally detectable CCF was calculated using the minimum number of required reads for a positive (P<0.01) clone detection call (methods). **B**. Minimally detectable CCF over time for each patient with a horizontal line indicating the threshold for high subclone sensitivity samples (20% CCF). All ctDNA positive samples included (N=354). 61% of preoperative MRD positive samples were considered high subclone sensitivity and 66% of postoperative samples were considered of high subclone sensitivity (overall 64% of samples). **C**. A histogram of clonal ctDNA levels for all ctDNA positive samples (N=354) with vertical lines indicating thresholds for ECLIPSE evaluability and for traditional clonal deconvolution evaluability used for TRACERx tissue samples^27^ and previous clonal deconvolution approaches in ctDNA^13,76^. **D**. A histogram of maximum clonal ctDNA levels observed in post-operative samples for each patient with vertical lines indicating thresholds for ECLIPSE evaluability and for traditional clonal deconvolution evaluability (see **C**). This is shown for 66 patients who relapsed with ctDNA positive postoperative plasma. **E**. Validation of ECLIPSE detection rates across varying subclonal mutation number, clonal ctDNA level, subclone cancer cell fraction and DNA input amount into the assay. Subclones were constructed using ground truth *in vitro* spike-in experiments with 10-12 technical replicates for each input mass-allele fraction combination. These ground truth mutant allele fractions were then mixed *in silico* to construct 76,263 subclones varying across these parameters. Data from these experimentally derived subclones were then run through ECLIPSE and subclone detection rates across each of these parameters depicted.

To estimate tumour subclonal composition from deep targeted sequencing of plasma cfDNA we developed ECLIPSE. ECLIPSE leverages background noise estimates and tumour tissue derived copy number information to assess the presence or absence of specific tumour subclones and calculate their respective cancer cell fractions (CCFs) from low tumour content cfDNA data (Extended figure 7, methods, Supplementary Note). Plasma samples with clonal ctDNA levels of 0.1% (64% of ctDNA positive samples) had an estimated minimally detectable CCF of 20% for a representative subclone (methods, Supplementary Note, Extended figure 8a-d). Using 76,263 subclones constructed *in silico* from the AMP analytical validation spike in data, we estimated a detection sensitivity of 94% for 20% CCF subclones in 0.1% clonal ctDNA level plasma with 4 tracked mutations and 10ng DNA input (Extended figure 8e, Supplementary Note). We observed a decline in detection rates below 10ng DNA input, hence considered samples with ≥0.1% clonal ctDNA level and ≥10ng cfDNA input as 'high subclone sensitivity', and analysed their clonal composition with ECLIPSE.

**Extended Figure 9:**
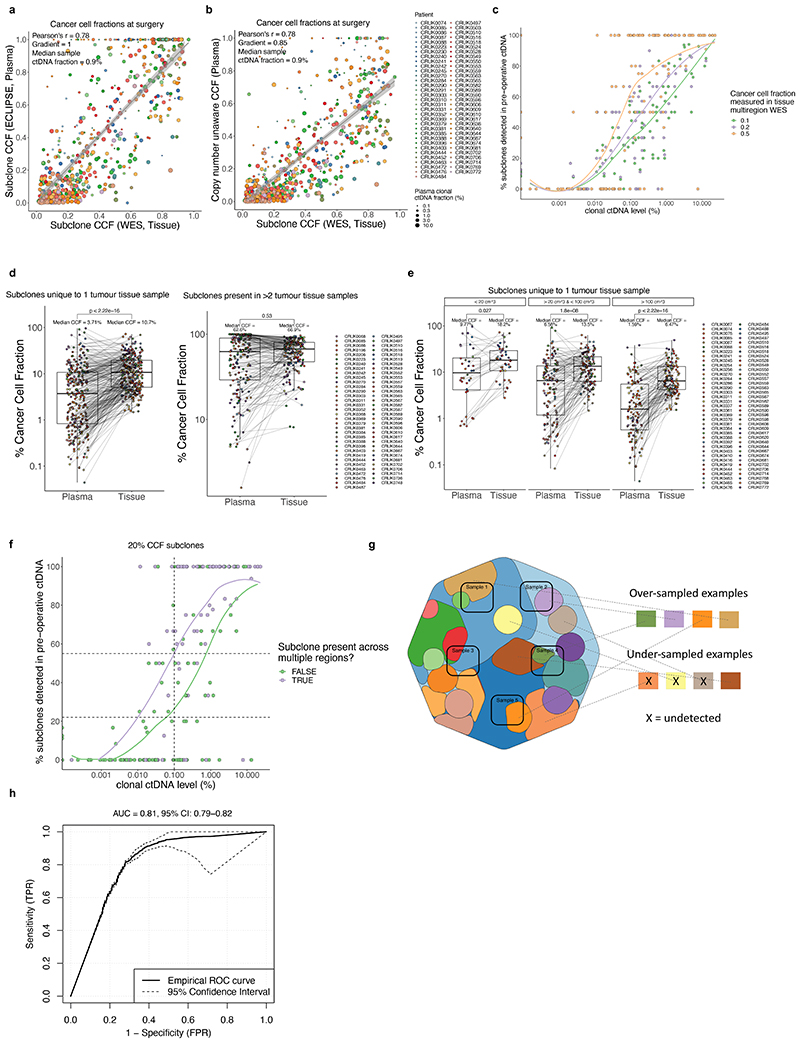
Time-matched comparisons between subclonal structure measured in plasma and in tissue at surgery. **A**. Correlation between cancer cell fractions (CCFs) as measured in preoperative plasma samples with phylogenetic data, >0.1% clonal ctDNA level & >=10ng DNA input (high subclone sensitivity samples) with ECLIPSE and those measured with multi-region tissue sequencing (M-seq) at surgery (N=71 patients and 684 subclones included). **B**. Copy number unaware CCFs calculated only using VAFs (methods) compared to tissue CCF from M-seq. All preoperative samples with phylogenetic data, >0.1% clonal ctDNA level & >=10ng DNA input (high subclone sensitivity samples) were included (N=71 patients and 684 subclones included). **C**. A scatter plot demonstrating the relationship between clonal ctDNA level and the proportion of multi-region tumour exome (M-seq) defined subclones detected by ECLIPSE based on varying subclonal cancer cell fractions as indicated, loess lines are fitted to the plots, n= 117 ctDNA positive preoperative samples. **D**. A comparison of pre-operative plasma CCFs and the average CCFs across all tissue regions sampled at surgery for clones that were unique to one tumour tissue region and for clones that were distributed across more than two tumour tissue regions. N=71 patients and 684 subclones included. A Wilcoxon-test was used to compare groups. **E**. A comparison of pre-operative plasma CCFs and the average CCFs across all tissue regions sampled at surgery for clones that were unique to one tumour tissue region separated between small (<20cm^3^), medium (>20cm^3^ & <100cm^3^), and large (>100cm^3^) tumours as measured on pre-operative PET/CT scans. N=71 patients and 684 subclones included. A Wilcoxon-test was used to compare groups. **F**. A comparison of detection rates in pre-operative plasma for 20% CCF subclones across a range of clonal ctDNA levels split by whether the subclones were spread across multiple primary tumour tissue regions or were limited to only a single primary tumour tissue region. 1924 subclones were assessed in 197 preoperative plasma samples. **G**. A map of tumour clones with areas of multi-regional tissue sampling indicated and clones which are over- and undersampled highlighted. Most of the undersampled clones are in fact not in the sampled areas creating a bias towards oversampling in clones which we are able to detect, an effect also called the 'winner's curse'. **H**. A ROC curve describing the sensitivity and specificity of detecting clonal illusion mutations using plasma-based CCFs with 95% confidence intervals generated using bootstrapping across 500-fold cross-validation (N= 71 tumours).

ECLIPSE measures of subclonal CCF from preoperative plasma samples were proportional to tumour exome multi-region sequencing measures of subclonal CCF sampled at surgery (Pearson R = 0.78, m (gradient) = 1, median clonal ctDNA level = 0.9 %, Extended figure 9a-b, Supplementary Note). Subclone detection rates in preoperative plasma increased with subclone size (CCF) in the primary tumour (Extended figure 9c). Using plasma-based CCFs, we found evidence of sampling bias in measurements of tissue CCF for subclones unique to a single tumour region (Extended figure 9d-g, Supplementary Note).

## Refining heterogeneity estimates using ctDNA

**Figure 4:**
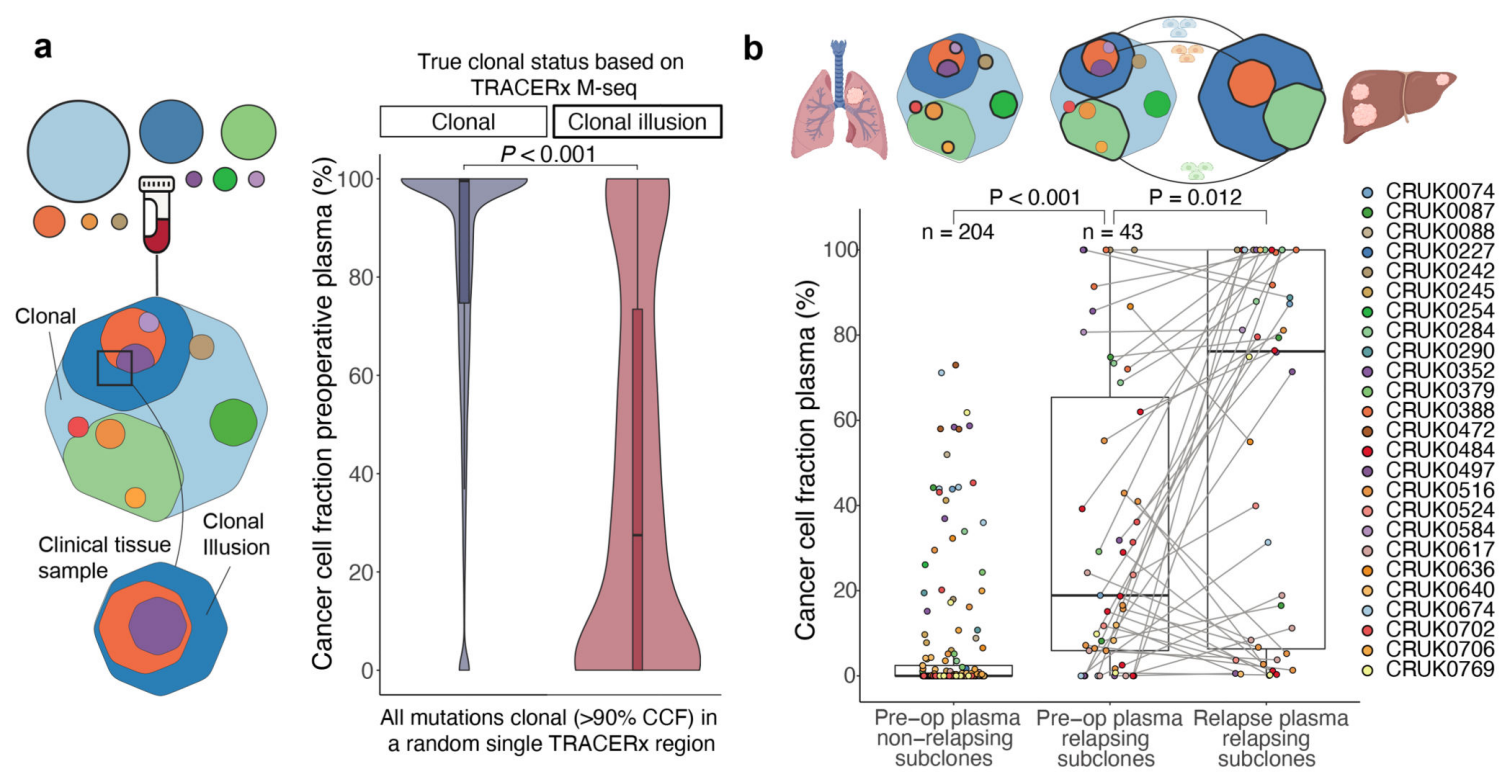
Clonality measurements in preoperative plasma overcome sampling bias from a single tissue sample and predict metastatic seeding potential. **A**. Depiction of a clonal illusion where a dark blue subclone is found in 100% of cells in a single clinical tissue sample. Such clonal illusion mutations may be detected in a clinical setting using ctDNA derived from many different tumour regions to increase accuracy of ITH measurements in the clinic. Mutations which were clonal (CCF > 90%) in a single, randomly selected tumour region are compared using plasma-based preoperative CCFs splitting by those truly clonal across all tumour regions in TRACERx (clonal) and those which, whilst they were clonal in the randomly selected region, were absent from other tumour regions (clonal illusion). Only data from a single randomly selected region was used by ECLIPSE to generate these CCFs. The distribution of plasma CCFs in each case is represented by a violin plot and a box and whisker plot. A Wilcoxon-test was used to compare groups. Only preoperative samples with at least 0.1% clonal ctDNA level (high subclone sensitivity samples, 71 samples from 71 patients) were included in this analysis (Supplementary Note for analysis of lower ctDNA levels). M-seq = Multiregional sequencing. **B**. Box and whisker plots of preoperative plasma primary tumour subclone CCFs split by whether a given subclone was found to be present or absent in cfDNA samples at relapse and postoperative plasma CCFs for relapse subclones at the last high subclone sensitivity timepoint. Only tumours with at least one sample >0.1% clonal ctDNA level (high subclone sensitivity) both preoperatively and postoperatively were included (N=26 tumours with CCFs from 247 subclones included). Two sided Wilcoxon-tests were used to compare groups.

In the TRACERx 421 cohort^27^a median of 12% of mutations were determined to be present in all cancer cells of at least one resected tumour region but were absent from other regions of the tumour, therefore exhibiting a clonal illusion (Figure 4a). ctDNA may be released from several regions of the tumour and resolve the true subclonal nature of mutations displaying a clonal illusion. In 71 TRACERx patients with high subclone sensitivity plasma samples available preoperatively, plasma-based CCFs were lower for clonal illusion mutations compared to mutations ubiquitous across all resected tumour regions (Wilcoxon-test, *P*<0.001, Figure 4a) and plasma CCFs could predict clonal illusion with an AUC of 0.81 (95% CIs: 0.79-0.82, Extended figure 9h). This suggests that collection of plasma alongside a single tumour biopsy can overcome tissue sampling bias, potentially increasing the accuracy of future heterogeneity-based clinical biomarkers^28,29^.

## Clonal expansions forecast metastasis

Predicting the subclonal nature of the subsequent metastatic recurrence at the time of surgery could inform precision adjuvant therapies against subclone(s) driving disease relapse. Primary tumour subclones (subclones detected in primary tumour tissue, excluding subclones unique to lymph-node or ipsilateral pulmonary metastases resected at initial surgery, methods) detected in postoperative cfDNA displayed larger CCFs in plasma samples taken prior to surgery relative to subclones not detectable postoperatively (Wilcoxon-test, P<0.001) and these metastatic subclones tended to expand further at relapse (Wilcoxon-test P=0.027, Figure 4b). This result indicates that primary tumour subclonal expansion measured non-invasively using ctDNA is associated with metastatic potential. In our companion manuscripts we demonstrate a similar effect using metastasis tissue sampling^30^ and describe increased proliferative transcriptional signatures associated with metastasis seeding primary tumour subclones^31^.

## Metastatic dissemination patterns in ctDNA

**Extended Figure 10:**
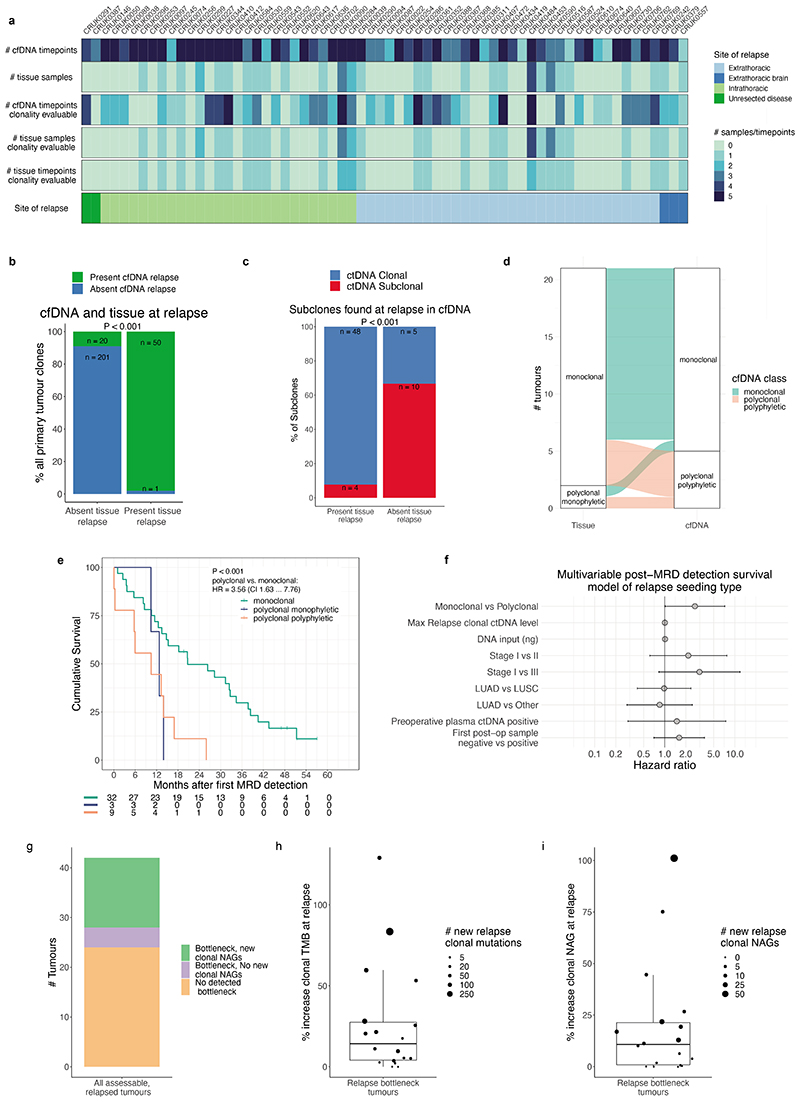
Clonal composition measurements in ctDNA after surgery. **A**. An overview of clonal structure evaluability at relapse for TRACERx patients in our cohort (N = 75 tumours) using either cell-free DNA and ECLIPSE or relapse tissue and WES/PyClone. **B**. ctDNA detection status post-operatively of subclones split by detection status in metastatic tissue. Untracked subclones (those without any mutations included in the PSP panels) were excluded (N = 26 tumours). P value indicates the result from Fisher's exact test. **C**. Clonal (estimated as present in 100% of tumour cells) vs subclonal (estimated as present in <100% of cells) status at relapse of primary tumour subclones by whether they were detected in cfDNA and metastatic tissue or cfDNA alone (N = 26 tumours). P value indicates the result from a Fisher's exact test. **D**. Metastatic dissemination class determined by tissue and by cfDNA in 22 cases with a metastatic biopsy, a postoperative high subclone sensitivity plasma sample, and a phylogenetic tree constructed. **E**. Overall survival Kaplan-Meier plot demonstrating time from the first MRD positive timepoint to death stratified by ECLIPSE metastatic dissemination class at relapse (monoclonal: light blue, polyclonal polyphyletic: purple, and polyclonal monophyletic: green). HR: Hazard ratio, CI: confidence interval. 44 patients were included in this analysis. The P value indicates the result of a log-rank test. **F**. A multivariable Cox proportional hazards model to predict overall survival from the time of first MRD detection including the clonality of metastatic dissemination at relapse, stage, maximum postoperative clonal ctDNA level, average DNA assay input, histology, and whether the first plasma sample after surgery was ctDNA positive, including only relapse patients. 44 patients were included in this analysis. Error bars indicate 95% confidence intervals. **G**. The frequency of high confidence subclonal to clonal bottlenecks (methods) at the latest possible plasma sample time point with sufficient clonal ctDNA level (high sensitivity subclone samples, N = 44 tumours) and which of these subclones harbour subclonal neoantigens (NAGs) which therefore become clonal at relapse. **H**. In cases of clonal bottlenecking at relapse, the percentage increase in the number of clonal mutations is shown as a box and whisker plot with the absolute number of new clonal mutations (N = 18 tumours). **I**. In cases of clonal bottlenecking at relapse, the percentage increase in the number of clonal NAGs is shown as a box and whisker plot with the absolute number of new clonal NAGs (N = 18 tumours). NAG = Neoantigen.

Comprehensive tissue sampling is challenging in the early-relapse setting. 44% of relapse patients had a tissue sample obtained at relapse, yet ease of plasma sampling allowed us to obtain high subclone sensitivity postoperative plasma samples in 61% of relapse patients (mean 2 samples per patient). 38% of relapse patients had high subclone sensitivity postoperative plasma samples, but lacked a relapse tissue sample (Extended figure 10a). In 26 patients with both high subclone sensitivity postoperative plasma and recurrence tissue, we found a high concordance between subclones detected in recurrence tissue and postoperative ctDNA (98% sensitivity [50/51 relapse tissue subclones detected that were tracked by PSPs], Extended figure 10b-c). Additional subclones detected in ctDNA but absent from relapse tissue were found in 6/26 patients (20 subclones). These subclones may have evaded tumour biopsy detection due to under-sampling of metastatic sites at relapse (Supplementary Note). This is consistent with our companion manuscript^30^ which suggests that a single metastatic biopsy is not sufficient to confidently capture all metastatic dissemination events.

**Figure 5:**
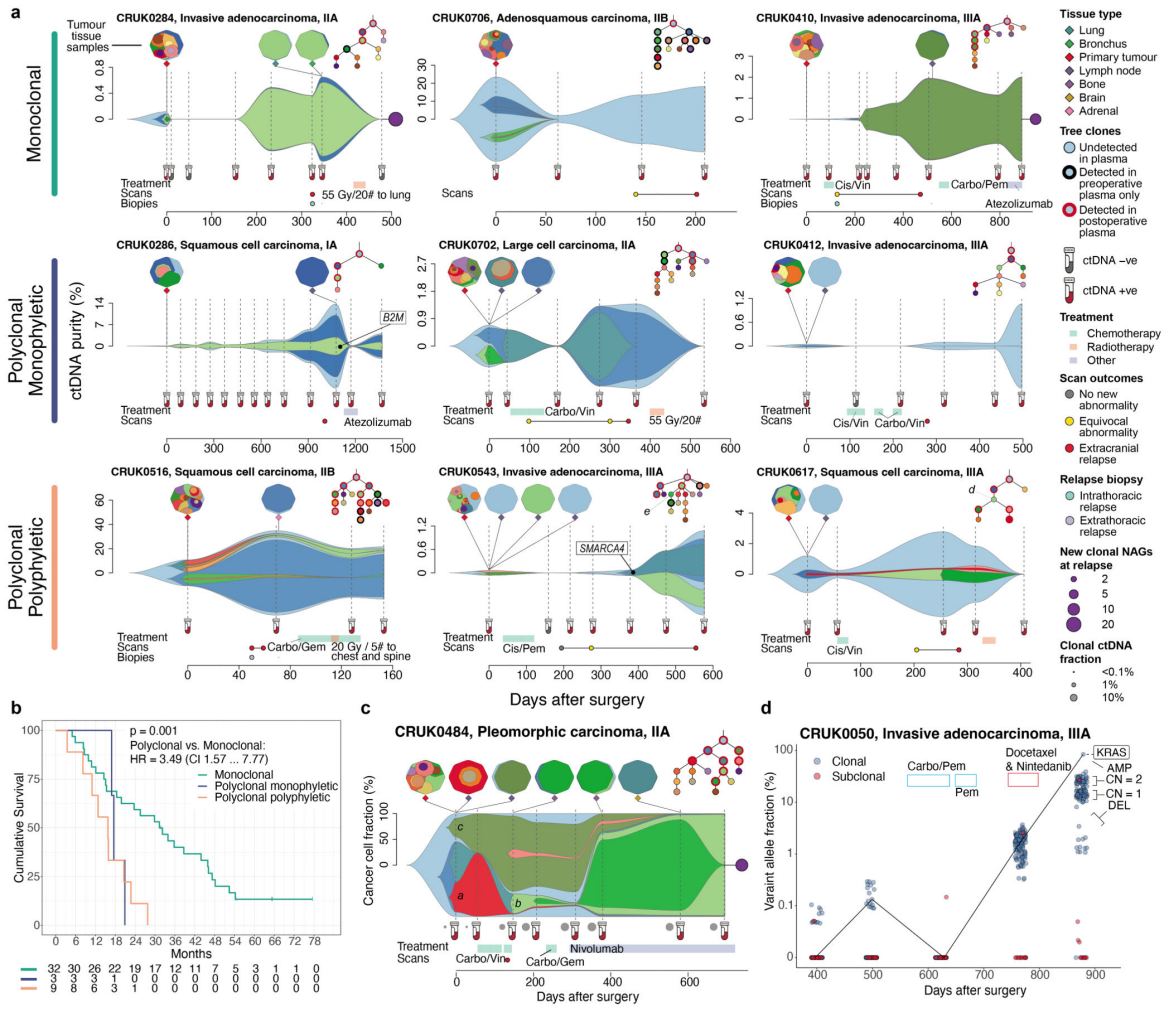
Longitudinal measurements of clonal evolution in plasma from surgery, through therapy and to recurrence. **A-D**. ctDNA purity for each clone is calculated by multiplying the clone CCF by the ctDNA purity of the plasma sample (methods) and represents the fraction of all cells from which cfDNA was derived which harbour a given tumour clone at each timepoint. Clonal nesting is based on the phylogenetic tree for each tumour. Data from all ctDNA positive plasma samples are shown including results from ECLIPSE of samples <0.1% clonal ctDNA level. Clone maps for each tumour tissue mass are depicted above the ctDNA based clonal structure with the phylogenetic tree. Metastatic dissemination class was defined using primary tumour subclones, excluding metastatic unique clones in surgically excised lymph nodes or intrapulmonary metastases (methods). Both CRUK0617 subclone *d* and CRUK0543 subclone *e* were not detected in ctDNA but their presence was inferred by detection of its daughter subclones (Supplementary Note). **A**. Depictions of longitudinal tumour evolution for examples of monoclonal, polyclonal monophyletic and polyclonal polyphyletic metastatic dissemination patterns. **B**. A Kaplan-Meier plot depicting differences in overall survival between metastatic dissemination classes (N= 44 tumours which had at least 1 high subclone sensitivity postoperative sample). A log-rank test was used to compare survival in the two groups. **C**. CCFs depicted through time and therapy for CRUK0484 who experienced a polyclonal polyphyletic relapse. **D**. Variant allele fractions for mutations tracked in CRUK0050 at recurrence. NAG = Neoantigen, Cis = Cisplatin, Vin = Vinorelbine, Carbo = Carboplatin, Pem = Pemetrexed, Gem = gemcitabine, Gy = Gray.

ECLIPSE-mediated calculation of subclone CCFs coupled with PSP targeting of the majority of sampled subclones in NSCLC resections (Extended figure 1b) facilitated estimation of dissemination patterns from the primary tumour to relapse using ctDNA (Supplementary Note). Tumours were categorised by the number of relapse-seeding primary tumour subclones (monoclonal = 1, polyclonal ≥ 1) and relapse-seeding primary tumour phylogenetic tree branches (monophyletic = 1, polyphyletic ≥ 1, Figure 1a, methods). Longitudinal plasma- and tissue-based clonal composition estimates from surgery to relapse are presented for 44 patients with high subclone sensitivity postoperative plasma (methods, Figure 5a, Supplementary Figure 1). We found an increased frequency of polyclonal metastatic dissemination at relapse when using ctDNA compared to recurrence biopsy tissue, driven by detection of ctDNA-unique subclones (10% polyclonal dissemination using tissue versus 24% polyclonal dissemination using ctDNA in matched cases, Extended figure 10d). Overall, 32/44 recurrent tumours were defined as monoclonal dissemination and 12/44 as polyclonal dissemination (3 polyclonal monophyletic and 9 polyclonal polyphyletic). Shorter OS from study registration and from the first ctDNA positive timepoint was observed in patients exhibiting polyclonal dissemination versus monoclonal dissemination (Figure 5b, post-registration OS: HR=3.49, 95% CIs=1.57 to 7.77, P=0.001 log-rank test, Extended figure 10e, N=44). OS from first postoperative ctDNA detection remained significant after adjustment for maximum postoperative clonal ctDNA level, assay DNA input amount, pTNM, preoperative ctDNA positivity, ctDNA detection in the first postoperative plasma sample and histology in multivariable analysis (Extended figure 10f).

## Longitudinal tracking of clonal evolution

We addressed whether phylogenetic tracking could detect changes in subclonal composition which may represent therapy-induced shifts in selection pressure. In 18/42 (43%) patients with a high subclone sensitivity postoperative plasma sample available, we estimated that subclones tracked from the surgically resected tumour had undergone a complete clonal sweep at recurrence, where a subpopulation of cells expands to become clonal across all tumour sites (methods, Extended figure 10g-i, Supplementary Note). We observed shifts in clonal composition in CRUK0484 concurrent with treatment (Figure 5c, Supplementary Note) including extinguishing of a subclone present in more than half of tumour cells after surgery (clone *a*) during adjuvant chemotherapy, and expansion of a minor subclonal lineage (clone *b*) during post-recurrence immunotherapy treatment which eventually outcompeted a parallel lineage (clone *c*). Despite three relapse tissue biopsies at different timepoints and metastatic sites, the dominant clone *c* was not detected in post-surgical tissue samples but only in a surgically excised lymph node. In CRUK0050 we observed a rapid increase in clonal ctDNA levels at day 876, following treatment of recurrent lung disease with cytotoxic chemotherapy (Figure 5d). A multi-modal distribution of clonal VAFs was observed in plasma, suggesting that 59/130 clonal mutations had altered their copy number state compared to samples taken at surgery (methods) including evidence for amplification of an oncogenic *KRAS* G12R mutation (84% VAF). This suggests the expansion of a new subclone during treatment harbouring significant chromosomal instability, not directly tracked by the PSP. **In summary**, we have demonstrated that preoperative ctDNA detection is prognostic in early-stage adenocarcinoma and implicated chromosomal instability as a predictor of ctDNA detection in this NSCLC-subtype. These findings suggest management of early-stage adenocarcinomas deemed high-risk based on preoperative ctDNA detection is inadequate, with innovation urgently needed. Postoperative ctDNA detection forecasted impending NSCLC relapse, agreeing with prior findings^5–8,32–34^. Here, 25% of patients were landmark MRD positive and 93% of these patients relapsed a median of 228 days post-ctDNA detection. Assessment of early treatment escalation in this high-risk population is required. ctDNA surveillance identified impending relapse in 20% of landmark negative patients, emergence of ctDNA during surveillance may reflect low-burden metastatic disease initially shedding ctDNA quantities below assay limit of detection (~95% sensitivity at 0.008% ctDNA level in ≥30ng DNA-input samples). Landmark MRD detection rates could increase with next-generation assays with improved ctDNA limits of detection^35,36,37^.

Prior publications have used high tumour fraction ctDNA samples (>10%) to calculate subclonal cancer cell fractions^11,13,12^. However, such samples are rare^16^, comprising only 9% of ctDNA positive samples from 14/145 (10%) ctDNA detected patients in this study. ECLIPSE, combined with AMP PSPs, enabled an estimated 94% detection sensitivity for 20% CCF subclones in plasma samples with 0.1% tumour content (64% of ctDNA positive samples) and could accurately estimate CCFs using such samples. We demonstrated that ctDNA can sample clonal structure from multiple different surgically excised tissue sites and capture additional heterogeneity at relapse when compared to analysis of relapse tissue samples. Despite this, two thirds of patients who suffered disease recurrence still harboured only one ctDNA-detectable metastasising primary tumour subclone (monoclonal dissemination). Low ctDNA levels and incomplete primary tumour sampling may however limit detection of additional disseminating primary subclones events. We observed a more aggressive disease course in patients with multiple metastatic dissemination events (polyclonal dissemination) suggesting that heterogeneity in the seeding population may provide fuel for Darwinian adaptation to different metastatic niches. However, the requirement to perform multiregional primary tumour sequencing currently limits the feasibility of determining metastatic dissemination patterns in the clinic.

ctDNA is poised to change (neo)adjuvant trial designs. Measurements of subclonal expansion in plasma before surgery may allow prediction of future metastatic subclones, offering the possibility for early intervention and suggesting new routes for biomarker development to target and eradicate such clones months or even years prior to relapse.

## Methods

### Patients and tissue samples

The TRACERx study (https://clinicaltrials.gov/ct2/show/NCT01888601) is a prospective observational cohort study that aims to transform our understanding of non-small cell lung cancer (NSCLC) the design of which has been approved by an independent research ethics committee (NRES Committee London, REC ref:13/LO/1546). Informed consent for entry into the TRACERx study was mandatory and obtained from every patient. All patients were assigned a study identity number that was known to the patient. These were subsequently converted to linked study identities such that the patients could not identify themselves in study publications. All human samples (tissue and blood) were linked to the study identity number and barcoded such that they were anonymized and tracked on a centralised database, which was overseen by the study sponsor only. The ctDNA cohort represents 188 TRACERx 421 cohort eligible patients and 9 additional patients (the following 9 patients were excluded from the final TRACERx T421 cohort [after ctDNA analyses were performed] and were analysed in this manuscript: CRUK0230, 0234, 0291, 0335, 0387, 0480, 0490, 0498, 0622). Reasons for exclusion from final T421 cohort are: CRUK0480, 0490: C>A artefact uncovered in exome data (excluded from ECLIPSE analyses), CRUK0291, 0234, 0230, 0387, 0622: Incomplete resection of NSCLC; CRUK0335: Concurrent oesophageal primary present at diagnosis; CRUK0498: 1 of 2 tumour regions contained lymphoid associated variants. Remaining preoperative plasma from 19 patients published in Abbosh et al. 2017^7^ was also analysed in this manuscript; these patients can be identified by CRUK IDs shared between manuscripts. Extended figure 3a describes the structure of the patient cohort analysed, patients analysed in the Extended figure 2 pilot cohort to assess optimum ctDNA detection thresholds were excluded from clinical analyses associating pre- and postoperative ctDNA detection with patient characteristics and survival outcomes (Figure 1 and 3) and biological analyses of ctDNA detection in lung adenocarcinoma (Figure 2). However, these patients were included in ECLIPSE clonality analyses (Figure 4 and 5). Multi-region tumour sampling was performed as previously described^2^. Relapse tissue samples, excess to diagnostic requirements, were also acquired. Sample extraction from tissue and whole blood followed the protocol in the TRACERx 100 cohort and exome sequencing was performed as previously described^2^.

### Analyses of adjuvant surveillance and relapse scan reports

Relapse site data was collected from anonymised standard of care imaging scan reports that occurred within 180 days of confirmed clinical relapse (Supplementary Table 14). Each report was reviewed by two clinicians and sites of disease documented. 2 patients lacked available scan reports (CRUK0311 and 0452); for these two patients data was gathered from TRACERx case report forms. Where an anatomical site was not covered by a recurrence scan this was marked as not evaluable. Anonymised surveillance (pre-relapse or relapse) scan reports were reviewed from 121/131 non-pilot patients who had donated longitudinal plasma samples (321 computed tomography scans, 7 Magnetic Resonance Imaging scans and 36 whole-body Positron Emission Tomography scans). Surveillance scan reports were not available in 10/131 non-pilot patients. These reports were categorised as showing no new abnormality compared to previous imaging, new equivocal abnormality (an equivocal abnormality was defined as any new change compared to a previous scan, equivocal changes were categorised as being related to new lung tissue abnormality including nodules, enlarging lymph-nodes, pleural abnormality or pleural effusion, lung atelectasis or collapse or other changes) or new unequivocal abnormality (scans showing a change that was viewed as definitive malignancy and resulted in a change in clinical management, Supplementary Table 16). This central review of reports was performed blinded to a patient's disease and death status. Where questions regarding interpreting the report arose, there was a dialogue with the cancer centre to establish an agreed assessment.

### Plasma samples

Blood samples were collected and processed to plasma as previously described^7^. Up to 4 ml of plasma per case was evaluated for the study (range 0.5 to 4 ml, median 4 ml, see Supplementary Table 2). For 1074 of 1095 samples circulating cell-free DNA was purified from plasma using the MagMAX™ Cell-Free DNA Isolation Kit in conjunction with the KingFisher™ Flex Purification System (ThermoFisher Scientific). KingFisher™ 24-deepwell processing plates were prepared according to the manufacturer's instructions (plate setup option for KingFisher™ Flex Magnetic Particle Processor 24DW, 4 mL of plasma, 75 uL elution volume). Automated cfDNA isolation was performed on the KingFisher™ Flex. For the remaining 21 samples, cfDNA was extracted as previously described^7^. Eluted cfDNA samples were quantified on the Qubit 3.0 Fluorometer using the Qubit dsDNA HS Assay Kit (ThermoFisher Scientific) according to the manufacturer's instructions. The single nucleotide polymorphism (SNP) profile of cfDNA from a patient was matched back to normal exome data and samples exhibiting discordant SNP profiles were excluded as sample swaps (n=26/1095 plasma samples analysed).

### Volumetric analyses

Tumour volume was determined on the basis of pretreatment (PET-) CT scans using 3D Slicer. Contours of the primary tumour were manually segmented on each axial CT slice. Window settings were adjusted if necessary to exclude vessels, lymph nodes or adjacent mediastinal tissue. If no accurate delineation of the primary tumour was possible (e.g. large cavity, pleural effusion or atelectasis), the patient was excluded from volume analysis (Extended figure 4a); patients with minor cavities within tumours were included. These steps were performed by a trained resident and all contours were confirmed and edited where necessary, by an experienced radiologist. Relevant clinical demographics including gender and tumour location were cross checked with imaging appearances for each scan analysed. Volumetric data is in Supplementary Table 8.

### Library preparation using Anchored-multiplex PCR

Anchored-Multiplex PCR (AMP) is a nested multiplex – PCR enrichment chemistry that incorporates strand specific priming and the incorporation of unique molecular identifiers (UMIs) into sequenced reads^15^. Cell-free DNA, fragmented peripheral blood mononuclear cell (PBMC) DNA (60ng) or fragmented normal tissue DNA (60ng) was end-repaired phosphorylated and A-tailed. An adapter containing a universal priming site, the indexes for multiplexing and a UMI is then ligated onto DNA. One round of target specific PCR was performed with a gene-specific primer 1 (GSP1) which amplifies against the P5 primer in the adapter, and a further round of PCR was then performed with a second nested gene-specific primer (GSP2) and a primer that incorporates a second primer containing a P7 index. Strand-specific priming was performed in both rounds of amplification facilitating the identification of positive and negative strand input DNA molecules during informatic analyses.

For cfDNA libraries, indexed libraries were quantified on either the ViiA 7 Real-Time PCR System or QuantStudio Dx Real-Time PCR Instrument (ThermoFisher Scientific) using the KAPA Library Quantification Kit (Roche). Libraries were individually normalised on the Fluent 1080 Automated Workstation (Tecan), then symmetrically pooled and adjusted to a final concentration of 2 nM or 1.25 nM for standard or Xp NovaSeq loading workflows, respectively. Library pools were prepared and sequenced on the NovaSeq 6000 System (Illumina) according to the manufacturer's protocol. We aimed to sequence each library to ~10 million reads. The on-target deduplication ratio of the library, which describes the ratio of raw on-target reads to unique molecular identifier [UMI] supported on-target reads (UMI supported reads contained 5 or more supporting raw reads with a matched molecular index) was then evaluated. In samples where initial sequencing depth resulted in on target de-duplication ratio less than 10:1, additional sequencing was performed; this quality control step was introduced to maximise recovery of UMI-families (which require at least 5 UMI-supported reads) from high complexity samples to ensure recoverable information from these samples, thereby reducing bias (given that only UMI-families are considered in our analyses). This QC step resulted in the majority of cfDNA libraries (1052/1069) having median de-duplication ratios more than 5 (Extended figure 1f). PBMC and normal tissue libraries were either sequenced on the NovaSeq 6000 system (Illumina) or the NextSeq system (Illumina).

### MRD Calling Algorithm

We generated an MRD caller (v0.1) that investigated background sequencing noise on an intra-library basis (Supplementary Note, Figure 1a). The MRD caller utilised the Archer informatic pipeline to clean input reads and generate deduplicated UMI supported reads. The cleaned, deduplicated, and error corrected UMI-supported reads were aligned to hg19 and used to evaluate alternate observations at predefined positions where tumour-specific variants were present in the patient's tumour (tumour-informed positions). Only “deep” consensus reads supported by 5 or more PCR duplicates (UMI-corrected) were used to infer expected sequencing noise as well as calculate signal for the MRD calling algorithm.

Alternate bases at tumour-informed positions were subject to a strict set of quality filters consisting of an off target filter, a read strand bias filter, a sequencing strand bias filter, background error rate filter, and variant allele frequency outlier filter to remove artefactual signals. The variant allele frequency outlier filter functioned by performing PAM (partitioning around medoids) clustering of the variant allele frequencies (VAFs) of the tumour-informed positions that passed previously described filters. K was set to 2 in the clustering algorithm, thus yielding a high VAF group and a low VAF group. If one of the the two clusters had significantly higher VAFs (as indicated by non-overlapping confidence intervals of the highest VAF of the low VAF cluster and the lowest VAF of the higher VAF cluster) and contained 3 or fewer tumour-specific variants, those variants were removed from consideration downstream in the algorithm.

Next, intra-library background error-rates (ERs) were calculated. ERs were used to establish the level of noise present in each library that had to be confidently exceeded to allow an MRD call to be made. To calculate background library ERs, the number of UMI-supported alternate observations (DAOs, deep alternate observations) were tallied across the assay's region of interest (ROI) for each trinucleotide context (TNC) and for each possible alternate position based on the plus strand of the reference sequence. The ER corresponding to each TNC alternate was calculated as DAO/DDP (DDP, deep UMI-corrected depth across a TNC alternate). In order to measure only PCR and sequencing error, a position in the ROI was not included in the TNC ER calculation if the VAF at that position for a particular alternate was > 1% (on the basis that this could represent a clonal haematopoiesis associated mutation or a single nucleotide polymorphism).

A mapping of tumour observed variants and their accompanied TNC ERs was generated. Any tumour observed variant with a corresponding TNC ER upper confidence interval that was above 0.01% was filtered from the MRD calling algorithm. PAM clustering was used to generate 4 “D-groups” of TNC error-rates from qualified TNCs. The population weighted average TNC error-rate was calculated for each of the four D-groups based on the product of the TNC error-rates included in each D-group cluster and the total DDP for each TNC. The generation of 4 D-groups ensured that there was sufficient intra-library DDP coverage of each D-group to make precise estimations regarding ERs for variants within each group.

To determine whether ctDNA was present in the sample, the total observed DAOs summed across tumour specific positions remaining after filters were compared to the number of DAOs that were expected due to background ERs as dictated by the D-groups. A one-tailed exact Poisson test was applied where the total remaining observed DAOs served as the value being tested and the expected number of DAOs due to error served as the lambda of the Poisson distribution. If the resulting P value of the test was below a pre-specified alpha threshold set to 0.01 then the sample was classified as MRD positive. The Supplementary Note contains details regarding how the pre-specified alpha threshold of 0.01 used in these analyses was generated.

To investigate whether a single mutation targeted by a panel was present we utilised the specific trinucleotide error-rate corresponding to the mutation of interest and a one-tail Poisson test to assess if the number of DAOs across the mutation of interest was above expected background ER. If the number of DAOs was higher than expected background error using an alpha threshold of 0.01 then a variant was deemed confidently detected. Supplementary Tables 13 and 17 contain sample and variant level outputs of the MRD caller pipeline.

### Estimating the effect of panel size on minimal detectable allele fraction

We estimated the minimally detectable allele fraction (MDAF) for total ctDNA to estimate our ctDNA sensitivity in each TRACERx plasma sample. We estimated the number of observed consensus mutant reads that would be required to produce a ctDNA positive call using at a threshold of P < 0.01, given the total background noise estimated across all mutations considered. To assess the effect of the number of mutations tracked on our ctDNA sensitivity, we randomly subsampled 1, 2, 5, 10, 20, 50, 75, 100 and 150 mutations for each of our 200 mutation panels and assessed the minimal detectable allele fraction. The median MDAF for samples with 20ng to 30ng using 50 mutations (0.008%) was very similar to the sensitivity estimated using our *in vitro* validation data (>90% sensitivity at 0.01% allele fraction).

### Data inputs for ECLIPSE

For each mutation ECLIPSE requires mutation identifiers (chromosome, position, reference allele, alternative allele), a sample identifier, the number of supporting reads, sequencing depth, estimated background error rate, clone identifier, a binary call for whether the mutation is clonal or subclonal, mutation multiplicity, total copy number at the mutated locus in tumour cells, total copy number at the mutated locus in non-tumour cells (default = 2). ECLIPSE also takes several optional inputs, including variants to be filtered for clone and tumour presence calls due to high background error, variants that should be filtered from all analysis for a specific sample and a measurement for the maximally expected normalised standard deviation of CCF in high confidence clones used to identify clones with incoherent CCF distributions which may represent mutation clusters that are not true clones. The background error rate is the probability, for any given read, to observe the specified mutation due to sequencing error. For application of ECLIPSE to our TRACERx data we estimate this using trinucleotide context specific error rates at non-mutated loci in the deep targeted sequencing data (see MRD Calling Algorithm section). The clone identifier, clonal vs subclonal status, mutation multiplicity and total copy number in tumour cells can be calculated using standard copy number extraction and clonal deconvolution methods (ASCAT^38^, Battenberg^39^, Pyclone^40^, DpCLust^39^) used for high tumour purity (>10%) samples, for example from tissue samples, which can then be used as estimates for these variables at the time of ctDNA sampling. Clonal status can be more accurately and comprehensively extracted from the sequencing of multiple high purity samples from the same patient, as is performed in TRACERx, but is not essential. See Application of ECLIPSE to the TRACERx cfDNA data section for further details.

### Stepwise description of ECLIPSE

#### 1 VAF denoising

Variant allele frequencies (VAFs) are denoised by subtracting the estimated background error, provided to ECLIPSE for each variant. For a description of estimating background error in this dataset see MRD calling algorithm section. Variants in each clone are grouped into clusters (via k-means clustering) with similar background error profiles, where the number of clustered groups is determined by the sum of the error estimated across all variants, so that if equally dividing the total error from all variants of a clone, each group would have a combined error of at least one mutant read. Therefore, if a clone has a total combined error of less than two mutant reads only one group will be used. A maximum number of clusters is set to four as the default value (which was used for application to the TRACERx plasma sequencing data). The average background error of each group per variant is subtracted from the number of supporting reads for all variants in each group and divided by the sequencing depth to calculate denoised VAFs.

#### 2 ctDNA tumour purity calculation

Denoised VAFs are used with mutation multiplicities, total copy number at the mutated locus and clonal vs subclonal mutation status for each mutation provided to ECLIPSE to calculate an estimate of ctDNA tumour purity using the equation shown in Extended figure 7b for each clonal mutation. The equation shown in Extended figure 7c is a rearrangement of that shown in Extended figure 7b for clonal mutations where CCF = 1. We summarise the mean of these values to provide a final estimate of ctDNA tumour purity per sample.

#### 3 CCF calculation per mutation and subclone

For all mutations, the sample's ctDNA tumour purity, denoised VAF, multiplicity and total copy number at the mutated locus are used in the equation shown in Extended figure 7c to calculate an estimate of CCF for each mutation in a given plasma sample. The clone identities for each mutation are provided to ECLIPSE and should be calculated independently using standard methods, which leverage SNP coverage applicable to high purity samples^38–40^. The mean per-mutation CCF is used as a CCF estimate for each clone. Any CCF estimates > 1, presumed to represent noise, are limited to 1.

#### 4 Poor quality clone identification

Mutation clustering using standard methodologies is imperfect and will be fitted to the samples of higher purity used for cluster identification (usually matched tissue samples), excluding lower purity samples which ECLIPSE is able to analyse using deep targeted sequencing. Erroneous clusters may not continue to track at similar CCFs in data from new samples. To identify such clusters, the distribution of ECLIPSE-calculated CCFs in each clone in a ctDNA sample are quantified using normalised standard deviations (SDs). The SDs can then be compared to the expected CCF distributions of high confidence clones, for example clonal clusters in higher purity plasma samples. In our data we quantified the normalised SD of all clonal clusters in samples of greater than 5% purity and took the upper 95% confidence interval for this data calculated at 0.56. Subclonal clusters with normalised SDs for CCFs > 0.56 were considered of poor quality and were not considered for analysis. This identified 2.6% of clones in the TRACERx data as of poor quality. Expected CCF distributions will be highly dependent on the input data for ECLIPSE and should therefore be benchmarked on each data set. A function in the ECLIPSE R package is provided to calculate an upper 95% CI of normalised SDs for CCFs in clonal clusters in high purity samples, as was performed for this dataset.

#### 5 Clone present calling

To determine whether each clone is present or absent from each sample (see High specificity subclone detection section), the sum of expected background error is compared with the sum of the observed signal across all variants in the subclone with a one-sided Poisson test. Mutations with high error that should be excluded from these calculations can be specified.

#### 6 Tumour present calling

To determine whether any tumour cells are present in each sample, the summed expected background error is compared with the summed observed signal across all variants tracked in the sample with a one-sided Poisson test. Mutations with high noise that should be excluded from these calculations can be specified.

#### 7 Minimal detectable CCF estimation for each subclone

Determination of the CCF equivalent to the minimal number of supporting reads across all variants in a subclone that would be required for a significant clone to be called as present (Poisson test, P <0.01, see High specificity subclone detection section).

#### 8 Minimal detectable CCF estimation for an average subclone for each sample

Determination of the CCF equivalent to the minimal number of supporting reads across all variants in a representative subclone that would be required for a significant clone to be called as present (Poisson test, P <0.01, see High specificity subclone detection section). The background is taken as an average of the background error in all subclonal mutations tracked in a given sample and is representative for a subclone tracked by four mutations as default, the average number tracked in this dataset. This value allows comparisons of minimally detectable CCF limits across samples.

#### 9 Minimal detectable purity estimation for each sample

Determination of the purity equivalent to the minimal number of supporting reads across all tracked variants that would be required for a significant tumour to be called as present (Poisson test, P <0.01).

#### 10 Testing for the absence of a complete clonal sweep for each subclone

A subclone which is detected in high purity samples used for mutation clustering may expand through a full clonal sweep later in the disease course. We would therefore expect to observe CCFs of 100%, indistinguishable from CCFs of clonal mutations after such an event. For each subclone in each sample, a Wilcoxon-test is performed to compare the CCFs of each subclone to the CCFs of clonal mutations in the same sample. The resulting P value indicates whether there is significant evidence that the subclone is significantly below 100% CCF and therefore is only present in the minority of tumour cells, without a full clonal sweep.

### Minimal detectable CCF estimates for each subclone

To quantify our limits of detection of CCF in each sample and subclone, ECLIPSE calculates the number of supporting reads for all mutations in each subclone that would be required for a positive clone detected call (P<0.01 threshold) based on the number of expected background error reads using the qpois function in R (stats package, v4.1.2). This value is then divided by the mean depth of all variants in a subclone to simulate a representative minimal detectable VAF for mutations in a given subclone and these values are input into the equation shown in Extended figure 7c to calculate the equivalent CCF, using an average of the mutation multiplicity and total copy number across all mutations in the given subclone and the ctDNA purity of the sample (see Determination of ‘tumour purity’ in plasma section, supplementary note). These minimally detectable CCF thresholds are highly dependant on the number of variants tracked in each subclone, hence to provide a single representative and comparable value for each plasma sample we also simulated the minimal detectable CCF for a subclone containing four mutations, which is the median number of mutations tracked in each subclone in this study but can be altered as an argument to ECLIPSE. The minimal detectable number of supporting reads in these four mutations was estimated using the average background error profile of all subclonal mutations in a given sample.

### High specificity subclone detection

A similar approach to that for high specificity MRD detection in ctDNA was undertaken for detection of subclones in this study, by estimating the background sequencing error in a trinucleotide context specific manner leveraging non-mutated positions in the target regions of the sequencing library (see MRD calling section). These background error estimates were then provided to ECLIPSE. These background noise rates were multiplied by depth to calculate the expected number of background reads alternate at each mutated position. These expected background read counts were then summed for all variants in a clone and used as the background lambda for a Poisson test comparing the sum of the observed number of reads across the same mutations. A P value threshold of 0.01 was chosen to call a clone present to match the threshold determined for MRD calling with *in vitro* spike in experiments and the pilot cohort of patients comparing post-surgery samples to relapse status.

### Application of ECLIPSE to the TRACERx cfDNA data

Inputs to ECLIPSE were prepared from the TRACERx 421 cfDNA and exome sequencing data as follows for all analyses unless otherwise specified. For inputs extracted from matched tissue exome sequencing data, all available samples were used, including from relapse tissue where possible. Clonal vs subclonal status, cluster identities and multiplicity status were extracted using presence and absence informed clustering as previously described^30^ which builds upon the PyClone algorithm^40^. Total copy number in each tumour sample at each mutated locus was extracted as previously described^30^. Normal copy number was presumed to be diploid. For metrics calculated per sample, purity-adjusted averages (which were computed as the sum of the metric per sample, multiplied by the sample purity and divided by sum of all sample purities) were calculated across the whole tumour for input into ECLIPSE for multiplicity and total tumour copy number. The number of variant supporting reads and depth in each cfDNA sample were calculated considering only unique reads with at least 5 supporting duplicates to minimise background error. Trinucleotide specific error estimates were used as input to the background error per variant. “Hard filtered” variants (those excluded from all ECLIPSE analyses) were those with “failed filters” of “primer_abundance_filter”, “primer_strand_bias”, “sequence_strand_bias”, “dro_cutoff” and “dao_imbalance”. Additionally “mid filtered” variants were those with “failed filters” “tnc_error_rate” where the background error was considered to high for inclusion in estimates of MRD (see MRD Calling Algorithm section) and were also excluded for estimates for clone presence or absence in ECLIPSE (see steps of ECLIPSE section).

### Validation of ECLIPSE CCFs vs tissue exome M-seq CCFs

To compare ECLIPSE estimated CCFs to those estimated using validated methods applied to tissue sequencing data at a matched time point, we compared purity adjusted averages (see Application of ECLIPSE to the TRACERx cfDNA data section) of CCFs from surgically excised tumour tissue for each subclonal cluster^30^, a benchmarked variant of PyClone^40^ to subclonal CCFs estimated in ECLIPSE (Extended figure 9a). The analysis was performed on high subclone sensitivity preoperative samples, which are defined as those with at least 0.1% clonal ctDNA level. These were samples with an estimated minimally detectable CCF of at least 20% (see power analysis in Extended figure 8a) compromising 61% of MRD positive preoperative samples from 67 patients. While a formal method for CCF estimation in deep targeted sequencing data has not been previously published for comparison, we compared ECLIPSE to a VAF only method for CCF estimation. In this method, which is naive to copy number status, the mean VAF of each subclonal cluster is divided by the mean VAF of the clonal cluster in each sample (Extended figure 9b). This caused a consistent underestimation of CCF relative to estimates from tissue exome sequencing, driven by the higher average multiplicity of clonal mutations compared to subclonal mutations, which more commonly occur before large scale copy number amplifications (for example whole genome doubling) which increases mutation multiplicities of mutations that have already been accrued.

### Validation of subclone detection rates using our data and ECLIPSE

To further investigate the sensitivity of subclone detection at different frequencies using ECLIPSE, we analysed data generated using in vitro spike-in experiments described in Extended figure 2. To generate these data, different mutation allele fractions were spiked into wildtype DNA and different total DNA amounts inputted into our AMP PCR NGS assay, including 12 replicates for each spike-in mutation fraction and input amount combination. In total this comprised 398 spike-in samples, each with 50 spiked in mutations, which were then subject to our AMP PCR NGS pipeline, identical to that applied to our plasma-derived cell free DNA samples. We subsampled mutations from each of these spike-in experiments *in silico* to represent subclones with 1, 2, 4, 10 and 20 mutations (a median of 4 mutations were tracked per subclone in our TRACERx ctDNA panels). Each of these 'subclones' was combined *in silico* with data from spike-in mutations at higher mutant allele fractions to represent clonal mutations. This allowed us to construct *in silico* subclones with various cancer cell fractions (determined by the ratio of spiked in mutant allele fraction of the subclonal mutations to the spiked in mutant allele fraction of the clonal mutations), across various clonal ctDNA levels (the spiked in mutant allele fraction of the clonal mutations) across a range of total DNA inputs to the assay. Although these data derive from mixing mutations together from different experiments *in silico*, the concentrations of DNA are known from ground truth, hence these mixtures provide a deeper level of validation, controlling for various sources of noise in the assay and providing technical replicates. In total we constructed 76,263 subclones from these data which varied in CCF, clonal ctDNA level, number of mutations per subclone, and assay DNA input amount. We ran these data through ECLIPSE using background noise estimates from the same libraries to determine how the rate of subclone detection varies with these four parameters. We focused on the lower DNA inputs (<= 10ng) as the greatest variety of allele fractions were spiked in for these inputs, enabling construction of a wider range of CCFs, and these samples represented the most challenging scenarios for subclone detection. We calculated the fraction of subclones detected for each experimental replicate at each specified clonal ctDNA level and at each CCF. We then used the resulting distribution of detection rates across experimental replicates, for each clonal ctDNA level and CCF, to calculate 95% confidence intervals.

### Clonal illusion analyses

For analysis of clonal illusion, we reran ECLIPSE for each TRACERx patient considering only data from a single randomly selected tumour sample to simulate a clinical biopsy, including multiplicity and total copy number estimates. Clonal status of each mutation was recalculated using a 90% CCF threshold in the selected region and only mutation specific, rather than clone specific, estimates of CCF were analysed, which removed the requirement cluster identification. To analyse clonal illusion, all mutations which would be considered clonal in the randomly selected region were split by their clonal status when considering all TRACERx regions. Mutations were therefore either truly clonal in all regions (labelled clonal) or were in fact subclonal when other tumour regions were considered and therefore harboured clonal illusion in the randomly selected region. ECLIPSE estimates (using only data from the randomly selected region as described) were then displayed for these two mutation groups in Figure 4a. To determine sensitivity and specificity using ROC analysis of clonal illusion detection, all apparently clonal mutations (>90% CCF) in the randomly selected region were used with the ROCIT R package (v2.1.1) with scores inputted as the mutation specific single region ECLIPSE CCF estimates and final classes considered as the Clonal or Clonal Illusion status leveraging all tumour regions in TRACERx.

### Longitudinal depictions of clonal evolution in cfDNA and tissue

Representations of clonal evolution over time were depicted using the ECLIPSE plasma CCFs per subclone, the subclonal CCFs in matched tissue samples extracted either at surgery and the phylogenetic subclone relationships calculated from tissue multi-regional exome sequencing as described^30^. ECLIPSE plasma subclone dynamics were plotted using modified code from the fishPlot R package (v0.5)^41^ and clonal structure of tissue samples were plotted using an R package developed in-house called cloneMap (version 1.0)^42^ distributed on GitHub (https://github.com/amf71/cloneMap). Only clones with at least one cfDNA tracked mutation which was not hard filtered (see Application of ECLIPSE to the TRACERx cfDNA data section) in all samples were shown in the ctDNA and tissue clonality representations and the phylogenetic trees. Clonal dynamics in cfDNA were represented by ctDNA purity for each clone which was calculated by multiplying the CCF of each clones by the ctDNA tumour purity of each cfDNA sample, therefore presenting the proportion of cfDNA derived cells (including normal hematopoietic cells) which belong to a specific subclone. 44 patients which relapsed from their disease excised at initial surgery and where phylogenetic trees were available from tissue exome sequencing were depicted in Figure 5 and Supplementary figure 1. The CCF of a parent clone was maximally limited to the sum of the CCFs of its daughter subclones. In Figure 5c, CCFs, rather than ctDNA purities, are plotted for each clone, as the purity/ctDNA fraction in this patient varied over several orders of magnitude. Use of ctDNA purities for each clone which would make it difficult to distinguish clonal composition changes in low purity/ctDNA fraction samples on a linear scale, required for intuitive interpretation of such area plots. Sample purities are depicted in this case as grey circles below the CCFs.

### Definition and detection of clonal sweeps at relapse

Subclones undergoing a clonal sweep were those which expanded after surgery, when they were first detected in tissue WES, increasing to 100% CCF, i.e. such previously subclonal mutations were now estimated to be present in every tumour cell and parallel subclonal lineages were estimated to have been extinguished. To call instances of a clonal sweep ECLIPSE performs a Wilcoxon-test comparing the CCF of all mutations in a given subclone to the clonal mutation in each sample. The resulting P value indicates the probability that the subclone has undergone a clonal sweep with a null hypothesis of a clonal sweep being present. We considered a clonal sweep present when this P value was greater than 0.05 and absolute mean subclone CCF was at least 90%. For each patient the latest possible time point with high subclone sensitivity (i.e. a clonal ctDNA level of at least 0.1%) was used to determine clonal sweeps at relapse. To estimate how these clonal sweeps at relapse modified the tumour trunk, we added all mutations and neoantigen in relapse clonal sweep subclones (including those clustered together in exome sequencing but not tracked in cfDNA) to the clonal mutations for re-estimation of clonal tumour mutational burden and clonal neoantigen burden at relapse. All subclones tracked by PSPs, including those which may have been specific to surgically excised lymph nodes or ipsilateral intrapulmonary metastases were included in this analysis.

### Determination of phylogenetic metastatic dissemination class at relapse

Phylogenetic metastatic dissemination classes at relapse were determined separately using either relapse tissue or post-operative cfDNA for each relapse patient in this study, where relapse tissue and/or a high subclone sensitivity postoperative sample (>0.1% clonal ctDNA level) was available. Our companion article^30^ has focused on metastatic disseminations estimated from primary tumour tissue including disseminations detected at surgery to local lymph nodes (also excised at initial surgery). Metastatic dissemination to excised local lymph nodes cannot be estimated in cfDNA alone, as preoperative ctDNA may derive from either metastatic lymph nodes or the primary tumour. For tissue based metastatic dissemination calls at relapse, relapse seeding primary tumour subclones from Al-Bakir et al. that were tracked by PSPs were considered^30^. These clones were used to determine whether a single clone or multiple primary tumour clones seeded the tissue relapse (monoclonal and polyclonal dissemination respectively). Using the phylogenetic tree in polyclonal cases, we also determined whether clones were directly descended from one-another in the same clade (polyclonal monophyletic) or if there is branching between the disseminating clones into different clades (polyclonal polyphyletic). For metastatic dissemination calls at relapse based on post-operative cfDNA, the number of relapse seeding clones was determined *de novo* without reference to the relapse tissue samples. If all primary tumour subclones detected in postoperative ctDNA were direct descendants in the phylogenetic tree and were present at 100% CCF, the relapse was considered monoclonal. If any primary tumour subclone was present at significantly less than 100% (using a Wilcoxon-test comparing clonal cluster CCFs to each CCFs in each subclonal cluster, *P* < 0.05 and also requiring a mean subclone CCF < 90%) then the metastatic dissemination at relapse was considered polyclonal. In polyclonal cases, if the subclones present at relapse were direct descendants of one-another the metastatic dissemination at relapse was considered as polyclonal monophyletic and if they were branched into separate clades they were considered as polyclonal polyphyletic metastatic disseminations at relapse. Metastasis unique subclones tracked by PSPs in surgically excised lymph nodes or intrapulmonary metastases which were also present at relapse were not considered when defining primary tumour to relapse metastatic dissemination patterns, as they represent metastasis to metastasis seeding rather than primary to metastasis seeding. For example CRUK0620 is determined to have a monoclonal metastatic dissemination pattern at relapse, despite having multiple subclones and branches present in post-operative ctDNA, as only one of those subclones (subclone *d* on the phylogenetic tree) is present in the primary tumour and others ctDNA relapse clones were only detected within surgically excised metastases (an ipsilateral intrapulmonary metastasis and several lymph nodes). This definition of metastatic dissemination as primary to metastasis dissemination, rather than surgically excised tumour to recurrence dissemination is consistent with our companion manuscript and the literature^43^. We did not find a significant difference between the number of tracked mutations in post-operative plasma subclones which were detected compared to those which were undetected(Wilcoxon-test *P*=0.13, median number of variants tracked = 4 in both cases) suggesting power of detection did not strongly influence which clones were detected in relapse cfDNA.

### Quantifying chromosomal instability in CRUK0050

At the last plasma sample time point in CRUK0050 a multimodal distribution of clonal mutation VAFs was observed (Figure 5d) where each mode likely represented a set of mutations with a similar average multiplicity across the tumour. To assign each clonal mutation to a VAF cluster the mclustBIC and then Mclust functions from the mclust package (v5.4.7) were used. In this case 4 VAF clusters were identified. The mutations in the lowest VAF cluster had an average VAF 1.2% and mutations in the second lowest VAF cluster had an average VAF of 12.1%. If the lowest cluster represented mutations with multiplicity of 1, the large majority of mutations in the remaining 3 clusters would therefore be presented at very high multiplicities (>10) given their >10 fold higher average VAF which would represent a biologically implausible amount of allele duplication across the genome, equivalent to 5 compounded whole genome doubling events. A more plausible explanation of these data is that the lowest cluster represents mutations with a multiplicity of 0 in a new subclonal population which has expanded at the final time point to a CCF >80%. Consistent with this, the mutations in the lowest VAF cluster were present at very similar VAFs in the previous plasma sample time point, consistent with the notion that these mutations remained only in those same tumour cells at the final time point, but not in the expanded subpopulation. The second lowest VAF cluster also contained 100% of the mutations which were associated with a multiplicity of 1 in the tumour tissue WES data. Therefore we assigned the second lowest VAF cluster a multiplicity of 1, the second highest cluster (average VAF of 25%) a multiplicity of 2 and the highest VAF mutation (KRAS G12 variant, VAF = 84%) a multiplicity >2. These mutation multiplicities were compared to the integer multiplicity estimates in surgically excised tissue WES to determine which mutations had undergone a change in copy number, which was the case for 59/130 clonal mutations.

### Designing AMP-MRD enrichment panels

tumour-informed personalised AMP-MRD enrichment panels were designed for 197 TRACERx patients. A median of 50 variants per panel (range 0 to 50) were chosen using the ArcherDx panel design algorithm (v0.1) and a median of 150 variants (range 34 to 153) were chosen using variants selected from TRACERx multi-region exome sequencing data derived from early-stage NSCLC resections (including primary tumor, lymph-node metastases or ipsilateral intra-pulmonary metastasis if applicable). Due to alterations in our TRACERx exome sequencing pipeline between panel design (2019 to 2020) and final analysis, a small fraction of mutations (3%) targeted by patient specific panels were no longer called with high confidence in tissue exome sequencing data. These mutations were included in MRD analyses (to align with the originally intended analysis approach plus prevent any possible bias conferred by manually removing these variants from consideration by the MRD caller) but were excluded from analyses of clonal structure. For Archer variant selection WES sequencing data from the highest purity tumour region and from the paired germline DNA were used. The algorithm then identified those variants for which there was high confidence that the variants were not artefacts and were tumour specific using the following criteria: the quality of the primers targeting the variant (to ensure high sequencing coverage of the target variant), predicted error rate for the variant in error corrected bins and known involvement in cancer. The predicted error rates for each variant was based on an analysis of AMP cfDNA libraries sequenced on a NovaSeq instrument.

The algorithm then determines which variants can be targeted using an ArcherDX AMP panel and from this set of variants the 50 most informative mutations are targeted based on these criteria: the quality of the primers targeting the variant (to ensure high sequencing coverage of the target variant), predicted error rate for the variant in error corrected bins and mappability. The predicted error rate for each variant is based on an analysis of AMP cfDNA libraries sequenced on a NovaSeq instrument. This error rate analysis was performed by performing targeted variant calling on every possible SNV in a set of Archer LiquidPlex cfDNA libraries. The TRACERx variants were selected from variants called in surgically excised tumour samples using the TRACERx WES pipeline^2^ for ranking. SNVs were ranked based on their (1) driver designation, (2) trinucleotide context as described above, (3) mean mutation allele count. All SNVs were categorised as either “neoantigen”, “clonal” or “subclonal”. Up to 50 variants were picked from each category. Neoantigens were additionally ranked by binding affinity^44^. Subclonal mutations were picked to represent all phylogenetic mutation clusters, picking up an equal number of mutations from each cluster when possible, up to a total of 50 maximum. Finally, 50 clonal variants were picked. If the sum of subclonal and neoantigen mutations was less than 100, the difference was picked from the list of clonal mutations.

Each personalised enrichment panel also contained 90 primers targeting 45 common single nucleotide polymorphisms (SNPs). During analyses the zygosity of these SNPs in a cfDNA library is compared to their zygosity in the whole exome sequencing data for that patient to confirm that a sample swap did not occur. In addition the coverage provided by these primers helps in establishing the background PCR and sequencing error rate for a library. These 45 SNPs were chosen based on being present in each Gnomad subpopulation at a frequency of 25%-75% to maximise utility in detecting sample swaps.

ArcherDX variant choosing and panel design deviated from the standard workflow in two cases. In the case of the pilot sample CRUK0297 the tumour and non-tumour samples used in design were not properly matched and rare germline variants appeared to be tumour-specific as a result. The ArcherDX variant choices in this panel included many germline variants. For this reason the cfDNA libraries for CRUK0297 underwent manual blanking of the germline targeted variants to facilitate use of these samples in the pilot patient analyses. All subsequent ArcherDX panel designs included a quality control step to confirm that the common population polymorphisms in the tumour and non-tumour samples matched. The second case in which panel design deviated from the standard ArcherDX workflow occurred in the design of CRUK0296. The variant call data for a tumour-normal tissue pair could not be obtained in the standard format for this patient. In this case, the standard variant caller could not produce a result so the variant caller VarDict^45^ was used and data was not available for the non-tumour sample in the standard format. As a result two germline variants (chr6:31118898:A:T and chr16:70928307:C:A) were targeted. These two variants were removed from consideration in making the MRD call automatically by the Outlier Filter in every library prepared with this panel (refer to the Library-specific MRD Calling section above) but were kept in all analyses and not manually blanked. Two patients lacked Archer picked variants (CRUK0157, 0227), CRUK0157 as the exome data could not be processed by the Archer variant picking pipeline and CRUK0227 due to an error during PSP primer ordering.

### Neoantigen pipeline

HLAHD was used to determine the patient-specific HLA composition. 9-11mer peptides harbouring nonsynonymous mutations coupled with patient-specific HLA were used as input to NetMHCPan4.1. A Rnk_EL <0.5 was used to determine strong binder peptides.

### Analytical validation experiments

For experiment LOD1, 634 samples of fragmented DNA with a known SNP profile (Genome in a Bottle DNA, NA24385) were added to a background of four other fragmented Genome in a Bottle inputs (NA24149, NA24631, NA24694 and NA24695). Six AMP enrichment panels were generated targeting 50 SNPS heterozygous in NA24385 and absent from the other four cell lines. To generate contrived samples NA24385 DNA was spiked into a background of the other four samples at ratios of 0.006% to 0.2% by mass to target variant allele frequencies ranging from 0.003% to 0.1% allele frequency (since heterozygous variants are present at 50% in the neat NA24538). As part of the same dilution series, admixtures with target allele frequencies of 1%, 5% and 10% were made. These mixtures were used as input for AMP library preparation to confirm that mixing based on mass achieved the desired target allele levels. The spike-in variant level was measured in these higher AF libraries by adding the number of deep alternate reads across the targeted SNPs and dividing by the total coverage of all deep reads across targeted SNPs. This analysis confirmed that the spike-ins achieved the targeted AFs. Fragmented DNA inputs from 2ng to 80ng were used in the experiment to reflect the range of DNA inputs encountered in a clinical setting. Overall 559 of 634 samples were deemed evaluable for LOD1 analysis (62 samples failed because of incorrect DNA input used, determined by on-target read per primer per ng input of <30 or >400, 8 samples failed because they had less than 10 million reads and 5 samples due to potential duplicate libraries). Clinical samples were used in validation of AMP MRD (LOD2) and were prepared using a similar method to the Genome in a Bottle mixtures. Whole exome sequencing data from four patients was used to design patient-specific panels with the ArcherDx panel design algorithm containing 50 SNVs. The panels were used to prepare libraries using cfDNA from each patient and the overall tumour variant AF for each sample was calculated by adding the total number of deep unique reads containing a targeted tumour-specific variant and dividing by the sum of the deep unique coverage across all targeted tumour variants. All four patient cfDNA libraries had a total AF of >1%. A single mixture was made using cfDNA from healthy donors and used to dilute the patient cfDNA. These dilutions were performed as a serial dilution. First a dilution was made targeting a 1% total AF and libraries were prepared using this mixture. The total AF was measured for this sample and a dilution correction factor was calculated to account for differences in conversion efficiency between the background cfDNA. For example, if a 1% AF was targeted and an AF of 1.3% was observed then this would indicate that the patient cfDNA is more efficiently converted to library than the background and more background DNA would need to be used. Mixtures were then made to achieve AFs of 0.1%, 0.05%, 0.01%, 0.008% and 0.005%. A total of 100 libraries were prepared at 5 AFs and 3 input masses. 48 blank samples (DNA donated from 24 healthy donors) were analysed to assess assay specificity. Panel observed allele frequencies were calculated by taking the number of deep alternate reads noted across the AMP panel, removing estimated background error and dividing by deep depth across the panel. For experiment LOD3 an AMP PSP was generated targeting 300 heterozygous SNPs in Genome in a Bottle product HG002. HG002 was diluted into a background mixture of HG003, HG005, HG006 and HG007 at multiple dilution levels such that heterozygous variants located in HG002 were present at final AFs of 0%, 0.003%, 0.005%, 0.006%, 0.01%, 0.03% and 0.05% and 0.1%. Using stocks of these contrived input materials, 10 ng was input into library preparation. 2 libraries were prepared at AFs from 0% to 0.05% and a single library was prepared at 0.1% AF using the 300 variant AMP panel. *In silico* subsampling was performed on the 15 libraries. Nine *in silico* panels were generated for each library (3 targeting 200 variants, 3 targeting 100 variants, and 3 targeting 50 variants) and MRD caller results evaluated alongside the 300 variant PSP result (overall 150 results generated from the 15 libraries). For assay sensitivities at specific spike-in categories, Clopper-Pearson binomial two-sided 95% confidence intervals were calculated in Extended figure 2e-f using the R package DescTools(v0.99.44)^46^ and the function BinomCI.

### Simulation analysis to assess specificity

Trinucleotide context of tumour-specific SNVs within each TRACERx AMP-MRD pilot cohort panel was assessed. Based on these data mock tumour signatures (genomic positions covered by the enrichment primers with positions of similar expected error rates of the targeted SNVs) were generated. A mock variant was added to a mock signature if the following criteria was met: It is bi-directionally covered by primers intended for MRD detection, It contained the same TNC-group error rate as the true MRD variant it is replacing, it was not a known population SNP variant as dictated by Ensemble's Variant Effect Predictor version 94.5, had a error-corrected coverage delta no more than 2,000 compared with the true MRD variant, and was not used within any other mock tumour signature, including itself. Thus, the resulting mock signatures targeted bases that are not mutated in the primary tumour and any positive MRD call from these mock signatures was by default a false positive. 3157 mock signatures across 91 pilot cfDNA libraries were interrogated for MRD positive calls A simulated ctDNA level was estimated for each sample by taking the number of deep alternate reads noted across the mock signature, removing estimated background error and dividing by deep depth across the mock signature. Data from this simulation is present in supplementary table 18.

### Digital droplet PCR orthogonal validation

Digital droplet polymerase chain reaction (ddPCR) orthogonal analyses were performed in 30 preoperative plasma samples from TRACERx patients who also had preoperative plasma analysed by the AMP personalised tumour informed approach and 8 negative controls (preoperative plasma from patients diagnosed postoperatively with non-malignant disease). TRACERx patients were selected as having clonal driver mutations that could be targeted by a single ddPCR assay. Clonal driver mutations targeted included *KRAS G12R*, *G12D*, *G12V*, *G12S*, *G12A*, *G12C* and *EGFR L858R*. The ddPCR assays used were SAGAsafe® assays (SAGA diagnostics) and had been designed and developed on the BioRad QX200 Droplet Digital PCR system. ddPCR analyses were performed at SAGA, SAGA received plasma (median 4.8 mls, range 2.5 to 5.2mls). cfDNA was extracted using the QiaAMP MinElute ccfDNA Midi Kit (Qiagen). cfDNA was eluted in 40 ul of Buffer EB. The entirety of cfDNA material was input in each case and ddPCR analyses were run in 4 replicate reaction wells per sample. All 8 negative controls (each assay tested once, *KRAS G12A* tested twice exhibited no mutant droplets detectable in control cfDNA, Supplementary Table 4).

### Transcriptional data analyses

Gene level transcription analysis used edgeR (v3.36.0)^47^ and limma (v3.50.3)^48^. The analysis included 101 tumour regions sampled from 34 ctDNA positive patients and 62 tumour regions sampled from 28 biological ctDNA low-shedder patients. The analysis took into account 18876 protein coding genes based on the HGNC database, retrieved on 03/04/2022. Genes with insufficient expression levels (count < 30) were filtered out and effective library sizes were calculated using the TMM (trimmed mean of M values) method. Count data was then transformed to logCPM values (log2-counts per million). Prior to linear modelling, a weight per observation was calculated based on the association between mean and variance. In order to take into account the association between tumour regions within patients, a per-patient consensus correlation was computed. Based on the logCPM table, the within-patient correlations and the ctDNA detection status, a linear model was fitted. A contrast matrix comparing ctDNA positives and biological ctDNA low-shedders was constructed alongside with the associated coefficients and standard errors, and the empirical Bayes method (eBayes function from limma v3.50.3^48^) was used to calculate the moderated t-statistics of differential expression. The resulting gene-level, two-tailed P values were adjusted for multiple testing using the Benjamini-Hochberg (FDR) method. Genes were noted as significantly differentially enriched if their adjusted P value was below 0.05.

The set of significantly overexpressed genes per detection category (n = 876 for ctDNA positives, n = 883 for biological ctDNA low-shedders) was used to calculate Reactome pathway enrichment (ReactomePA v1.38.0)^49^. The resulting P values were FDR-corrected and an adjusted P value cutoff of 0.05 was employed.

Additionally, pathway enrichment with respect to the Hallmark Gene Sets from the msigDB database was investigated. Pathway enrichment analysis was carried out on logCPM data including 17815 protein-coding genes using Gene Set Variation Analysis (GSVA v1.42.0)^18^. Fold change of GSVA enrichment scores comparing 101 tumour regions from 34 ctDNA positives and 62 tumour regions from 28 biological ctDNA low-shedders was calculated using the estimated marginal means (rstatix v0.7.1)^50^ method, using a linear mixed-effects (lmerTest v3.1-3)^51^ model to take into account the patient-tumour region associations, treating detection status as fixed effect and patient ID as random effect. The resulting pathway-level P values were FDR-corrected for multiple testing.

### Mutation analyses

Driver mutations in 181 genes from 70 ctDNA positive and biological ctDNA low-shedder patients (39 ctDNA positives, 31 biological ctDNA low-shedders) were included in the analysis. Clonality was determined based as part of the TRACERx WES pipeline. If a patient carried multiple mutations in the same gene with differing clonality, the clonal state was kept. In the gene-level analysis, the top 14 frequently mutated genes were considered. Genes were assigned to pathways as described by Sanchez-Vega et. al^52^. A Fisher's exact test was conducted in a two-tailed manner to compare the number of ctDNA positives and ctDNA low-shedders carrying alterations in the frequently mutated genes. The resulting P values were corrected using the FDR method.

### Chromosomal instability analyses

Copy number data including allele-specific copy numbers and purity estimates were derived from the TRACERx WES pipeline and were available for 245 tumour and lymph node regions collected from 63 ctDNA positive and biological ctDNA low-shedder patients (166 regions from 35 ctDNA positives and 79 regions from 28 ctDNA negatives). Cytoband analysis was conducted using GISTIC 2.0^23^, which takes one sample per patient as input. To investigate genomic regions of recurrent gains and losses, we constructed the single sample copy number profile for each tumour by selecting the maximum (for gains) or minimum (for losses) ploidy-corrected total copy number per segment across the genome. The GISTIC score difference of 0.5 was used as a threshold for significance cutoff. Cytobands were overlapped with output from GISTIC2.0 to get a mean GISTIC score for each cytoband. FLOH and wGII were analysed on the region level (548 tumour and lymph node regions from 137 patients - 166 regions from 35 ctDNA positive adenocarcinomas, 79 regions from 28 biological low-shedder adenocarcinomas and 303 regions from 74 non-adenocarcinomas). Comparing the chromosomal instability metrics between ctDNA positive and biological ctDNA low-shedder adenocarcinomas and non-adenocarcinomas was performed using a linear mixed model, taking into account the within-sample associations. Pairwise comparisons were made using the estimated marginal means method and P values were FDR-adjusted. Tumour regions were considered whole genome doubled (WGD) if the fraction of the genome with major allele >= copy number 2 was >50% as per previous publications^53^. Tumours were considered to have WGD if any single region harboured a WGD event. WGD data was available for 63 lung adenocarcinoma patients (28 biological ctDNA low-shedders, 35 ctDNA positives).

### Purity analysis

Using 245 regions from 63 patients (166 regions from 35 ctDNA positives and 79 regions from 28 ctDNA negatives), we performed an estimated marginal means analysis incorporating a linear mixed model approach to account for the within-sample associations. The analysis compared ctDNA positives with biological ctDNA low-shedders.

### ORACLE analysis

ORACLE scores were calculated by using the method developed by Biswas et al^20^, including 196 tumour and lymph node regions from 77 ctDNA positive and ctDNA low-shedder patients (109 regions from 35 ctDNA positives, 87 regions from 42 ctDNA low-shedders). Pairwise comparisons between the ctDNA shedders and ctDNA low-shedders were made using the estimated marginal means method with a linear mixed-effects model to account for the within-patient associations between tumour regions.

### Volume adjustment

Biological low-shedder samples were excluded from the volume-adjusted analysis if their size fell in the lowest quartile size range (< 6042.544 mm3). Transcriptomic and GISTIC analyses were repeated using the volume-adjusted dataset as described above. Taking into account the significantly overexpressed genes and significant cytobands in both datasets, Venn-diagrams were constructed for comparison and Jaccard Similarity Index was calculated to assess the statistical significance of the overlap. The similarity coefficient calculations were performed using the jaccard R package (version 0.1.0)^54^, and the corresponding P values were computed using the exact method. Venn diagram visualisations were created using eulerr (v6.1.1)^55^ and ggplotify (v0.1.0)^56^.

### Clonal mutation ctDNA levels

Mutations that were defined as clonal, either by PyClone clustering as described in our companion manuscript^27^, or (in the absence of PyClone data) that were present in every primary tumour tissue region analysed (ITH state = 1), and that were unfiltered by the MRD caller, were used in clonal mutation ctDNA level estimations. For each mutation the MRD caller estimated trinucleotide error rate associated with that mutation and the coverage of that mutation was used to estimate the number of expected error controlled reads we would observe due to error. Clonal mutation ctDNA level was then summarised as the total number of error-corrected reads across selected mutations, minus the expected error across these positions (rounded down to the nearest whole integer) divided by total clonal deep coverage. If the clonal ctDNA level was <0% (where background error was higher than observed variant DNA signal), it was assigned 0%. In two ctDNA positive samples, clonal ctDNA levels were measured at 0% due to mutations driving ctDNA positive status not being assigned a clonal status by the TRACERx pipeline (CRUK0296, sample 144717 and CRUK0039, sample 117025).

### Identifying probable technical negative and low-shedding adenocarcinomas

We generated a linear regression model (using the stats R package, function lm) where log-10 transformed tumour volume and histology was used to predict log-10 transformed clonal ctDNA level in 96 ctDNA positive non-pilot NSCLCs analysed in this cohort. We used this model to predict clonal ctDNA levels in 47 evaluable adenocarcinomas negative for ctDNA. We tested the capability of this model to predict clonal mutation levels in 8 independent ctDNA positive adenocarcinomas with volume data available analysed in our prior work using a separate assay^7^. In this test set 6 of 8 (75%) adenocarcinomas evaluated had mean clonal mutation levels above the lower 95% confidence interval of the model estimation. We calculated minimal detectable clonal ctDNA level (MDCL) in the 47 ctDNA negative adenocarcinomas by taking the minimum deep alternate observations needed to make a call in patient cfDNA samples and subtracting the estimated deep alternate reads that would occur due to noise in the panel (rounded down to the nearest whole integer). The resulting number was the number of clonal deep alternate reads needed to make a ctDNA positive call (we conservatively assumed all real deep alternate reads will be clonal). We divided this number by the clonal deep depth across the panel to calculate the minimum clonal ctDNA level that must be exceeded to make a call and called this value MDCL. Using the above linear model, we classified cases as probable technical negatives if the lower 95% confidence interval for predicted clonal ctDNA level was below MDCL and as probable low-shedders if the lower 95% confidence interval for predicted clonal ctDNA level resulting was above MDCL.

### Survival analyses

Overall survival (OS, events were death from any cause, outcome pre-defined in TRACERx protocol, see Jamal-Hanjani et al., 2017^2^), freedom from recurrence (FFR, events were lung cancer recurrence, patients disease-free or experiencing second-primary or death were right censored at last follow-up) and post-relapse survival (time from recurrence to death from any cause) analyses were performed. 169/187 non-pilot cohort patients were evaluated for Figure 1 and Extended figure 4 survival analyses (5 patients were excluded as they died within 30 days of surgery - CRUK0115, 0196, 0312, 0487, 0681, 4 patients were excluded as they had confirmed unresected disease after surgery - CRUK0230, 0234, 0291, 0387 and 9 patients with synchronous primaries were excluded given the emphasis on associations with tumour histology). For Extended figure 6d-e patients with synchronous primaries were included in landmark survival analyses as tumour histology was not considered in survival analysis. R packages survival(v3.2-13)^57^, survivalAnalysis(v0.3.0)^58^ and survminer(v0.4.9)^59^ were used to generate hazard ratios, forest plots, 1- and 2-year survival data and cox regression models in the manuscript. Differences in overall survival between metastatic dissemination classes at relapse were analysed using cox proportional hazard models from either the date of study registration and from the date of MRD detection. A multivariable cox proportional hazard model including maximum relapse ctDNA level, which is known to co-correlate with tumour burden and power for subclone detection, was used to account for this confounder relative to overall survival from the date of study registration.

### Lead time analyses

Lead time was defined as time from first postoperative ctDNA detection to radiologically confirmed clinical relapse. For lead time calculations we analysed patients with NSCLC relapse and assigned patients without postoperative ctDNA detection or with initial detection following clinical relapse lead times of 0 days. We excluded incompletely resected patients (n=4), patients with no ctDNA sampling before clinical recurrence (n=3, CRUK0516, 0557 and 0640) and pilot-patients (n=5) from these analyses.

### Statistical data analysis

No statistical methods were used to predetermine sample size. Analysis was performed in the R statistical environment version 4.1.2^60^. For I/O operations and general data manipulations, the R packages tidyverse (v1.3.2)^61^, data.table (v1.14.6)^62^, readxl (v1.4.1)^63^, fst (0.9.8)^64^, and qusage (v2.28.0)^65–67^ were used. All statistical tests were two-sided unless otherwise stated. For assay performance analyses, positive predictive value was calculated as all true positive results divided by the sum of true positive and false positive results; negative predictive value was calculated as all true negative results divided by the sum of false negative plus true negative results; sensitivity was calculated as true positive results divided by the sum of true positive and false negative results; specificity as true negatives divided by the sum of true negatives and false positives. For generation of heatmaps the R package ComplexHeatmap(v2.11.1)^68^ was used. For general visualisation purposes, R packages ggplot2 (v3.3.5)^69^, ggpubr (v0.4)^70^, ggrepel (v0.9.2)^71^, ggbeeswarm (v.0.6.0)^72^, scales (v1.2.1.)^73^, ggforce (v0.4.1)^74^, and cowplot (v1.1.1)^75^ were used. For plotting paired data ggpubr(v0.4)^70^ was used.

## Extended Data

## Supplementary Material

Supplementary Data Guide

Supplementary Note

Supplementary Table 1

## Data Availability

ECLIPSE is available as an R package in the Zenodo repository *here*. Upon publication the ECLIPSE github repository, from which this Zenodo repository is derived, will be made public. Code used to produce the figures in this manuscript is available on request.

## Data access statement

The cfDNA sequencing files, RNA-sequencing data and multi-region tumour exome sequencing data (in each case from the TRACERx study), used or analysed during this study have been deposited at the European Genome-phenome Archive (EGA), which is hosted by The European Bioinformatics Institute (EBI) and the Centre for Genomic Regulation (CRG) under the accession codes (EGAS00001006494, EGAS00001006517, EGAS00001006494) and is under controlled access due to the nature of the data and commercial partnerships arrangements. Details on how to apply for access are available on the linked page.
