# Supplementary material for: Tracking early lung cancer metastatic dissemination in TRACERx using ctDNA
Source: Nature. Author manuscript; Available in PMC 2023 Jun 1. (PMC7614605; doi:10.1038/s41586-023-05776-4)
Supplement: Supplementary Data Guide [file EMS176197-supplement-Supplementary_Data_Guide.docx]

**Supplementary Data Guide:**

**Supplementary Table 1|Composition of patient specific panels used in this manuscript**

This table summarizes the composition of the patient specific panels used in the study, describing the data shown in Extended Figure 1. The number of tracked clonal and subclonal variants are described on a per-patient basis, additionally, the number of variants with unknown clonality are included.

**Supplementary Table 2|cfDNA inputs from patient samples**

This table summarises sample level details on plasma volumes, cell-free DNA (cfDNA) concentrations extracted from plasma and quantity of cfDNA input into the Anchored Multiplex - PCR (AMP) MRD assay.

**Supplementary Table 3|Analytical Validation data**

This table summarises data from the 707, 50-variant patient specific panel (PSP) analytical validation experiments (LOD1 and LOD2) described in Extended Figure 2 and the Supplementary Note.

**Supplementary Table 4|ddPCR orthogonal validation data**

This table summarises data from the 48 plasma samples that were analysed with digital droplet PCR against clonal driver mutations identified in TRACERx multi-region sequencing data, to confirm accuracy of AMP MRD assay calls. Results described in Extended Figure 2 and Supplementary Note.

**Supplementary Table 5| Extended analytical validation data (300 mutation panel analyses)**

This table summarises data from the 300-variant patient specific panel (PSP) analytical validation experiment (LOD3) presented in the Supplementary Note and Extended Figure 2.

**Supplementary Table 6| Association of mutations tracked with estimated minimal detectable allele frequency in TRACERx libraries**

This table demonstrates how the minimum detectable allele frequency across TRACERx libraries varies with cfDNA input into the AMP MRD assay and number of mutations tracked by a PSP.

**Supplementary Table 7|Baseline demographics table**

This table summarises demographic and survival information across the TRACERx 421 cohort for reference, the 197 patients with baseline plasma evaluated in this manuscript and the 108 landmark evaluable non-pilot patient cohort.

**Supplementary Table 8| Patient clinical data and tumor volumes**

This table contains patient level demographic, survival and tumor volume data across the 197 patient ctDNA cohort.

**Supplementary Table 9|Differential gene expression analysis comparing ctDNA positive adenocarcinomas to low-shedder adenocarcinomas.**

This table shows the results obtained from the gene-level differential expression analysis comparing 101 tumour regions from 34 ctDNA positive adenocarcinomas and 62 regions of 28 ctDNA low-sheddder adenocarcinomas, as displayed by Figure 2A. The analysis is performed using limma-voom, see methods.

**Supplementary Table 10| Reads from significantly overexpressed genes in ctDNA positive adenocarcinoma to low-shedder comparison**

This table shows the gene counts of the 1,759 genes found to be significantly overexpressed in ctDNA positives or ctDNA low-shedders in the differential gene expression analysis.

**Supplementary Table 11|Reactome pathway enrichment analysis comparing ctDNA positive adenocarcinomas to low-shedder adenocarcinomas**

This table summarizes the results obtained from the Reactome pathway enrichment analysis (see methods), as shown in Figure 2B-C. Pathways are marked according to whether they are significantly enriched in ctDNA positives or ctDNA low-shedders.

**Supplementary Table 12|Gistic gene analysis output**

This table contains the genes found in significantly enriched regions identified by GISTIC (see methods). The significantly amplified or deleted cytobands shown in Figure 2H (ctDNA low-shedders) and 2I (ctDNA positives) are displayed.

**Supplementary Table 13|Sample level cfDNA MRD caller output**

This table contains cfDNA sample level MRD caller summary outputs (e.g., MRD Caller P values, depth achieved across a panel) for all plasma samples analysed in the manuscript. Details regarding plasma samples and exome SNP matching to exclude sample swaps are also included in this table.

**Supplementary Table 14|Anatomical sites of relapse**

This table provides detail regarding the anatomical sites of metastasis identified in patients within 180 days of clinical relapse.

**Supplementary Table 15|Adjuvant and post-recurrence treatment data**

This table provides detail regarding start and end dates of adjuvant and post-recurrence treatment data (if applicable) for patients who underwent longitudinal ctDNA monitoring in the manuscript.

**Supplementary Table 16|Scan data for the longitudinal MRD cohort**

This table provides clinical detail regarding surveillance imaging (pre-relapse imaging) that was performed for patients who underwent longitudinal ctDNA monitoring in the manuscript.

**Supplementary Table 17|Cell-free DNA MRD pipeline variant level output**

This table contains cfDNA mutation level MRD caller summary outputs (e.g., variant level MRD Caller P values, sequencing depth achieved across a mutation, MRD caller error filters activated by a mutation and trinucleotide error rates associated with a mutation) for all mutations targeted by PSPs across all plasma samples analysed in the manuscript.

**Supplementary Table 18|Specificity simulation in pilot cohort**

This table contains the results of 3157 *in-silico* simulations performed using pilot patient cfDNA libraries to estimate specificity of the AMP MRD assay during MRD caller p value threshold selection outlined in the Supplementary Note and Extended Figure 2.

**Supplementary Table 19|Sample level normal tissue MRD caller outputs**

This table contains normal tissue (e.g., peripheral blood mononuclear cells, normal lung tissue and genome in a bottle) sample level MRD caller summary outputs for a subset of patients analysed in Extended Figure 5.

**Supplementary Table 20|Variant level normal tissue MRD caller output**

This table contains normal tissue mutation level MRD caller summary outputs (e.g., variant level MRD Caller P values, sequencing depth achieved across a mutation, MRD caller error filters activated by a mutation and trinucleotide error rates associated with a mutation) for all mutations targeted by PSPs across the normal tissue samples analysed in the manuscript.

**Supplementary Table 21| Sample identifiers used for upload of raw sequencing data to the European Genome-Phenome Archive (EGA)**

This table contains sample and experiment identifiers which were uploaded to the EGA repository to associate with the raw sequencing data.

**Supplementary Figure 1|** **Longitudinal subclonal analyses across all relapsing patients with available phylogenetic trees and at least one postoperative time point with high subclone sensitivity, N = 44 patients).** ctDNA purity (CCF of each clone multiplied by ctDNA purity for each sample) is depicted for each detected clone at each time point as well as depiction of the clonal structure of matched tissue samples and the timepoints of their collections. Data from all ctDNA positive plasma samples are shown including results from ECLIPSE of samples <0.1% clonal ctDNA level. Treatment, scan outcomes, and biopsies received by each patient are also depicted. *In CRUK0620, this indicated tumour mass was in fact an intrapulmonary metastasis excised at surgery from the primary tumour depicted in the clone map to its right. While several subclones are present at metastasis in the ctDNA at relapse, most of these were only found in the surgically excised metastatic tissue and therefore represent metastases to metastases seeding rather than primary tumour to relapse metastatic dissemination. Only a single subclone (subclone f) in fact seeded the relapse from the primary tumour, hence this case is associated with monoclonal metastatic dissemination at relapse.
