# Supplementary material for: Tracking early lung cancer metastatic dissemination in TRACERx using ctDNA
Source: Nature. Author manuscript; Available in PMC 2023 Jun 1. (PMC7614605; doi:10.1038/s41586-023-05776-4)
Supplement: Supplementary Note [file EMS176197-supplement-Supplementary_Note.docx]

Supplementary Note for:

**Tracking lung cancer metastatic dissemination in TRACERx using ctDNA**

Contents:

**P2:** ctDNA detection approach and algorithm summary

**P2:** MRD calling threshold determination

**P3**: Analytical Validation of AMP ctDNA detection

**P4**: Orthogonal validation of AMP ctDNA detection

**P4**: Validation to assess targeting an increased mutation number

**P4**: Subsampling analyses of TRACERx libraries to assess the effect of tracked mutation number on sensitivity

**P5**: Preoperative ctDNA detection in synchronous primary cases

**P5**: Volume sensitivity analysis

**P5**: CRUK0498 false positive analysis

**P5**: ECLIPSE motivation & overview

**P6**: Determination of ‘tumour purity’ in plasma samples and contrast with ‘ctDNA level’

**P7**: Stability of mutant copy number alterations over time

**P8**: Minimal detectable CCF estimates for each subclone and sample evaluability thresholds

**P8:** In vitro validation of subclone detection sensitivity using spike-in experiments

**P9:** Undetected phylogenetic clones with detected daughter subclones

**P9**: Validation of ECLIPSE CCF estimates in pre-operative plasma

**P9**: Concordance between subclones detected in relapse cfDNA and relapse tumour tissue

**P9:** Evidence for detection bias causing CCF overestimation for small subclones in tumour tissue sampling

**P10**: Identification of complete clonal sweeps at recurrence

**P11**: Example of treatment-associated clonal evolution

**ctDNA detection approach and algorithm summary|** We utilised Anchored Multiplex PCR (AMP) chemistry to generate new phylogenetic tracking technologies. AMP incorporates Unique Molecular Identifier (UMI) indices to facilitate *in-silico* generation of single-stranded consensus reads. Only single-stranded consensus reads were analysed to reduce background sequencing error. Distribution of UMI-corrected sequencing depth and association of this parameter with cell-free DNA (cfDNA) input are summarised in Extended figure 1d-e. To create a consensus read (UMI-family) at least 5 reads supported with a matched molecular index were required (deduplication ratio of at least 5, methods). Lower deduplication ratios were observed in higher cfDNA input samples, yet only 17/1069 (1.6%) analysed plasma samples exhibited median deduplication ratios less than the 5 required to create a UMI-family (Extended Figure 1f). This demonstrates that sufficient complexity was extracted in most libraries to enact UMI error-control .

Figure 1a demonstrates the ctDNA detection approach leveraged in the study. Multi-region tumour exome data was used to generate a phylogenetic representation of a patient’s primary tumour containing clusters of mutations that are clonal (mutations present in all cancer cells) and clusters that are subclonal (mutations present in only a subset of cells). Bi-directional primers were synthesised to amplify genomic regions containing mutations identified through the multi-region tumour sequencing data, with the aim to target mutations from all clusters. UMIs were incorporated into amplified DNA and only consensus reads used to detect ctDNA. The position of interest where a mutation is expected was considered for ctDNA calling if there was no evidence of strand bias (that is variant bases are noted on both positive and reverse reads), no evidence of sequencing bias (variant bases are noted across both read 1 and read 2 during sequencing) and both forward and reverse primers were functioning (methods). Alongside evaluating the position of interest, the caller evaluated background sequencing positions from the targeted variants and background sequencing positions from primers targeting 45 germline SNP (single nucleotide polymorphism) primers. Any base errors observed in background reads were summed based on their trinucleotide context to generate trinucleotide error-rates (cohort wide error-rates summarised in Extended Figure 1g-h) . Trinucleotide contexts with an upper confidence interval less than 0.01% (error rate confidently below 0.01%) were used for ctDNA calling. Passed variants were subsequently evaluated by an outlier filter. If there were 3 or less outlier variants these were removed from the ctDNA calling algorithm (methods). For calling the presence or absence of ctDNA (panel-wide caller) all positions targeted by a panel were analysed and a one-sided Poisson test was performed to determine whether the observed number of error-corrected alternate reads deviates from the background error encountered by a panel (methods). For individual subclones a subclonal caller evaluates the presence or absence of ctDNA on a per-subclonal cluster basis. This information was integrated with primary-tumour informed cancer cell fraction (CCF) estimates made through ctDNA analysis at relapse using the ECLIPSE (Extraction of Clonality from Liquid BioPSiEs) tool (methods) to categorise the lung cancer relapse process as either monoclonal (a single clone seeding the metastasis), polyclonal monophyletic (multiple clones seeding metastasis from a single branch of the primary tumour phylogenetic tree) or polyclonal polyphyletic (multiple clones seeding metastasis from multiple primary tumour branches of the phylogenetic tree). ECLIPSE was also able to detect the presence of a clonal sweep whereby a subclone achieves 100% CCF at recurrence (methods).

**MRD calling threshold determination|** We assessed a pilot-cohort of 91 plasma samples from 10 patients to evaluate pre-defined P<0.1 and P<0.01 variant DNA detection algorithm thresholds. An error during patient specific panel (PSP) design for patient CRUK0297 in this pilot-phase of the project meant that 44 of 199 variants selected for PSP design were germline. This would confound ctDNA detection analyses of samples from this patient, therefore these variants were excluded from all analyses and only 155 variants were followed (manual removal of mis-targeted variants was only performed in this pilot-cohort patient, where germline variants were mistakenly targeted in non-pilot patients these were kept in analyses [methods]). For the remaining 9 patients, PSPs contained a median of 200 variants (94 to 200 variants). We noted 0 of 55 false positive ctDNA calls (defined as postoperative ctDNA positive calls in 5 non-recurrent patients) at both the 0.1 and 0.01 MRD P-value call thresholds (Extended Figure 2a). In 5 patients who did experience NSCLC recurrence, 1 of 13 postoperative MRD calls were made between P-values of 0.1 and 0.01, the remaining 12 calls were made at P-values <0.01 (Extended Figure 2b). In 10 preoperative cell-free DNA samples we observed 7 positive ctDNA calls, all calls were made at P-values <0.01 with P-values in the three ctDNA negative patients of 1, 1 and 0.7 (Extended Figure 2c). We were limited in our capability to assess assay false positive rate in the pilot cohort (n of only 55 postoperative samples from non-recurrent patients), therefore we generated 3157 *in-silico* simulated negative cfDNA samples from the pilot-samples (methods, DNA bases not targeted by a PSP were interrogated by the MRD caller algorithm and since we would not expect a ctDNA signal at these positions these were treated as negative samples). We noted a specificity of 99.3% versus 96.2% at <0.01 and <0.1 P-value thresholds respectively (Extended Figure 2d). Based on the observed specificity of >99% in simulation (Extended Figure 2d) and the minimal impact on false negative rate (1 of 20 ctDNA positive calls at the P-value <0.1 threshold would have been negative at the P-value <0.01 threshold, Extended Figure 2a-b) we advanced an MRD P-value threshold of <0.01 to further analyses.

**Analytical Validation of AMP ctDNA detection|** Analytical validation of the assay was first performed using PSPs targeting 50-variants (representing a clinic-ready version of the AMP MRD assay, Extended Figure 2e-h, Supplementary Table 3). In experiment one (LOD1) fragmented germline DNA was spiked into a wild-type background at concentrations ranging from 0.1 to 0.003% variant allele frequency (VAF) and different DNA inputs (ranging from 2ng to 80ng) were analysed for variant DNA signals using the 0.01 MRD P-value threshold described above (Extended Figure 2e, methods). Sensitivity of AMP to detect variant DNA using the MRD caller scaled with DNA input into the assay (Extended Figure 2e). At 30ng input 100% sensitivity (95 CI : 74 to 100%) was observed at 0.05% VAF, 92% sensitivity (95% CI: 64 to 100%) at 0.01% VAF and 100% sensitivity (95% CI: 74 to 100%) at 0.008% VAF; at the lowest VAF tested at 30ng input (0.003% VAF) 17% sensitivity was observed (95% CI: 2 to 48%, Extended Figure 2e). At the highest DNA input tested (80ng) 100% sensitivity was observed down to 0.008% VAF and at 0.003% VAF 25% sensitivity (95% CI: 6 to 57%) was achieved (Extended Figure 2e). In experiment 2 (LOD2), circulating tumour DNA from high ctDNA level samples was spiked into a wild type background (methods). At 10ng DNA input into the assay 100% sensitivity for ctDNA detection was achieved at 0.1% and 0.05% VAF and 75% sensitivity was achieved at 0.01% and 0.008% VAF (95% CI: 43 to 95%, Extended Figure 2f). At 60ng DNA input 100% sensitivity was observed at 0.005% VAF (95% CI: 63 to 100%, Extended Figure 2f). Specificity analyses were performed in 48 blank samples from healthy participants; all samples were negative for variant DNA demonstrating a specificity of 100% in analytical validation experiments (Extended Figure 2g, Supplementary Table 3).

Extended Figure 2h contains the observed variant DNA fraction for each of the spike-in experiments where variant DNA was detected, categorised by DNA input into the assay and experiment (LOD1 or LOD2). These analyses reveal that at higher DNA inputs (>10ng) the intended spike-in variant allele frequency was generally recapitulated by variant DNA fraction measurement by AMP. For example in LOD1, at 10ng DNA input the following mean (±SD) allele frequencies (AFs) were observed at 0.1%, 0.07%, 0.05%, 0.03% and 0.01% spike-in frequencies - 0.08% ± 0.01%, 0.06% ± 0.01%, 0.05% ± 0.02%, 0.03% ± 0.01% and 0.02% ± 0.01% respectively. At 80ng DNA input, the following mean AFs were observed at 0.008%, 0.006%, 0.005% and 0.003% spike-in frequencies - 0.007% ± 0.001%, 0.005% ± 0.002%, 0.004% ± 0.001% and 0.005% ± 0.002% respectively. We noted in these analyses that the observed AF tended to be less accurate and overestimated at DNA inputs <10ng where low spike-in frequencies were analysed. For example, at 0.01% spike-in in experiment LOD1, the measured AF in the single positive sample at 2ng input was 0.05% and at 0.008% spike-in, the mean measured AFs at 4ng and 5ng were 0.03% and 0.02% respectively. Additionally the standard deviation of observed AFs was the widest at 2ng input suggesting high variation in observed AFs (Extended Figure 2h). One possible reason for this is insufficient complexity (error-controlled depth) in low input samples to accurately quantitate mutant allele frequencies. On this basis, we excluded samples with <10ng input from ECLIPSE cfDNA analyses, given the inability to accurately quantify AF, which is integral in extracting CCFs from plasma (Supplementary note section ‘In vitro validation of subclone detection sensitivity using spike-in experiments’).

**Orthogonal validation of AMP ctDNA detection|** Orthogonal validation of AMP MRD caller accuracy in matched pre-operative samples from 30 TRACERx patients was performed with digital droplet PCR (ddPCR against clonal *KRAS* or *EGFR* variants known to be present in a patient’s primary tumor based on tumor exome data, Supplementary Table 4, methods). Pre-operative cfDNA input into the ddPCR assay was higher than cfDNA input into AMP assays (median cfDNA input into AMP 18.2ng [range 5 to 47ng] versus 19.1ng into ddPCR [range 5ng to 78ng], Wilcoxon Paired-Test P=0.014, Extended Figure 2i). To make assay agreement comparisons between ddPCR and AMP we used two ddPCR thresholds, a one mutant droplet threshold for a positive ddPCR call (recommended by the provider) and a two mutant droplet threshold for a positive ddPCR call (as previously published in NSCLC using this assay)[^1^](https://paperpile.com/c/TRxvNu/gcrhM). At one and two mutant droplet positive thresholds respectively, percentage positive agreement between AMP and ddPCR was 80% and 100% and percentage negative agreement was 85% and 76% (ddPCR the comparative method and AMP the test method, Extended Figure 2j). There was a significant association between AMP status and ddPCR status at both thresholds (Figure 2j, two-sided Fisher test P values of 0.001 and 0.003 for 1 mutant droplet ddPCR threshold and 2 mutant droplet respectively).

**Validation to assess targeting an increased mutation number|** Extended figure 2e-h describes the sensitivity of a 50-mutation AMP PSP. We also assessed the increase in sensitivity conferred by targeting more than 50 mutations given that a median of 126 clonal mutations were tracked across panels in this manuscript (experiment LOD3, Supplementary Table 5). To achieve this a single PSP was designed targeting 300 heterozygous SNPs and *in-silico* sub-sampling of this PSP was performed to assess 3 x 200, 3 x 100 and 3x 50 variant panel sensitivities across spike-in variant levels from 0% to 0.1% with 10ng DNA input (Extended Figure 2k, methods). Sensitivity at 10ng input improved when an increased number of mutations were tracked. For example at 0.01% spike-in sensitivity was 50% (95% CI: 12 to 88%) with 50 mutations, at 100 and 200 mutations, sensitivity increased to 67% (95% CI: 22% to 96%) and 100% (95% CI: 54% to 100%) respectively (Extended Figure 2k). All 18 analyses performed at 0% allele-frequency spike-in were negative for variant DNA using the 300 variant version and subsampled versions of this PSP.

**Subsampling analyses of TRACERx libraries to assess the effect of tracked mutation number on sensitivity|** We evaluated ctDNA detection sensitivity for each TRACERx plasma sample by calculating the minimum detectable allele frequency (MDAF) and then categorising achieved median MDAFs by cfDNA input (given the association between input and assay sensitivity described in extended figures 2e-f). MDAF was calculated by estimating the number of observed mutant reads which would be required to reach the pre-defined 0.01 MRD caller P value threshold, considering the total depth and expected background noise across all variants tracked. We randomly sub-sampled the tracked mutations in each sample (to 1 ,2, 5, 20, 30, 50, 75 and 150) and repeated our MDAF calculations in each case to determine the effect of the number of tracked mutations on our ctDNA detection sensitivity. We observed a stronger association between the number of tracked mutations and ctDNA detection sensitivity at lower assay inputs (Supplementary table 6). When tracking all 200 mutations used in the TRACERx panels we observed a 45% decrease in median estimated MDAF (from 0.009% to 0.005%) in samples with 5ng-10ng of cfDNA input when compared to 50 mutations (as used in our analytical validation experiments). This estimate is in line with the analytical validation data presented in Extended Figure 2k (10ng DNA input) providing confidence in the median MDAF estimates presented in Supplementary Table 6.

**Preoperative ctDNA detection in synchronous primary cases|** 9/187 patients had synchronous primary NSCLC at diagnosis, of which 5/9 had preoperative ctDNA detection. In 4/5 cases variants from both primaries were tracked yet only ctDNA from one tumour was detected. In 3 of these cases the largest primary tumour (CRUK0622) or the tumour with squamous histology in cases of synchronous adenocarcinoma and squamous cell carcinoma (CRUK0223, CRUK0555) led to ctDNA detection (Extended figure 3b).

**Volume sensitivity analysis|** To ensure the results pertaining to ctDNA detection biology were not primarily driven by higher tumour volume in ctDNA positive versus low-shedder cases, we excluded the bottom quartile of tumour volumes from low-shedding adenocarcinomas and repeated the transcriptomic and GISTIC analyses. This resulted in 970 significant genes and 20 significant cytobands, including 763/876 significantly overexpressed genes and 18/20 significantly altered cytobands as identified from the extended cohort (Extended figure 4i-k).

**CRUK0498 false positive analysis|** CRUK0498 was observed to have ctDNA positive plasma samples at 7 of 8 postoperative timepoints but did not experience clinical relapse (Extended figure 5b [left panel] displays AFs of individual mutations detected in cfDNA). Presumed lymphoid somatic (nonmalignant) mutation mistargeting in this case was identified through application of CRUK0498’s patient specific panel (PSP) to peripheral blood mononuclear cell (PBMC) DNA (buffy-coat) isolated alongside cfDNA, normal lung tissue DNA and control genome in a bottle (GIAB) DNA (Extended figure 5b [middle panel], methods). This analysis revealed that mutations detected at most timepoints in cfDNA (Extended figure 5b, left panel) were also detected in PBMC DNA and normal lung DNA, but were absent from control GIAB DNA (suggesting that they were not technical artefacts, Extended figure 5b [middle panel], Supplementary Table 19). These somatic, non-tumour associated mutations existed at high AFs in lysed tumour tissue (potentially due to the presence of lymphoid cells in this tissue, see below) but were below the 1% germline exome detection threshold in PBMC DNA (Extended figure 5b, middle and right panel). Therefore, the mutations were not called by the exome of germline blood used to perform germline subtraction, explaining why they were misclassified as tumour derived by tissue exome analyses and included in PSP design. For example, mutation *ATP2C1:T>G* (detected in 6 postoperative cfDNA samples, Extended figure 5b [left panel]) was present at an AF of 16.6% in tumour region 2 (Extended figure 5b [right panel]; yet max AFs in postoperative cfDNA and PBMC DNA were only 0.57% and 0.86% respectively (Extended figure 5b [left and middle panels]). Histological examination of CRUK0498’s tumour revealed an aggregate of lymphoid cells, potentially representing a source of these non-malignant mutations (Extended figure 5c). To address whether PSPs frequently mistargeted non-tumour-variants present in tumour tissue, we evaluated PBMC DNA from 19 additional patients using their PSPs. Application of the MRD detection algorithm to PBMC samples revealed no variant DNA detection (pan-library MRD detection algorithm P-values above 0.01, Extended figure 5d, Supplementary Table 19). At the level of individual mutations, 12 of 3621 (0.3%) PSP-targeted mutations were detected in PBMC DNA, only 2 / 12 mutations were present in preoperative cfDNA and not filtered by the MRD detection algorithm, suggesting that PSP mistargeting of non-tumour somatic mutations is a rare event (Extended figure 5e, Supplementary Table 20).

**ECLIPSE motivation & overview|** Clonal decomposition of tumour tissue is widely performed using whole exome or whole genome sequencing where coverage of single nucleotide polymorphisms (SNPs) is leveraged to determine tumour purity (defined in ‘Determination of ‘tumour purity..’ section below) and genome wide copy number states which are required to determine cancer cell fractions (CCFs) for each mutation and clone, representing the clonal composition of the sample[^2^](https://paperpile.com/c/TRxvNu/WX81b). These methods are not currently practical for low tumour purity plasma samples (<1%), which make up the vast majority of samples in the localised, minimal residual disease or early metastatic clinical settings, because sufficient coverage of SNPs for B-allele frequency separation for calling *de novo* copy number alterations and has not been demonstrated to be feasible in low purity (<1%) cfDNA samples where DNA input is also highly limited. Hence, we constructed an informatic tool ECLIPSE (Extraction of Clonality from Liquid BioPSiEs) to leverage the copy number and clonal status of each mutation from matched tissue sequencing with variant allele fractions from deep targeted sequencing of plasma to calculate the tumour purity and CCFs for each mutation and clone in the tumour mass shedding into the plasma at the time of blood collection (Extended Figure 7a) while accounting for background sequencing noise. For low tumour purity plasma samples, high confidence determination of the presence or absence of subclones is also critical to clonal composition estimates as the signal to background noise ratio will be much lower than in higher tumour purity tissue samples. ECLIPSE therefore uses expected background noise profiles to provide high confidence calls for the presence or absence of each clone, *i.e.* whether there is significantly greater observed signal than expected noise, for each clone in each plasma sample. A plasma specific method to measure, *de novo*, the size of the major subclone using measurements of somatic copy number alterations from low pass WGS has been recently published[^3^](https://paperpile.com/c/TRxvNu/NPXRJ). While this method is cost effective and does not require tissue sequencing, a breakdown of the clonal composition for specific subpopulations is not provided and plasma samples with tumour purity <10% were discarded from analysis due to a greater than acceptable influence of noise in subclonality estimates. In contrast we were able to include samples with at least >0.1% tumour content by using our deep targeted sequencing approach and ECLIPSE. This threshold was validated using *in vitro* spike-in experiments. While our 2017 publication[^4^](https://paperpile.com/c/TRxvNu/MoOdY) described detection of specific subclonal lineages in ctDNA, we were not able to calculate cancer cell fractions (CCFs) without ECLIPSE, preventing or limiting several areas of investigation including measurements of clonal dynamics, complete clonal sweeps, subclonal expansions and relapse seeding patterns, where we focus our attention in this work. We have also tracked a larger fraction of subclones (88%) and many more mutations per subclone (median of 4) which ECLIPSE leverages by combining the signal across all mutations in the same subclone for more sensitive subclone detection.

**Determination of ‘tumour purity’ in plasma samples and contrast with ‘ctDNA level’|** In the circulating tumour DNA field the term “ctDNA fraction” is used conceptually to refer to the percentage of DNA molecules derived from tumour cells in a liquid biopsy and is used as a proxy for tumour burden in a patient. Is it often measured using the average variant allele frequency (VAF) of mutations in a sample. Non-mutated tumour derived cfDNA molecules are usually ignored in measurements of ctDNA fraction as they are often not possible to detect. In this work we use the term ‘clonal ctDNA level’ to refer to the average VAF of clonal mutations which should be present in all tumour cells, hence representing an improved measure of tumour burden than total average VAF. In contrast in the clonal deconvolution field, calculation of “tumour purity” (also referred to as “cellularity” or “aberrant cell fraction”) is essential to clonal composition estimates and refers to the percentage of cells in a sample which are tumour derived, or more precisely as the percentage of all cells from which the DNA molecules sequenced derive that are tumour cells, rather than non-tumour cells (Extended figure 7a). This is also the same measure made in cfDNA by IchorCNA[^5^](https://paperpile.com/c/TRxvNu/cBhJw) termed ‘Tumour fraction’ using copy number aberrations and broad sequencing such as shallow WGS. To demonstrate the difference between this measure and average VAF imagine a diploid tumour without somatic copy number aberrations, with one copy of each somatic mutation, where tumour cells constituted 20% of all cells and all mutations are clonal, we would expect the average VAF (referred to as ‘ctDNA fraction’/’ctDNA level’ in this work in plasma) to be 10%, as at each mutated locus in the tumour cells there is one mutated copy and one wild type copy. However in this case the ‘tumour fraction’ or ‘tumour purity’ correctly estimated by IchorCNA or ASCAT[^6^](https://paperpile.com/c/TRxvNu/squUk) would be 20%. This example also demonstrates that when calculating the cancer cell fractions (CCFs) from VAFs of subclonal mutations during clonal deconvolution, both the number of mutated and wild type copies must be known as well as the percentage of cells from which the sequenced DNA is derived which are tumour cells (‘tumour fraction’ or ‘tumour purity’). The average VAF of mutations (often referred to as ‘ctDNA fraction’ in plasma or as ‘ctDNA level’ in this work) does not provide this information as the VAF is affected by both the wild type and mutant copy number at the variant loci and the clonality of the variants. We must therefore calculate the percentage of cells from which plasma DNA molecules are derived which are tumour cells rather than non-tumour (e.g. haematopoietic) cells, which we referred to in this manuscript as ‘ctDNA tumour purity’ accounting for copy number aberrations and the variants’ clonality in the tumour. The relationship between ctDNA purity, clonal ctDNA level and the average copy number of tracked mutations is shown in Extended Figure 7e. Calculation of tumour purity from ctDNA data has also been performed previously with standard informatic methods[^2^](https://paperpile.com/c/TRxvNu/WX81b) leveraging whole genome or whole exome sequencing data of plasma. These methods are normally applied for clonal deconvolution of tissue samples and are appropriate for ctDNA samples of high ctDNA tumour purity (>10%) but are not practical for low ctDNA tumour purity plasma samples (<1%) which make up the majority of samples in the localised, minimal residual disease or early metastatic clinical settings (see section above ECLIPSE motivation & overview). The equation which describes the relationship for each mutation between the CCF, VAF, number of mutated DNA copies per tumour cell, total number of DNA copied at the mutated locus and the ‘tumour purity’ of the sample is shown Extended Figure 7c. Using matched tumour tissue samples several of these factors can be calculated and can form probable estimates for these same factors in the tumour at the time of plasma sampling (see section below on stability of mutant copy number from surgery to relapse). For mutations which are found to be clonal in tissue sequencing, the vast majority are likely to remain clonal in subsequent tissue samples and hence these mutations can be presumed to have a CCF of 1 (100%). We can therefore reduce and rearrange the equation shown in Extended Figure 7c to that in Extended Figure 7b for mutations expected to be clonal and calculate a ctDNA tumour purity estimate for each clonal mutation, leveraging the copy number estimates from tissue and VAF in plasma. The mean of these values can then be used as a final, sample-level estimate for the ctDNA tumour purity of the sample. We show in Figure 4a that using estimates of clonal mutation status from only a single tumour region (i.e. including some subclonal variants which may have clonal illusion in the selected region) with ECLIPSE still provides biologically meaningful results. VAF measured in plasma is also corrected for background noise in ECLIPSE (Methods: Stepwise description of ECLIPSE). A similar methodology to this has been used as a quality control for tumour purity and ploidy estimates using single nucleotide polymorphisms with methods such as ASCAT[^6^](https://paperpile.com/c/TRxvNu/squUk) for tumour tissue samples[^7^](https://paperpile.com/c/TRxvNu/gV7vb) but has not been applied to clonal SNVs in deep targeted sequencing data, which lacks SNP coverage, for *de novo* estimates of tumour purity when the ploidy is known.

**Stability of mutant copy number alterations over time|** CCF calculation from VAF in low fraction ctDNA samples mostly involves correction for mutant multiplicity in tumour cells, as only a very small proportion of wild type DNA molecules are tumour derived. Copy number information is provided to ECLIPSE from tissue samples. Copy number instability is a hallmark of cancer and modifications to copy number at mutated loci between time of tissue sampling and plasma sampling could introduce noise into CCF estimates. We quantified the changes in mutation multiplicity for clonal mutations between matched primary tumour and relapse tissue in TRACERx (Extended Figure 7d). We found on average each mutation’s multiplicity estimate is increased or decreased by 7.7% at relapse compared to estimates at surgery from M-seq, without a consistent bias toward either gain or loss. These data show that mutation copy number at surgery provide much better predictions than chance for mutation copy number at other timepoints and hence correction for these will improve CCF estimates. Additionally, while changes in multiplicity will introduce some noise in mutation CCF estimates, each clone is tracked by a median of 4 mutations, most of which do not change multiplicity between surgery and relapse, hence the effect on clone level CCFs will be small in comparison to other sources of noise inherent to CCF estimates in this and other datasets. It should also be noted that the presumption of equal multiplicity is also required for the same mutation in different subpopulations with a sample using standard clonal deconvolution methods.

**Minimal detectable CCF estimates for each subclone and sample evaluability thresholds|** To quantify our limits of detection of CCF in each sample and subclone, ECLIPSE calculates the number of supporting reads for all mutations in each subclone that would be required for a positive clone detected call (P<0.01 threshold) based on the number of expected background noise reads using the qpois function in R (methods). The sample level minimal detectable CCF estimates and their relationship with clonal ctDNA level are shown in Extended Figure 8a. The minimal detectable CCF varies over several orders of magnitude with the ctDNA tumour purity. Samples with 0.09% clonal ctDNA level on average had a minimal detectable CCF of 20% whereas samples of high purity (>5%) had very low estimated minimal detectable CCF of <0.5% which represents greater sensitivity for small subclones that has been achieved previously with SNP based subclonal deconvolution methods[^2^](https://paperpile.com/c/TRxvNu/WX81b) on samples of similar tumour purity. The changes in minimally detectable CCF are shown over time for each patient and all ctDNA positive samples (Extended Figure 8b). These estimates were used to determine a clonal ctDNA level threshold for high quality subclone detection of 0.1% where on average plasma samples had a minimum detectable CCF of 20%, which we validated using spike in experiments (see supplementary note section ‘In vitro validation of subclone detection sensitivity using spike-in experiments’). 64% of ctDNA positive plasma samples had a clonal ctDNA level of at least 0.1% in this study. A greater fraction of samples and patients at relapse could be analysed using this threshold compared to with thresholds required for standard subclonal deconvolution methods using WES/WGS (at least 10% tumour purity, Extended Figure 8c-d). This threshold and a minimum DNA input of 10ng was used as a sample inclusion criteria for clonality-based analyses including validation of ECLIPSE CCFs compared to tissue-based CCF measurements (Extended figure 9a), distinguishing clonal and clonal illusion mutations using plasma in preoperative samples (Figure 4a), pre-operative CCF measurements of metastatic subclones and subsequent clonal sweeps at recurrence (Figure 4b and Extended Figure 10g-i) and determination of metastatic dissemination type (Figure 5, Extended Figure 10d-f). Patients with at least 1 postoperative plasma sample of >0.1% clonal ctDNA level and >= 10ng DNA input had clonal structure from all plasma samples and matched tissue samples depicted over time (Figure 5, Supplementary Figure 1). For distinguishing clonal and clonal illusion mutations using plasma in preoperative samples (Figure 4a) a lower threshold could also have been used given each mutation was called in surgical tissue samples and is therefore known to be present at the time of pre-operative plasma sampling, which would allow extraction of this data for a greater fraction of patients. In this case the absence of an apparently clonal mutation with a minimal detectable CCF of 50% will indicate a CCF < 50% and a clonal illusion. We repeated the analysis of clonal illusion across a range of evaluability thresholds of 0.1%, 0.05%, 0.025% and 0.015% ctDNA level equivalent to 10%, 30%, 50% and 75% minimal detectable CCFs based on best fit line in Extended Figure 8a and allowed inclusion of 42%, 48%, 53% and 60% of all preoperative samples respectively as evaluable, finding that clonal illusion mutations had significantly lower plasma CCFs than true clonal mutations with P<0.001 in each case.

**In vitro validation of subclone detection sensitivity using spike-in experiments|** We leveraged sequencing of spike-in mutations with known allele frequencies to construct *in silico* 76,263 subclones with varying cancer cell fractions, clonal ctDNA levels, DNA input and numbers of tracked mutations derived from ground truth. We applied ECLIPSE to each of these subclones to estimate ECLIPSE’s subclone detection sensitivity in our data across these parameters (methods: Validation of subclone detection rates using our data and ECLIPSE, Extended figure 8e). 93% of ctDNA positive plasma samples had >10ng of DNA input in the TRACERx samples and the median number of mutations tracked per subclone was 4. At 10ng of DNA input and 4 mutations tracked, we observed a mean subclone detection rate of 100% (95% CIs: 100-100%, 2530 subclones across all experiments tested and all detected) at 33% CCF and 0.1% clonal ctDNA level and 79% (95% CIs: 74-84%, 2905 subclones across all replicates tested) at 10% CCF and 0.1% ctDNA level. We fitted a non-linear asymptotic regression model to these data which predicted a detection rate of 94% at our *in silico*-estimated 20% minimal detectable CCF threshold and 0.1% clonal ctDNA level. We observed a significant drop off in sensitivity for small subclones when <10ng of DNA was inputted (45% detection for 20% CCF subclone at 0.1% clonal ctDNA level and 5ng input) and therefore added an additional threshold of >=10ng of input DNA to consider a sample of ‘high subclone sensitivity’ (which previously only required >0.1% clonal ctDNA level). Overall however, we found good agreement between our validation data using in vitro spike-in samples and our initial *in silico* minimal detectable CCF calculations (94% sensitivity at the 20% minimal detectable CCF, 0.1% ctDNA level threshold) with a remaining mismatch when comparing to our pre-operative subclone detection rates (39% detection of 20% CCF tissue subclones at 0.1% clonal ctDNA level).

**Undetected phylogenetic clones with detected daughter subclones|** We noted in a minority of cases (CRUK0617, CRUK0094, CRUK0245,CRUK 0087, CRUK0543, CRUK0674, CRUK0769 and CRUK0762) that a subclone was not detected by ECLIPSE despite daughter clones in the inferred phylogeny being detected. This was possible as the phylogeny was built entirely using the tissue sequencing data for consistency between this and other TRACERx 421 manuscripts[^8–10^](https://paperpile.com/c/TRxvNu/a3dQZ+UOzMw+53pik), without accounting for data from the ctDNA samples. There are two possible explanations for this phenomenon. Firstly, the undetected clone is present and we were unpowered to detect it which is likely for subclones with few mutations tracked (e.g. only two mutations were tracked from the five mutations present in subclone *d* in CRUK0617, compared to an average of 5 tracked mutations in its daughters, Figure 5a) and in samples of low clone purity (i.e. ctDNA purity multiplied by clone CCF, e.g. a clone purity of <0.02% for subclone *e* in the preoperative sample of CRUK0543 where its daughter was detected, Figure 5a). Secondly, the phylogeny inferred using tissue sequencing may be incorrect. In fact, while we use the default phylogeny from our tree building method (CONIPHER[^11^](https://paperpile.com/c/TRxvNu/3mxc0)), alternative phylogenies are often also provided. This is the case for CRUK0617 where all detected daughter clones of subclone *d* may instead be daughters of other clones in the phylogeny. As *de-novo* copy number aberrations could not be inferred from our AMP-PSP deep sequencing of plasma, we did not attempt to alter the phylogeny based on the ctDNA data.

**Validation of ECLIPSE CCF estimates in pre-operative plasma|** ECLIPSE estimation of CCFs in pre-operative plasma correlated directly with CCF estimates from multi-region tissue exomes of tumours sampled at surgery (pearson’s r = 0.78, P<0.001, m = 1, Extended Figure 9a) using plasma samples with a median of 0.9% clonal ctDNA level. These results validate ECLIPSE estimates of CCF and hence metastatic dissemination class calls in the relapse setting. Copy number unaware estimates of CCF from plasma consistently underestimated tissue based CCFs unlike when using ECLIPSE (methods, Extended Figure 9b). Discrepancies in this correlation are likely to at least partially derive from differences in the CCF (i.e. clone size) of subclones in the tumour tissue sampled and subject to multiregional exome sequencing (usually <10% of the total tumour mass) and the CCF (i.e. clone size) in the total tumour mass from which the plasma DNA may derive (see section ‘Evidence for detection bias causing CCF overestimation for small subclones in tumour tissue sampling’ below).

**Concordance between subclones detected in relapse cfDNA and relapse tumour tissue|** In 26 patients with both metastatic tissue and high subclone sensitivity post-surgical cfDNA 50/51 [98%] tissue relapse subclones were also detected in post-operative cfDNA, whereas 20/201 [9%] subclones absent from recurrence tissue were detected (Extended Figure 10b) representing a significant enrichment of relapse tissue detected clones also detected in relapse ctDNA (*P* < 0.001, Fisher’s exact test). These subclones were also significantly more likely to exhibit plasma CCFs significantly less than 100% at recurrence (P<0.001, Fisher’s exact test), suggesting these subclones were present in only a subpopulation of relapsing tumour cells (Extended Figure 10c, methods).

**Evidence for detection bias causing CCF overestimation for small subclones in tumour tissue sampling|** In TRACERx we undertake multiregional sampling of tumours where DNA is extracted from a median of 3 2mm^3^ samples of tissue per tumour. In many cases this means the large majority of the tumour mass is not extracted for DNA sequencing and therefore we expect some remaining sampling bias in our data in relation to the total tumour mass (Extended Figure 9g). Many clones will remain unsampled and those which are sampled will be subjected to a detection bias effect expected in spatially constrained sampling called the ‘winner’s curse’ where clones discovered within samples are expected to be oversampled, on average, relative to the total tumour. This is because while we expected there to be both over and undersampled clones, many of the undersampled clones are not present in the sampled tissue and hence are not discovered. Therefore, in the clones which we are able to discover, we would expect on average clones to be oversampled, i.e. have a higher cancer cell fraction (CCF) across all the tissue which we have extracted compared to their cancer cell fraction across the tumour as a whole. Large subclones which are spread across many regions should be less prone to this sampling bias whereas the smaller clones, unique to single samples, should be more heavily oversampled. This effect is discussed by the PCAWG consortium publication for low CCF subclones[^12^](https://paperpile.com/c/TRxvNu/Wah2G). Plasma ctDNA will not be derived solely from the same tumour tissue regions that were subject to DNA extraction and hence subclones discovered using tissue will not be subject to the winner’s curse phenomenon in ctDNA-derived CCFs. Therefore, we would expect to detect lower subclonal CCFs in the plasma, on average, than in the tissue, particularly for subclones only present in one tissue region, where the winner’s curse would be most evident. Indeed, we do observe that subclones unique to one region of tumour tissue have a much lower CCFs in plasma compared to tissue, suggesting sampling bias is causing an overestimation of the total subclone size for smaller subclones in tumour tissue due to the winner’s curse effect (Extended Figure 9d). In contrast, we observe a far smaller difference between tissue and plasma CCFs in subclones which are spread across several regions (Extended figure 9d). We also observed an even greater difference in plasma vs tissue CCFs in larger tumours, where a smaller proportion of the total tumour mass is sequenced despite increased number of tumour samples, consistent with increased sampling bias and winner’s curse in tumour tissue CCFs for cases that are more sparsely sampled (Extended figure 9e). When splitting detection rates for 20% CCF subclones by whether or not they were present across multiple tumours regions we found that detection rates were several fold higher for 20% CCFs subclones spread across regions compared to 20% CCF subclones unique to a single region at 0.1% clonal ctDNA level (Extended figure 9f). This result is consistent with our hypothesis that sampling bias in measurements of tissue CCF drives lower than otherwise expected subclone detection rates in plasma where by only 39% of 20% CCF subclones were detected in pre-operative plasma of 0.1% clonal ctDNA level (Extended Figure 9c) whereas using our *in vitro* spike-experiments we estimated a 94% sensitivity for such subclones (Extended Figure 8e). As all the subclones tracked were discovered in the primary tumour we also would not expect a winner’s curse / sampling bias effect when sampling the same subclones at relapse with either tissue or ctDNA. Consistent with this, our detection sensitivity in postoperative ctDNA was high for subclones present in relapse tissue. We could detect 50/51 (98%) of the tracked subclones present in relapse tissue also in post-operative ctDNA (Extended Figure 10b in the 26 patients with both evaluable ctDNA and a relapse tissue sample). This prominent winner’s curse effect suggests that many of the subclones we discover in tumour tissue are small and either completely or mostly localised to the tissue regions which we sample. This in turn suggests that there are a large number of small, localised subclones in the tumour which remain undiscovered as they are only present in the unsampled tumour tissue, despite use of multiregional sequencing.

**Identification of complete clonal sweeps at recurrence|** In 18/42 (43%) patients with a high subclone sensitivity postoperative plasma sample available, we estimated that subclones tracked from the surgically resected tumour had undergone a complete clonal sweep at recurrence, where a subpopulation of cells expands to become clonal across all tumour sites (methods). This phenomenon led to increased clonal tumour mutation burden (median 14% increase; Extended figure 10g-h) and increased clonal neoantigen burden at recurrence (median of 11% increase; Extended figure 10i) which we have previously linked to the efficacy of immunotherapy[^13^](https://paperpile.com/c/TRxvNu/NeME6). Only 3/18 patients contained a known subclonal driver involved in the clonal sweep including mutations in *SMARCA4* (p.D881A & p.G1194E), KMT2D (p.G4182), *CTNNB1* (p.S37C), and an amplification in *MDM4*.

**Example of treatment-associated clonal evolution|** Patient CRUK0484 underwent plasma sampling on Day 55 (D55) after surgery and subclone *a* (Figure 5c), found in the primary tumour and surgically resected lymph node, was detected in cfDNA (clone detection *P*=6.9x10-5). This subclone receded following four cycles of adjuvant chemotherapy with carboplatin and vinorelbine (clone detection P=1, minimally detectable CCF = 0.4%, Figure 5c). Patient CRUK0484 relapsed with rib metastasis and on D147 post-surgery, clone *c* from a different phylogenetic branch dominated the tumour mass with 73% CCF detectable in plasma. This subclonal expansion pre-dated disease progression (rib, hilar lymph-nodes, liver and scapular bony metastases) which did not respond to palliative carboplatin and gemcitabine (administered D232 to 260). The phylogenetic branch containing clone *c* was not detected in rib and scapular bone relapse biopsies taken on D147 and on D379 respectively, therefore this subclone may have been present in the non-bone metastatic sites (which did not undergo tissue sampling). After the patient received immunotherapy (Nivolumab) clone *c* receded and clones from a different branch of the phylogenetic tree (*e.g*. clone *b*) expanded. This subclonal shift coincided with progression of the scapular metastasis and response or stable disease at rib, liver, spleen and lymph-node metastatic sites on imaging performed at D398 post-surgery. The immunotherapy resistant tumour branch was dominant in scapular metastasis tissue taken at D379 post-surgery, where the lineage including the expanding clone *b* was found (Figure 5c).

**References**

1. [Isaksson, S. *et al.* Pre-operative plasma cell-free circulating tumor DNA and serum protein tumor markers as predictors of lung adenocarcinoma recurrence. *Acta Oncol.* **58**, 1079–1086 (2019).](http://paperpile.com/b/TRxvNu/gcrhM)

2. [Tarabichi, M. *et al.* A practical guide to cancer subclonal reconstruction from DNA sequencing. *Nat. Methods* **18**, 144–155 (2021).](http://paperpile.com/b/TRxvNu/WX81b)

3. [Lakatos, E. *et al.* LiquidCNA: Tracking subclonal evolution from longitudinal liquid biopsies using somatic copy number alterations. *iScience* **24**, 102889 (2021).](http://paperpile.com/b/TRxvNu/NPXRJ)

4. [Abbosh, C. *et al.* Phylogenetic ctDNA analysis depicts early-stage lung cancer evolution. *Nature* **545**, 446–451 (2017).](http://paperpile.com/b/TRxvNu/MoOdY)

5. [Adalsteinsson, V. A. *et al.* Scalable whole-exome sequencing of cell-free DNA reveals high concordance with metastatic tumors. *Nat. Commun.* **8**, 1324 (2017).](http://paperpile.com/b/TRxvNu/cBhJw)

6. [Van Loo, P. *et al.* Allele-specific copy number analysis of tumors. *Proc. Natl. Acad. Sci. U. S. A.* **107**, 16910–16915 (2010).](http://paperpile.com/b/TRxvNu/squUk)

7. [Jamal-Hanjani, M. *et al.* Tracking the Evolution of Non–Small-Cell Lung Cancer. *N. Engl. J. Med.* **376**, 2109–2121 (2017).](http://paperpile.com/b/TRxvNu/gV7vb)

8. [Maise Al Bakir, Ariana Huebner, Carlos Martinez Ruiz, Kristiana Grigoriadis, Thomas B. K. Watkins, Oriol Pich, David A. Moore, Selvaraju Veeriah, Sophia Ward, Joanne Laycock, Diana Johnson, Andrew Rowan, Maryam Razaq, Mita Akther, Cristina Naceur-Lombardelli, Sonya Hessey, Michelle Dietzen, Emma Colliver, Alexander M. Frankell, Emilia Lim, Takahiro Karasaki, Christopher Abbosh, Crispin T. Hiley, Mark S. Hill, Daniel Cook, Gareth Wilson, Allan Hackshaw, Nicolai J. Birkbak, Simone Zaccaria, the lung TRACERx consortium, Mariam Jamal-Hanjani, Nicholas McGranahan, Charles Swanton. TRACERx: The evolution of metastases in non-small cell lung cancer. *Nature in press*.](http://paperpile.com/b/TRxvNu/a3dQZ)

9. [TRACERx consortium. Genomic-transcriptomic evolution in lung cancer and metastasis. (2022).](http://paperpile.com/b/TRxvNu/UOzMw)

10. [Al, F. et. The evolution of lung cancer and impact of subclonal selection in TRACERx. *Nature*.](http://paperpile.com/b/TRxvNu/53pik)

11. [Kristiana Grigoriadis, Ariana Huebner, Abigail Bunkum, Emma Colliver, Alexander M. Frankell, Mark S. Hill, Kerstin Thol, Nicolai J. Birkbak, Charles Swanton, Simone Zaccaria, Nicholas McGranahan. CONIPHER: a computational framework for scalable phylogenetic reconstruction with error correction. *Nature pre-print*.](http://paperpile.com/b/TRxvNu/3mxc0)

12. [Dentro, S. C. *et al.* Characterizing genetic intra-tumor heterogeneity across 2,658 human cancer genomes. *Cell* **184**, 2239–2254.e39 (2021).](http://paperpile.com/b/TRxvNu/Wah2G)

13. [Litchfield, K. *et al.* Meta-analysis of tumor- and T cell-intrinsic mechanisms of sensitization to checkpoint inhibition. *Cell* **184**, 596–614.e14 (2021).](http://paperpile.com/b/TRxvNu/NeME6)
